# Global, regional, and national characteristics and incidence of sudden death from 1948 to 2022: a systematic review and modelling study

**DOI:** 10.7189/jogh.16.04222

**Published:** 2026-07-31

**Authors:** Shuxiao Pan, Xiaoqing Huang, Xiumin Wu, Buxian Chen, Xiaoling Su, Ling Fang, Tingting Dai, Pi Guo

**Affiliations:** 1 Department of Clinical Medicine, Shantou University Medical College, Shantou, China; 2 Department of Pharmacy, Cancer Hospital of Shantou University Medical College, Shantou, China; 3 Department of Preventive Medicine, Shantou University Medical College, Shantou, China

## Abstract

**Background:**

Sudden death (SD) poses great health and social challenges worldwide. There are a few studies investigating the epidemiological characteristics and disease burden of SD globally. The study aimed to assess the epidemiological characteristics and global burden of SD in different population and regional groups, and to determine the incidence of SD and its influencing factors.

**Methods:**

We conducted a systematic review and spatiotemporal analysis to estimate global, regional, and national SD incidence. A comprehensive search was conducted across four databases, including PubMed, Embase, the Cochrane Library and Web of Science, between 1 July 2003 and 30 June 2023. The included studies reported the incidence of SD in the general population of a country or region, or provided data on which the incidence could be calculated. We utilised a Bayesian hierarchical linear mixed model to estimate global, regional, and national SD incidence and to describe the characteristics.

**Results:**

Our search identified 9797 records, with 108 studies meeting our inclusion criteria. The incidence of SD was significantly different among super-regions. South Asia was estimated to have the highest incidence for 4.01 cases (95% confidence interval (CI) = 1.40, 7.51) per 100 000 person-years on a log-transformed basis, followed by South East Asia, East Asia, and Oceania. Among them, Guyana showed the highest estimated incidence of 5.06 cases (95% CI = 2.78, 8.20) per 100 000 person-years, followed by India of 4.89 cases (95% CI = 2.08, 7.76) per 100 000. Sub-Saharan Africa had the lowest estimated incidence, at 1.79 cases (95% CI = –1.83, 3.98) per 100 000 person-years. The temporal trends in SD incidence rates exhibited remarkable heterogeneity across regions. The downward trend was most pronounced in high-income countries, while Sub-Saharan Africa maintained a stable high incidence rate. Conversely, Latin America and the Caribbean exhibited continuous growth, with the Andean sub-region showing the most striking trend. Risk factors associated with SD include diabetes, hypertension, hyperlipidaemia, and heart disease.

**Conclusions:**

This study provides a comprehensive assessment of the global incidence of SD. The findings emphasise the need for targeted strategies to effectively reduce the burden of SD

**Registration:**

PROSPERO: CRD42023432992.

Sudden death (SD) is widely defined as an unexpected death occurring within one hour or within 24 hours in the absence of witnesses [1]. Generally, SD is categorised into traumatic and non-traumatic subtypes, with sudden cardiac death (SCD) representing the predominant aetiology of non-traumatic SD [2]. The World Health Organization (WHO) reported that cardiovascular diseases (CVD) remain a major threat to global health, accounting for approximately 17 million annual deaths worldwide [3]. Contemporary research has quantified the SCD incidence across diverse populations in multinational studies. Notably, SCD accounts for 40–50% of CVD mortality, representing approximately 3.7 million deaths each year [4]. These findings not only reveal the current severity of SD but also underscore its significance as a critical global public health challenge.

In recent decades, there have been remarkable changes in the epidemiology and disease spectrum of CVD. From 1990 to 2019, global CVD cases increased significantly, with annual mortality rising from 12.1 million (95% uncertainty interval = 11.4, 12.6 million) to 18.6 million deaths [4]. In contrast, another research demonstrated that the age-adjusted mortality rate of heart disease has declined in some developed regions, such as Japan, Australia, and New Zealand [5]. The burden of SCD is particularly pronounced in North America and Europe, affecting an estimated 50 to 1000 individuals per 100 000 person-years annually. The epidemiological characteristics of SCD are strongly influenced by age, gender, genetic predisposition, and environmental factors [6,7]. For instance, studies have demonstrated the significant international variations in age-standardised mortality rates of CVD, particularly before the age 80–84 years, where the incidence and mortality rates among males are three times higher than those for females [8,9]. Although CVD mortality has declined in high-income countries and some middle-income countries, no clear changes have yet been observed in certain low- and middle-income regions. Limited healthcare resources and prevalent risk factors like hypertension, obesity, and smoking further exacerbate the burden of SD, leading to persistently high incidence rates [10,11]. This situation imposes substantial pressure on healthcare systems, which increases the financial burden on families and society as a whole [10]. Although existing studies have described the regional epidemiology and disease burden of SD, comprehensive investigations examining the full spectrum of SD and its global disease burden remain limited. Therefore, an in-depth investigation into the global epidemiology of SD and its impact on public health and socioeconomic factors is urgently warranted.

At present, epidemiological data on SD are mainly confined to specific countries and regions worldwide, lacking a systematic and comprehensive assessment. Although SD has received increasing attention, existing studies have mainly focused on regions such as Europe, South Asia, and the USA. Information on other regions, especially low- and middle-income countries, remains scarce [12]. Furthermore, existing studies are relatively scattered, which limits a comprehensive understanding of the global trends and characteristics of SD. There is an urgent need for further research into the global and regional epidemiology of SD to alleviate the social burden of premature mortality caused by the disease [12].

Therefore, we aimed to conduct a systematic assessment of the epidemiological characteristics and disease burden of SD across countries and regions worldwide. The main objective was to comprehensively investigate the demographic characteristics, spatiotemporal distribution, and temporal trends of SD, considering dimensions such as age, gender, economic status, and sociocultural factors, and to conduct a multidimensional comparison of SD incidence and disease burden across population groups and regions. Overall, we aimed to provide scientific evidence for the effective formulation of public health policies and targeted preventive measures. We anticipate that this research will draw broader attention to the disease burden associated with SD and promote the implementation of effective interventions to reduce CVD mortality, thereby enhancing overall population health.

## METHODS

### Search strategy and selection criteria

We followed the PRISMA guidelines for conducting systematic reviews [13]. We conducted a comprehensive analysis to assess the epidemiology of SD and trends in disease burden at national, regional, and global levels. We systematically searched PubMed, Embase, Cochrane Library, and Web of Science, with the search period ranging from 1 July 2003 to 30 June 2023. The included literature was limited to English-language papers. We used free text keywords and thesaurus terms ‘sudden death,’ ‘epidemiology,’ ‘prevalence,’ ‘incidence,’ and ‘mortality’ to search for relevant literature (Table S1 in the **Online Supplementary Document**).

Two independent reviewers (CBX and SXL) applied the pre-established inclusion criteria to screen titles and abstracts, with any discrepancies resolved through discussion with a third researcher. Included studies were required to report SD cases, along with epidemiological and disease-burden data. Eligible study participants were required to meet the WHO definition of SD – death occurring within 24 hours of symptom onset [14]. We excluded non-human studies and those lacking specific SD population data.

We employed Rayyan (Rayyan Systems, Inc., Cambridge, Massachusetts, USA), a systematic review tool, to eliminate duplicate studies and to conduct a rigorous screening of titles and abstracts and full texts to ensure that only the most relevant studies were included [15]. Three authors (PSX, HXQ, and WXM) evaluated full texts. The eligible papers were evaluated strictly against the inclusion and exclusion criteria, and those that met them were selected for data extraction. Each step of the study selection process and the reasons for exclusion were documented. For studies with multiple publications or duplicates, only the most recent reports with comprehensive results were retained. This study was registered with the International Prospective Register of Systematic Reviews under registration number CRD42023432992 (Table S2 in the **Online Supplementary Document**).

### Data extraction and quality assessment

Five authors (PSX, HXQ, WXM, CBX, and SXL) independently completed the data extraction using a pre-designed table. We extracted relevant information on demographic characteristics from the included studies, and study results were converted into a standardised data extraction format. The gathered data items encompassed general information (publication year, geographical location, lead author’s name, journal title, and the digital object identifier), characteristics of target population (sample size, age, gender, SD year, season, race, body mass index (BMI), marital status, occupation, and SD family history), disease incidence and outcomes (cause of SD, state before SD, and relevant risk factors). To minimise bias, our team resolved disagreements by discussing with senior researchers.

We assessed all included studies for risk of bias using three independent Joanna Briggs Institute critical appraisal tools tailored to cross-sectional, case-control, and cohort studies, respectively [16]. The studies were categorised as high, moderate, and low risk of bias based on the overall quality of study design, methodology, and reporting of results. The results of the systematic assessment did not involve pooling estimates through a meta-analysis. For incidence, target population characteristics, and outcome, we systematically combined qualitative and descriptive statistical analyses.

### Data analysis

The primary outcome variable reported in this study was the incidence of SD, expressed as per 100 000 person-years. Descriptive summaries of the incidence rates of SD were either directly reported in the original studies or derived through our calculations. Specifically, we extracted the incidence rates and corresponding 95% confidence intervals (CIs) directly from the original studies, when available. For studies that did not report 95% CIs, we derived estimates using established statistical modelling techniques. Specifically, we constructed our estimates by utilising the available SD counts and corresponding person-years at risk. When data could not be extracted directly from the original studies, we utilised the WebPlotDigitizer tool (Automeris LLC, Austin, Texas, USA) to extract the relevant data points from the published graphs. The research results are stratified by country to facilitate detailed comparative assessments. Additionally, when the study data were available, we applied a random-effects model using the DerSimonian-Laird estimator for between-study variance, with variance correction implemented via the Hartung-Knapp-Sidik-Jonkman method, to summarise the incidence rates across different periods for temporal trend analysis [17]. Therefore, based on the midpoint year of each case collection period, we divided the research into five time periods: 1971–1980, 1981–1990, 1991–2000, 2001–2010, and 2011–2022.

We implemented a Bayesian hierarchical linear mixed model to estimate the incidence of SD at the super-region, region, and country levels. The model enables clear descriptions of spatial patterns, time trends, and local trends for each country [18]. The spatial random effect term in the model captures regional variation through a conditional autoregressive prior and assesses spatial heterogeneity using comprehensive ecological data at the regional level, with disease risk factors incorporated as standardised spatial covariates [18]. In the Bayesian hierarchical model, the log-transformed incidence of SD was used as the outcome variable to satisfy the basic assumption of linearity. Following previous studies [19], we classified countries using the Global Burden of Disease classification system [20], grouping 185 countries into 21 regions, which were further grouped into seven super-regions (Table S4 in the **Online Supplementary Document**). The classification followed the spatial-geographic structure and income levels across countries and regions. Differences in the distribution across age groups, data sources, incidence estimates, diagnostic methods, and other factors may contribute to substantial heterogeneity in predictions of global SD incidence. Consequently, the Bayesian computational framework included four random effects (global, super-regional, regional, and national) and three fixed effects: age stratification (infant, children, adult and overall), population density, and whether all SD patients in a study underwent autopsies (yes or no) [19]. We obtained population density data for each country in 2020 from the WorldPop database (WorldPop, University of Southampton, Southampton, UK), with a spatial resolution of 1 × 1 km and treated them as a continuous variable.

With respect to the selection of studies, we intended to choose those with the latest data on the relevant variables and then select studies that provided the most comprehensive data on those variables. Specifically, studies with identical study areas, study periods, study populations, and age groups were included only once to avoid duplication of data sources. Geographic clustering of countries and regions was accounted for in our model to estimate the incidence of SD in countries with missing data. This suggests that the estimates of incidence depend on evidence borrowed from higher levels in this model. Briefly, when specific national data were unavailable, we utilised regional estimates; when corresponding regional data were unavailable, we resorted to super-regional estimates. Notably, both regional and super-regional incidence rates were estimated from national-level studies, indicating that no regional or super-regional studies were conducted. It should be noted that super-regional estimates differ from regional estimates within a super-region that encompasses only one region, as the super-regional estimates serve as the initial values for deriving regional estimates.

The statistical model employed to estimate the incidence of SD utilised Bayesian inference and sampled from the posterior distributions of the parameters using the Hamiltonian Markov Chain Monte Carlo framework [21]. The model was run with four chains, each undergoing 2000 iterations to ensure sampling robustness. We generated posterior predictions for each country-age group combination, uniformity of autopsy across study populations, and population density, providing incidence estimates expressed as proportions with 95% CIs [19]. To determine the national burden of individuals affected by SD, we multiplied the country-specific incidence estimates by the corresponding population sizes, using the demographic framework published by the United Nations in 2022. The adequacy of the model fit was rigorously appraised through an assessment of diagnostics pertinent to the effective sample size, autocorrelation, and trace plots, ensuring the reliability of our inferential process (Figures S4 and S5 in the **Online Supplementary Document**).

We performed all spatial analyses using ArcGIS, version 10.8 (Esri Inc, Redlands, California, USA). We conducted all statistical analyses using *R*, version 4.2.0 (R Core Team, Vienna, Austria) and the package ‘RStanArm’ (Stan Development Team, New York, New York, USA). Statistical tests were two-sided, with statistical significance defined as a *P*-value <0.05.

## RESULTS

**Figure 1:**
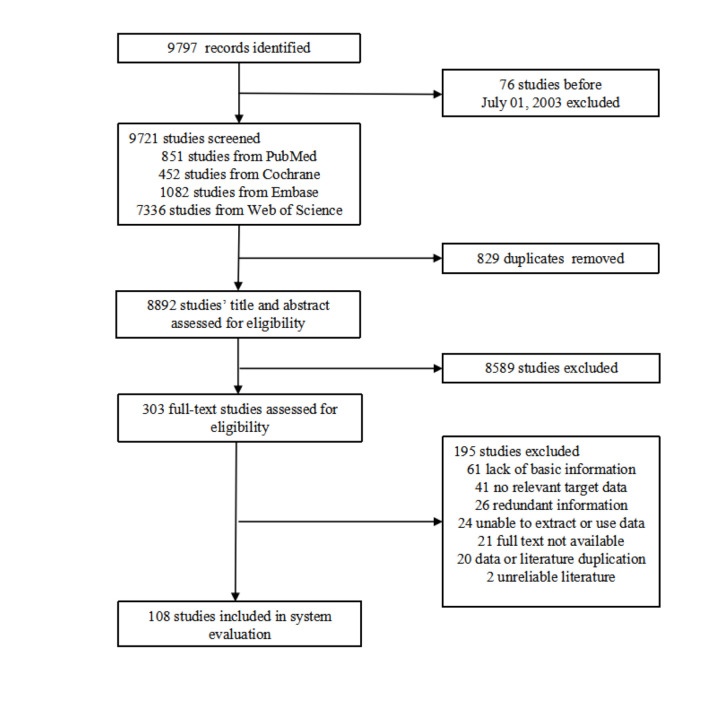
PRISMA diaphragm of study selection.

We identified 9797 potential studies after searching the four main databases regarding SD. Among these, we excluded 76 because of date-related reasons, and 829 were duplicate studies. According to the eligibility criteria, we further excluded 8589 studies. Subsequently, 195 studies failed to meet the inclusion and exclusion criteria for the following reasons, including: a lack of basic information (n = 61), presenting all irrelevant data (n = 41), redundant information (n = 26), data unable to extract or use (n = 24), unavailable full text (n = 21), duplicated date or literature (n = 20), and unreliable literature (n = 2). Finally, we included 108 studies in evaluation (**Figure 1**).

We conducted a comprehensive analysis of 108 English-language literature sources published between 1 July 2003 and 30 June 2023. The majority of the included studies were categorised as having low to moderate risk of bias (n/N = 98/108) (Table S8 in the **Online Supplementary Document**. The pooled data from these studies showed a total of approximately 201 549 cases of SD, with 61 341 cases attributed to SCD (Figure S1 and Table S10 in the **Online Supplementary Document**). The geographical distribution of these studies was widespread, encompassing 30 countries across six continents including Europe with 15 countries, Asia with seven, Africa with three, North America with two, Oceania with two, and South America with one, respectively. Notably, the majority of the studies originated from North America (n = 29), followed by Australia (n = 9), UK (n = 8), China (n = 7), and Spain (n = 6) (Table S4 in the **Online Supplementary Document**). The temporal scope of the data extended from 1948 to 2022, with the majority of the studies conducted between the years 2000 and 2009 and carried out in high-income regions, which constituted 82.40% of the total, contrasting with a solitary study from a low-income region, Cameroon. In terms of study duration, more than half of the studies were of shorter duration, ranging from 0–14 years, with specific breakdowns as follows: 0–4 years (n = 26; 24.07%), 5–9 years (n = 24; 22.22%), and 10–14 years (n = 23; 21.30%). Most of the studies were designed as cohort studies. The majority of the studies reviewed were published during the period from 2013 to 2017 (n = 46; 42.59%), succeeded by research conducted between 2018 and 2023 (n = 33; 30.56%) (Table S5 in the **Online Supplementary Document**).

Among the cases of SD reviewed, demographic features showed significant heterogeneity. Specifically, the proportion of SD was distinctly higher among whites (54.83%) and married individuals (50.70%). Moreover, in accordance with China’s 2015 occupational classification, SD cases were disproportionately concentrated among professional technicians (20.52%) and manufacturing personnel (25.84%) (**Table 1**).

**Table 1:** Summary of demographic characteristics of sudden death (SD) included in the systematic assessment*

Demographic characteristics	n (%)
Sex (n = 103 362)	
*Male*	56 191 (54.36)
*Female*	47 171 (45.64)
Age group in years (n = 108)	
*0–3*	23 (21.30)
*4–14*	25 (23.15)
*15–49*	47 (43.52)
*50–59*	4 (3.70)
*60–69*	5 (4.63)
*≥70*	1 (0.93)
*Not sure*	3 (2.78)
Race (n = 49 284)	
*White*	27 021 (54.83)
*Yellow*	9703 (19.69)
*Black*	7465 (15.15)
*Others*	5095 (10.34)
BMI in kg/m^2^ (n = 2366)	
*>25.0 (overweight)*	1685 (71.22)
*>30.0 (obesity)*	681 (28.78)
Marital status (n = 71)	
*Married*	36 (50.70)
*Single*	17 (23.94)
*Others*	18 (25.35)
Occupation category (n = 2763)†	
*First*	126 (4.56)
*Second*	567 (20.52)
*Third*	354 (12.81)
*Fourth*	395 (14.30)
*Fifth*	0
*Sixth*	714 (25.84)
*Seventh*	200 (7.24)
*Eighth*	407 (14.73)
State before death (n = 8550)	
*Sleeping*	2224 (26.01)
*Working*	263 (3.08)
*Exercise*	1981 (23.17)
*Rage*	528 (6.18)
*Daily activities*	675 (7.89)
*Relax*	708 (8.28)
*Drinking*	43 (0.50)
*Medical treatment*	91 (1.06)
*Driving*	45 (0.53)
*Others*	1992 (23.30)
Season (n = 11 915)	
*Spring*	2666 (22.38)
*Summer*	2304 (19.34)
*Autumn*	2869 (24.08)
*Winter*	4076 (34.21)
Family history of SD (n = 667)	
*Yes*	551 (82.61)
*No*	24 (3.60)
*Not sure*	92 (13.79)

BMI – body mass index, SD – sudden death

*The total number of SD cases included in the systematic assessment was 201 549.

†Occupational classification standard: The first category encompasses public service and administrative personnel. The second category comprises professional and technical personnel. The third category pertains to clerical and administrative support workers, who provide organisational support. The fourth category involves service workers. The fifth category consists of agricultural, forestry, and fishing workers. The sixth category includes manufacturing and production workers. The seventh category is military personnel. The eighth category comprises other workers who do not fit neatly into the aforementioned classifications.

**Figure 2:**
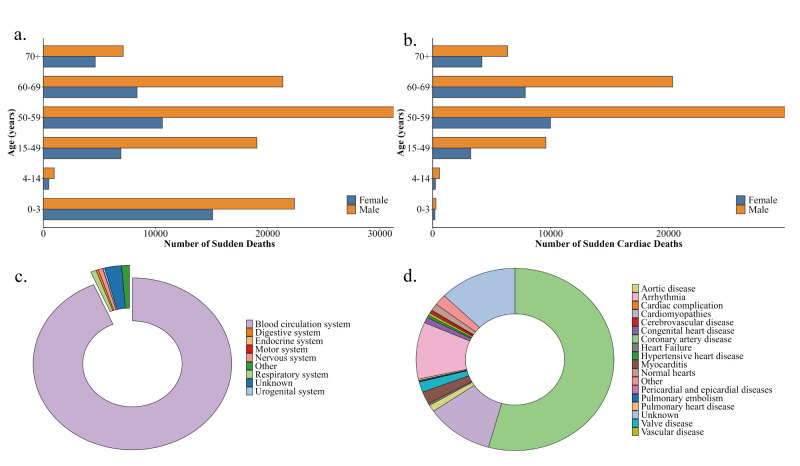
Distribution and causes of sudden death (SD) stratified by age, sex, disease category. **Panel A.** SD quantitative distribution by age and gender. **Panel B.** Sudden cardiac deaths (SCD) quantitative distribution by age and gender. **Panel C.** Causes of SD stratified by organ systems. **Panel D.** Causes of SCD stratified by specific disease.

Cardiovascular system diseases account for the majority of SD cases, with a higher proportion of 93.6%. Additionally, respiratory system diseases also account for a substantial proportion of SD cases. The main contributor of SCD was coronary artery disease, which accounted for more than half of the cases. Subsequent causes included cardiomyopathies and arrhythmias, with comparable proportions. A considerable proportion of individuals experience SD during sleep or exercise. Discrepancies in age and gender were also apparent, with individuals aged 50–59 years and males representing a larger proportion of cases, contrasting with the relatively smaller proportion observed in the 4–14 years age group. Diabetes, hypertension, hyperlipidaemia, and heart disease were important risk factors in the majority of SD cases, with hypertension being particularly prevalent, affecting nearly one-third of the cases. Concurrently, the results showed that obesity and smoking were also major factors contributing to SD (**Figure 2**; Figure S5 in the **Online Supplementary Document**).

Based on the extracted incidence rate data of SD in 21 regions worldwide, we stratified the SD rates by time period based on the midpoint of each study cycle. The results showed substantial fluctuations in global SD incidence. The incidence of SD peaked at 233.8 cases (95% CI = 210.74, 258.04) per 100 000 person-years during 1971–1980, then sharply declined to 4.83 (95% CI = 4.61, 5.04) per 100 000 person-years during 1981–1990. From 1991 to 2010, global SD incidence rates stabilised at 20.57 (1991–2000) and 18.39 (2001–2010) before dropping to the lowest recorded level in 2011–2020 with 7.39 (95% CI = 2.19, 15.63) per 100 000 person-years (Figure S3 and Table S6 in the **Online Supplementary Document**). According to Havmoeller [22], the incidence rate among the USA population was 58.00 cases (95% CI = 54.75, 61.44) per 100 000 person-years from 2002 to 2005. Two studies investigated incidence rate of SD in children population aged between two and 17 years reported at 0.3 cases (95% CI = 0.20,0.50) per 100 000 person-years from 1986 to 2011 [23], while the incidence rate from 2007 to 2013 exhibited a rise, reaching 0.99 cases (95% CI = 0.78, 1.26) per 100 000 person-years [24]. Conversely, the incidence of SD noted 13.00 cases (95% CI = 10.90, 15.50) per 100 000 person-years from 1977 to 2001 in adult [25], escalating to 15.44 cases (95% CI = 13.86, 17.21) per 100 000 person-years from 2011 to 2013 [26]. In addition, a compelling study undertaken in Denmark highlighted that the incidence among children from 2000 to 2006 was reported at 1.47 cases (95% CI = 1.21, 1.77) per 100 000 person-years [27], (**Table 2**) whereas the total population incidence reached 8.60 cases (95% CI = 8.00, 9.20) per 100 000 person-years from 2000 to 2009 [36], thereby indicating a rising trend in overall incidence rates within the general population, with adult incidence rates surpassing those of their younger counterparts. Moreover, it is particularly that Rao’s research indicated the incidence rate of total population in India soared to 761.31 cases (95% CI = 654.28, 885.3) per 100 000 person-years in 2010 [37], while concurrently, Germany was reported 81.00 cases (95% CI = 76.54, 85.72) per 100 000 person-years from 2002 to 2009 [40], which further underscores the alarming incidence of SD across various global contexts (**Table 2**; Table S9 in the **Online Supplementary Document**).

**Table 2:** Comparative incidence rates and characteristics of sudden death (SD) across children, adults, and overall populations in the included studies

Study	Study period and country	Age of the population in years	SD	Incidence rate per 100 000 person-years (95% CI)		
				**Total**	**Male**	**Female**
Children						
*Maron et al., 2012 [* 23 *]*	1986–2011, USA	12–18	17	0.30 (0.20, 0.50)*	0.50 (0.30, 0.90)*	0
*Winkel et al., 2013 [* 27 *]*	2000–2006, Denmark	1–18	87	1.47 (1.21, 1.77)*†	NA	NA
*Harmon et al., 2016 [* 24 *]*	2007–2013, USA	14–18	69	0.99 (0.78, 1.26)*†	1.45 (1.12, 1.89)*†	0.32 (0.15, 0.62)*†
Adult						
*Eckart et al., 2004 [* 25 *]*	1977–2001, USA	NA	126	13.00 (10.90, 15.50)*	13.30 (11.00, 16.00)*	11.50 (7.00, 18.90)*
*Niemeijer et al., 2015 [* 28 *]*	1990–2010, Netherlands	≥45	583	422.64 (389.41, 458.65)*†	513.43 (455.87, 578.1)*†	363.61 (324.47, 407.39)*†
*Kim et al., 2016 [* 26 *]*	2011–2013, USA	≥18	336	15.44 (13.86, 17.21)*†	21 (18.43, 23.92)*†	9.66 (7.92, 11.77)*†
*Feng et al., 2016 [* 29 *]*	1997–2010, Australia	35–84	8077	58.45 (57.19, 59.74)*†	82.67 (80.54, 84.85)*†	34.44 (33.08, 35.86)*†
*Bonny et al., 2017 [* 30 *]*	2013, Cameroon	≥18	135	1.63 (1.37, 1.92)*	1.87 (1.57, 2.21)*	0.30 (0.51, 1.00)*
*Shuvy et al., 2019 [* 31 *]*	2003–2014, Canada	35–74	36 334	49.11 (48.61, 49.62)*†	75.27 (74.38, 76.17)*†	23.98 (23.49, 24.48)*†
*Park et al., 2020 [* 32 *]*	2002–2015, USA	≥20	197	39.86 (37.77, 42.06)*†	47.54 (44.39, 50.92)*†	31.59 (28.94, 34.48)*†
Overall						
*Tabib et al., 2003 [* 33 *]*	1980–1999, France	1–65	200	4.82 (4.61, 5.05)*†	NA	NA
*Moore et al., 2005 [* 34 *]*	2003–2004, UK	NA	300	19.88 (17.73, 22.30)*†	NA	NA
*Byrne et al., 2008 [* 35 *]*	2005, Ireland	0–80	212	51.17 (44.62, 58.67)*†	76.42 (65.24, 89.47)*†	25.38 (19.14, 33.55)*†
*Fragkouli et al., 2010 [* 36 *]*	1998–2008, Greece	1–80	688	32.22 (30.47, 34.07)*†	NA	NA
*Rao et al., 2010 [* 37 *]*	3010, India	≤90	173	761.31 (654.28, 885.3)*†	NA	NA
*Havmoeller et al., 2012 [* 22 *]*	2002–2005, USA	0–85	1175	58.00 (54.75, 61.44)*†	70.00 (65.00, 75.37)*†	45.00 (41.03, 49.35)*†
*Pilmer et al., 2013 [* 38 *]*	2005–2009, Canada	1–19	116	0.78 (0.64, 0.93)*	1.01 (0.8, 1.27)*†	0.54 (0.39, 0.75)*†
*Hofer et al., 2014 [* 39 *]*	2000–2007, Switzerland	5–39	40	1.71*‡	2.73*‡	0.69*‡
*Martens et al., 2014 [* 40 *]*	2002–2009, Germany	0–90	1212	81.00 (76.54, 85.72)*†	55 (51.37, 58.89)*†	25 (22.56, 27.70)
*Braggion-Santos et al., 2015 [* 41 *]*	2006–2010, Brazil	10–80	899	29.97 (28.05, 32.01)*†	NA	NA
*Zheng et al., 2015 [* 8 *]*	2007–2013, China	0–111	879	1.26 (1.18, 1.34)*	14.83*	1.26*
*Zhao et al., 2016 [* 42 *]*	1997–2012, Seychelles	0–97	484	32.88 (30.05, 35.98)*†	NA	NA
*Bagnall et al., 2016 [* 43 *]*	2010–2012, Australia	1–35	490	1.30 (1.20, 1.40)*†	1.80 (1.62, 2.00)*†	0.70 (0.59, 0.83)*†
*Risgaard et al., 2016 [* 44 *]*	2000–2009, Denmark	1–49	1575	8.60 (8.00, 9.20)*	NA	NA
*Ifteni et al., 2017 [* 45 *]*	2001–2015, Romania	0–89	1085	18.08 (17.03, 19.20)*†	NA	NA
*Wisten et al., 2016 [* 46 *]*	2000–2010, Sweden	1–35	552	1.29 (1.18, 1.40)*†	NA	NA
*Belhaj et al., 2023 [* 47 *]*	2010–2019, Tunisia	NA	2385	2.15 (2.06, 2.24)*†	NA	NA
*Frontera et al., 2022 [* 48 *]*	2010–2015, Italy	1–35	301	0.24 (0.13, 0.45)*	0.40*‡	0.10*‡
*Rücklová et al., 2022 [* 49 *]*	2014–2019, Czech Republic	1–40	245	2.44 (2.15, 2.77)*†	3.20*‡	0.80*‡
*Işın et al., 2021 [* 50 *]*	2011–2019, Turkey	10–59	118	0.41*‡	NA	NA
*Ripoll-Vera et al., 2021 [* 51 *]*	2015–2019, Spain	0–50	123	5.8*‡	10.10*‡	1.60*

CI – confidence interval, NA – not available, SD – sudden death

*Value reported from the study.

†CI estimated and added by the authors as it was not present in the original study.

‡It was not possible to calculate the confidence interval due to a lack of raw data.

In 108 included studies, we identified a subset of 10 studies specifically addressing sudden infant death syndrome (SIDS), which accounted for 69 096 cases. The majority of these studies were conducted in Europe and the Americas, highlighting the regional focus within the research landscape. It was noteworthy that the proportion of SIDS appears to be evenly distributed among males and females, with the majority of cases occurring within zero to one years (**Table 3**). The recorded incidence rates of SIDS vary significantly across regions. Chang *et al.* reported the population incidence was recorded 71.22 cases (95% CI = 69.48, 73.00) per 100 000 person-years from 1989 to 2008 in the USA [52], which was broadly comparable with the reports of Hakeem *et al.* (64.45 cases per 100 000 person-years) [56] and Bartick *et al.* (91.31 cases per 100 000 person-years) [60]. However, this was in stark contrast to the incidence of SIDS reported by Malloy *et al.* (43.56 cases per 100 000 person-years) [54] and Drake *et al.* (106.18 cases per 100 000 person-years) [58]. The incidence of SIDS varied over time within the same country. Unexpectedly, the incidence of SIDS in France from 2000 to 2017 was 12.75 cases (95% CI = 10.69, 15.19) per 100 000 person-years, as reported by Tuchtan *et al.* [57]. Based on incidence data extracted from the literature, the difference in SIDS incidence between male and female infants in the United States is not statistically significant (Table S12 in the **Online Supplementary Document**).

**Table 3:** The characteristics and epidemiological data of sudden infant death syndrome (SIDS) in the studies included

Study	Study period	Continent	Country	Incidence rate (per 100 000 person-years)	Age in years	Male, n (%)	Race, n				Total cases, n*
							**White**	**Yellow**	**Black**	**Other**	
Chang *et al.*, 2008 [52]	1998–2004	North America	USA	71.22	0–1	3872 (61.43)	4626	454	1222	0	6303
Liebrechts *et al.*, 2013 [53]	1984–2005	Europe	Netherlands	18.00	0–1	110 (58.82)	NR	NR	NR	NR	187
Malloy *et al.*, 2013 [54]	2005–2007	North America	USA	43.56	2–3.5	3111 (59.79)	2856	932	1415	0	5203
Evans *et al.*, 2013 [55]	2000–2010	Oceania	Australia	NR	0–1	138 (61.06)	NR	NR	NR	NR	226
Hakeem *et al.*, 2015 [56]	1995–2004	North America	USA	64.45	0–1	14 300 (59.33)	NR	NR	NR	NR	24 101
Tuchtan *et al.*, 2019 [57]	2000–2017	Europe	France	12.75	0–2	80 (61.54)	NR	NR	NR	NR	130
Drake *et al.*, 2019 [58]	2004–2013	North America	USA	106.18	0–1	421 (57.51)	403	19	310	0	732
Mitchell *et al.*, 2023 [59]	2012–2018	Oceania	New Zealand	63.87	0–1	137 (51.31)	0	267	0	0	267
Bartick *et al.*, 2022 [60]	2015–2018	North America	USA	91.31	0–1	NR	7656	416	3870	0	11 942
Anderson *et al.*, 2022 [61]	2006–2015	North America	USA	NR	0–1	NR	7134	NR	NR	5010	20 005

NR – not reported

*Number of SIDS cases included in the study.

**Figure 3:**
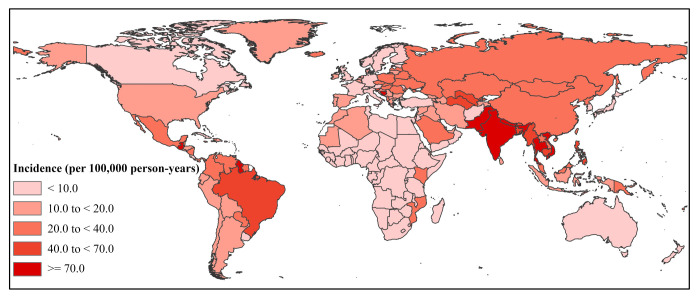
Geographical distribution of estimated incidence of sudden death (SD) by country. The estimated national incidence of SD was estimated using Bayesian hierarchical linear mixed models. Details about countries with observed or extrapolated data are given in the appendix.

Based on our models, the estimates of SD incidence revealed substantial geographic and temporal heterogeneity across general and age-stratified populations. Among seven super-regions, South Asia had the highest incidence rate at 4.01 cases (95% CI = 1.40, 7.51) per 100 000 person-years on a log-transformed basis, followed by South East Asia, east Asia, and Oceania for 3.38 (95% CI = 1.69, 5.29) and Latin America and Caribbean for 3.36 (95% CI = 1.16, 6.39) per 100 000, respectively. Sub-Saharan Africa demonstrated the lowest incidence for 1.79 cases (95% CI = –1.83, 3.98) per 100 000 person-years (**Figure 3**; Table S11 in the **Online Supplementary Document**). For different regions, Asia South had the highest incidence rate at 4.51 cases (95% CI = 1.85, 7.74) per 100 000, and a high-incidence cluster had formed in the Latin America region and the Caribbean region, with a rate of 3.28 to 3.64 cases per 100 000. East Asia (3.47) and Southeast Asia (3.62) are close to the level in Andean Latin America (3.44). The results of the age-stratified analysis showed that children had a significantly lower incidence than adults and infants, with South Asia consistently showing peak rates across age strata. Infants were estimated the highest incidence rate at 5.74 cases (95%CI = 3.28, 9.85) per 100 000 person-years, following adults with 5.72 cases (95%CI = 2.72, 9.39) per 100 000 person-years and children demonstrated the lowest incidence for 2.39 cases (95% CI = –0.95, 6.22) per 100 000. Besides, Latin America and the Caribbean constitute a persistently high-burden cluster (Figures S6–8 in the **Online Supplementary Document**). Nationally, Guyana was estimated to have the highest incidence of 5.06 cases (95% CI = 2.78, 8.20) per 100 000, followed by India for 4.89 cases (95% CI = 2.08, 7.76) per 100 000 person-years and Saint Vincent and the Grenadines for 4.76 cases (95% CI = 2.46, 7.74) per 100 000 person-years (Table S11 in the **Online Supplementary Document**). The results from the generalised linear mixed models indicate that the global overall decline has significant super-regional heterogeneity. High-income countries were estimated to have the largest declines despite the greatest dispersion, and Sub-Saharan Africa maintained a stable high rate. Southeast Asia, East Asia and Oceania demonstrated nonlinear progress since 2005. Latin America and the Caribbean continued to grow, with the Andes region being the most notable (Figure S3 in the **Online Supplementary Document**).

## DISCUSSION

We provided a comprehensive description of the global incidence and disease burden of SD, quantifying substantial global heterogeneity among SD cases across varying demographic characteristics. South Asia exhibited the highest incidence of SD, which could be attributed to racial differences, a lack of overall medical resources and awareness, and underlying disease factors [62]. Consistent with research on sudden cardiac arrest, Blacks are more prone to congestive heart failure and left ventricular hypertrophy, and they also have a longer corrected QT interval [62]. In India, low out-of-hospital cardiac arrest survival rates are linked to deficient cardiac arrest recognition [63–65], low bystander cardiopulmonary resuscitation (CPR) rates [66,67], legal and social concerns regarding CPR provision [68], and limited emergency medical services [69].

Sub-Saharan Africa (especially western regions) showed the lowest SD incidence, aligning with advances in reducing HIV-related CVD through improved testing/diagnosis/treatment [70] and younger demographics (64.2% aged <25years) [69]. Nevertheless, inadequate reporting partially underestimates incidence [71].

Country-level variations were significant, with Guatemala in Latin America, in particular, exhibiting the highest incidence rate of SD due to Chagas disease [72] and poor adherence to implantable cardioverter defibrillator [73]. Comparatively, countries in Southern Latin America, such as Argentina, Chile, and Uruguay, reported considerably lower incidence rates of SD (Figure S7 in the **Online Supplementary Document**). This disparity aligns with findings associated with higher total health expenditure and a more developed healthcare infrastructure [74].

Demographically, most SCD cases occur in adults [74], with incidence increasing with age [73]. Our study indicates that SIDS also constitutes a notable proportion of such fatalities in the infant population. We identified 50–59 years as high-risk due to excessive stress. Females show lower SD incidence with protective factors involving oestrogen, pregnancy, and cellular electrophysiology [75,76]. White ethnicity and higher socioeconomic status are associated with reduced incidence [77], and the discrepancy in the incidence of SD among whites may be explained by the population sizes of the different races included in our review.

Cardiac causes predominate, with coronary artery disease being responsible for more than half of deaths related to the circulatory system (**Figure 2**). Comprehensive risk reduction requires multi-factorial interventions; established predictors include hypertension, diabetes, dyslipidaemia, smoking, and obesity [3,4,10]. Genetic factors such as pathogenic/likely pathogenic variants [78] and Myosin Heavy Chain seven mutations [79] support existing evidence. Moreover, women are underrepresented in cardiovascular trials and face barriers to medical access [10], necessitating gender-specific prevention strategies [80]. The true SD incidence remains controversial due to data source and collection biases [81].

Global SD incidence peaked at 233.8 cases per 100 000 person-years during 1971–1980, then declined. However, it is worthy of particular attention that only one piece of literature was included for both periods of 1971–1980, and we need to interpret this analysis. Our research results showed that the global incidence rate was generally declining. This reduction aligns with synergistic improvements in: medical technology (>80% coverage of percutaneous coronary intervention/coronary artery bypass grafting/automated external defibrillator enhancing emergency care), public health policies (Australia’s tobacco taxation increasing hypertension control from 32% to 54%), and localised interventions (China’s chest pain networks reducing ST-segment elevation myocardial infarction mortality by 28%) [11]. The mortality rate of SD for the zero to three year age group deviated from the overall age-incidence trend, with an overall increase mainly attributed to SIDS, which remains the leading cause of death between one to 12 months despite a >50% decline over 20 years [82]. Cultural practices correlate with SIDS incidence [82]. Given that three mechanisms of SIDS have been widely identified-preterm or exposed to maternal smoking, exogenous stressor and immature cardio-respiratory control-recommendations for the prevention of SIDS have been proposed [83]. These included sleeping in a safe environment, breastfeeding on demand, and abstaining from tobacco, alcohol, and illicit drugs during pregnancy and postpartum by parents [84].

Lifestyle and environmental factors influence SD occurrence – manufacturing and production workers [85] and blue-collar occupations show a higher risk, potentially due to physical and mental stress, which may be related to long-term exposure [86]. Seasonal variations (higher incidence in autumn and winter) may be related to cold-induced physiological stress or increased exposure during these seasons [87].

Our study has several strengths. Principally, this study represented the first systematic assessment and review of relevant literature on SD cases spanning 60 years globally, contributing valuable academic insights to the medical field. Additionally, our research was innovative in employing a comprehensive and systematic methodology to investigate and analyse a multi-centre, large-sample database. Elucidating epidemiological characteristics and occurrence patterns guides mitigating disease burden and reducing the incidence of SD.

There are also some limitations to note. First, due to variations in the sample sizes of the studies included across the seven continents, with a predominant focus on Europe, Asia, and South America, there may be differences in the accuracy of estimates of global incidence rates. One possible reason for this bias is that our study only includes English-language literature. Therefore, among the 108 included studies, 61.11% focused on SCD, *vs.* 20.37% on other systemic SD. Although the majority of included primary studies were judged to have a low to moderate risk of bias, their inherent limitations may still lead to overestimation or underestimation of the results. Finally, the inclusion of studies with small sample sizes or retrospective designs may negatively affect the validity and reliability of the evidence.

## CONCLUSIONS

Overall, the incidence of SD has been systematically assessed, revealing notable national and regional variance, accompanied by discrepancies in demographic characteristics, primarily within the domains of age, gender, occupation, race, and income level, with particular emphasis on SIDS. Throughout this 60-year review, the circulatory system has predominantly been implicated in the origin of SD. Taking the existing limitations into consideration, a more valid and reliable assessment is encouraged for future studies.

## Additional material


Online Supplementary Document
