# Supplementary material for: Global, regional, and national characteristics and incidence of sudden death from 1948 to 2022: a systematic review and modelling study
Source: J Glob Health. 2026 Jul 31;16:04222. doi: 10.7189/jogh.16.04222 (PMC13425371; doi:10.7189/jogh.16.04222)
Supplement: Online Supplementary Document [file jogh-16-04222-s001.pdf]

Supplement to: Pan S, Huang X, Wu X, Chen B, Su X, Fang L, Dai T, Guo P. Global, regional, and national characteristics and incidence of sudden death from 1948 to 2022: a systematic review and modelling study. J Glob Health. 2026;16:04222.

Contents

Figure S1. Included number of sudden death cases (a) and sudden cardiac death cases (b) by location. The global map visually represents the distribution of included cases across various regions. The map utilizes a color-coded system, where different shades of color distinguish between locations, and the intensity of the coloration indicates the relative number of cases included in the study. This visual approach provides an immediate understanding of the geographic distribution of sudden deaths and sudden cardiac deaths, highlighting areas with higher concentrations of reported cases. The use of color gradation effectively conveys the prevalence of these sudden death events on a global scale, emphasizing the need for targeted public health initiatives and further research in the regions most affected.

Figure S2. Risk factors of sudden death. The pie chart provides a graphical summary of the distribution of various risk factors associated with the risk of sudden death. Each segment of the pie, differentiated by color, corresponds to a distinct risk factor, allowing for an immediate and comparative assessment of their prevalence. The area of each segment is proportional to the percentage of the population affected by the associated risk factor. A larger segment indicates a higher proportion of the population exposed to that particular risk factor, while a smaller segment suggests a lower influence.

Figure S3. Temporal trends in sudden death incidence. Temporal trends for 21 regions based on geography and income hierarchy. The size of the bubble represents the number of person-years included in each study. The position of the bubble is determined by the midpoint of the study period and the incidence rate of the study. The fitted curves derived from generalized linear model show the trends in incidence over time.

Figure S4. Trace plots and variance for the fixed and random effects based on the Bayesian hierarchical linear mixed model. The plot depicts the parameter traces, which are meticulously recorded over successive iterations within the framework of a Bayesian hierarchical linear mixed model. The parameters that correspond to fixed effects are distinctly represented in blue, thereby highlighting the interrelationships in prevalence rates across various age demographics: infants, children, adults, and the encompassing age groups. In contrast, the parameters for random effects are rendered in orange, effectively conveying the variability inherent to different geographical strata, namely country, region, and super-region, within the categorical spectrum. It is observed that the variance is more pronounced at the regional level in comparison to the national level, with an even greater magnitude at the super-regional level. This pattern of variance is reflective of the heterogeneity observed in the input data for the respective parameters, thereby indicating a consistent trend across the board. Incidence rates is higher in the general (all age groups) population and the adult population than in infants.

Figure S5. Posterior predictive check plot comparing observed and simulated values from the Bayesian hierarchical model. In the plot, y denotes the observed value, and y_{rep} denotes the fitted value.

Figure S6. Estimated incidence of sudden death for infants in 21 regions. The

error bars indicate 95% credible intervals (95% CI). The different color represents the different super region, respectively.

Figure S7. Estimated incidence of sudden death for children in 21 regions. The error bars indicate 95% credible intervals (95% CI). The different color represents the different super region, respectively.

Figure S8. Estimated incidence of sudden death for adults in 21 regions. The error bars indicate 95% credible intervals (95% CI). The different color represents the different super region, respectively.

Table S1. Search strategies for the selection of literature sources in this systematic review.

Table S2. Studies excluded during screening.

Table S3. Studies excluded post-full text screening.

Table S4. Basic information of the studies included by location.

Table S5. Summary characteristics of the studies included in the systematic assessment.

Table S6. Pooled incidence of sudden death by time period globally. The time periods are categorized based on the midpoint of each study period.

Table S7. Integrated classification of countries, regions and super-regions in the analyzed areas.

Table S8. Characteristics of studies reporting on incidence of sudden death.

Table S9. Details information on the incidence of sudden death in children, adults, and the overall population of the studies.

Table S10. Studies reporting on the incidence of sudden death in overall population.

Table S11. Estimated incidence of sudden death (per 100,000 person-years) by country, region, and super-region.

Table S12. Incidence trends of SIDS in included studies.

Table S13. The articles included in the systematic review.

Figure S1. Included number of sudden death cases (a) and sudden cardiac death cases (b) by location. The global map visually represents the distribution of included cases across various regions. The map utilizes a color-coded system, where different shades of color distinguish between locations, and the intensity of the coloration indicates the relative number of cases included in the study. This visual approach provides an immediate understanding of the geographic distribution of sudden deaths and sudden cardiac deaths, highlighting areas with higher concentrations of reported cases. The use of color gradation effectively conveys the prevalence of these sudden death events on a global scale, emphasizing the need for targeted public health initiatives and further research in the regions most affected.

a.

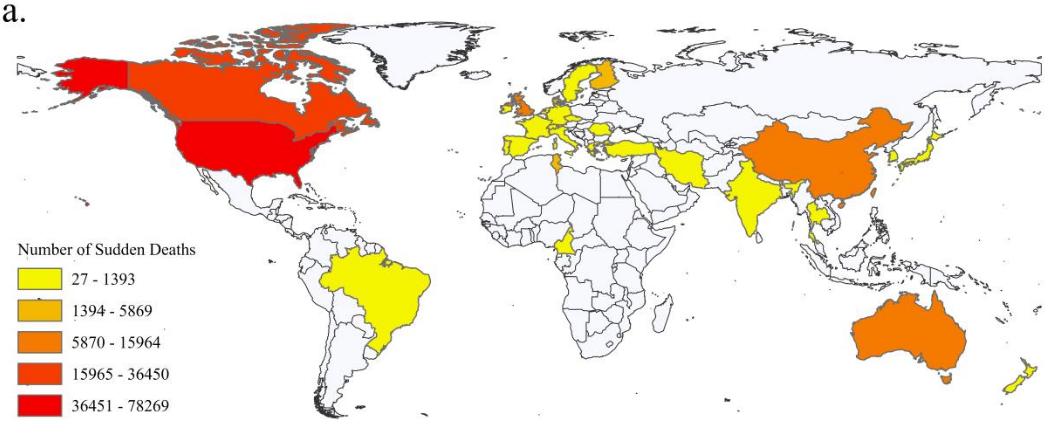

b.

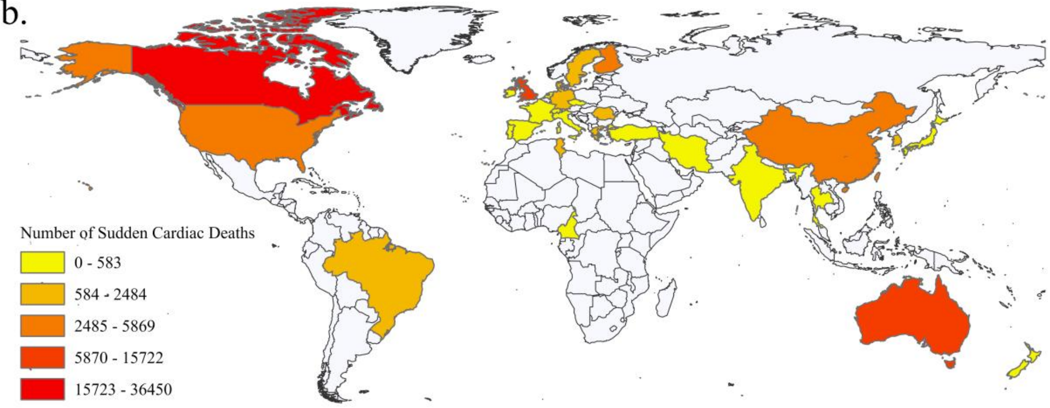

Figure S2. Risk factors of sudden death. The pie chart provides a graphical summary of the distribution of various risk factors associated with the risk of sudden death. Each segment of the pie, differentiated by color, corresponds to a distinct risk factor, allowing for an immediate and comparative assessment of their prevalence. The area of each segment is proportional to the percentage of the population affected by the associated risk factor. A larger segment indicates a higher proportion of the population exposed to that particular risk factor, while a smaller segment suggests a lower influence.

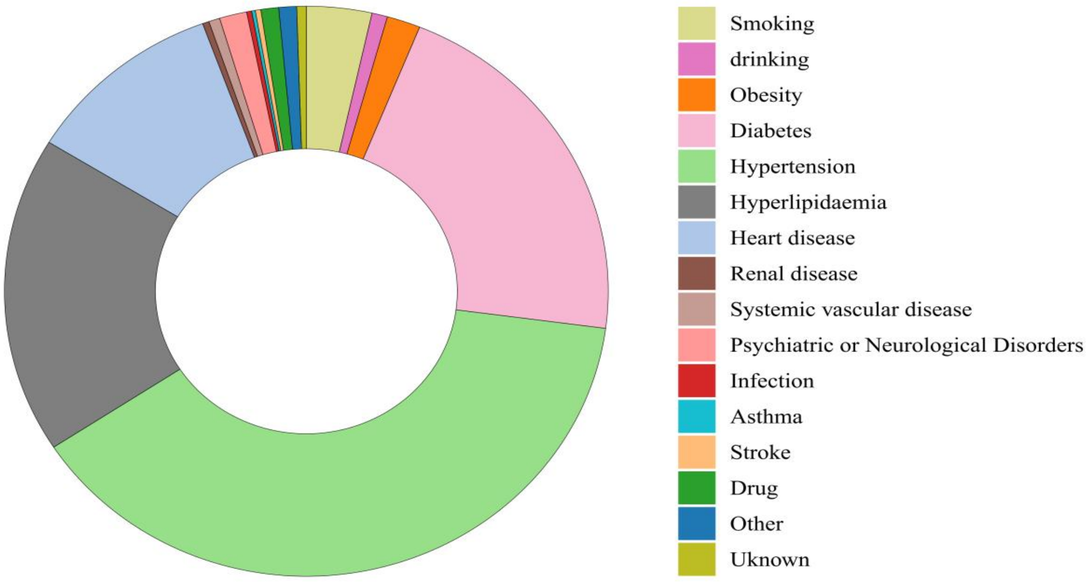

Figure S3. Temporal trends in sudden death incidence. Temporal trends for 21 regions based on geography and income hierarchy. The size of the bubble represents the number of person-years included in each study. The position of the bubble is determined by the midpoint of the study period and the incidence rate of the study. The fitted curves derived from generalized linear model show the trends in incidence over time.

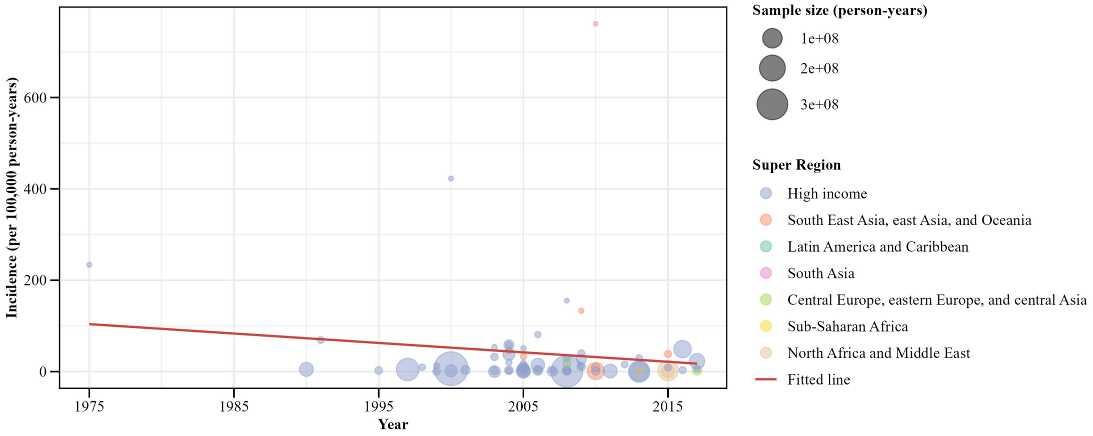

Figure S4. Trace plots and variance for the fixed and random effects based on the Bayesian hierarchical linear mixed model. The plot depicts the parameter traces, which are meticulously recorded over successive iterations within the framework of a Bayesian hierarchical linear mixed model. The parameters that correspond to fixed effects are distinctly represented in blue, thereby highlighting the interrelationships in prevalence rates across various age demographics: infants, children, adults, and the encompassing age groups. In contrast, the parameters for random effects are rendered in orange, effectively conveying the variability inherent to different geographical strata, namely country, region, and super-region, within the categorical spectrum. It is observed that the variance is more pronounced at the regional level in comparison to the national level, with an even greater magnitude at the super-regional level. This pattern of variance is reflective of the heterogeneity observed in the input data for the respective parameters, thereby indicating a consistent trend across the board. Incidence rates is higher in the general (all age groups) population and the adult population than in infants.

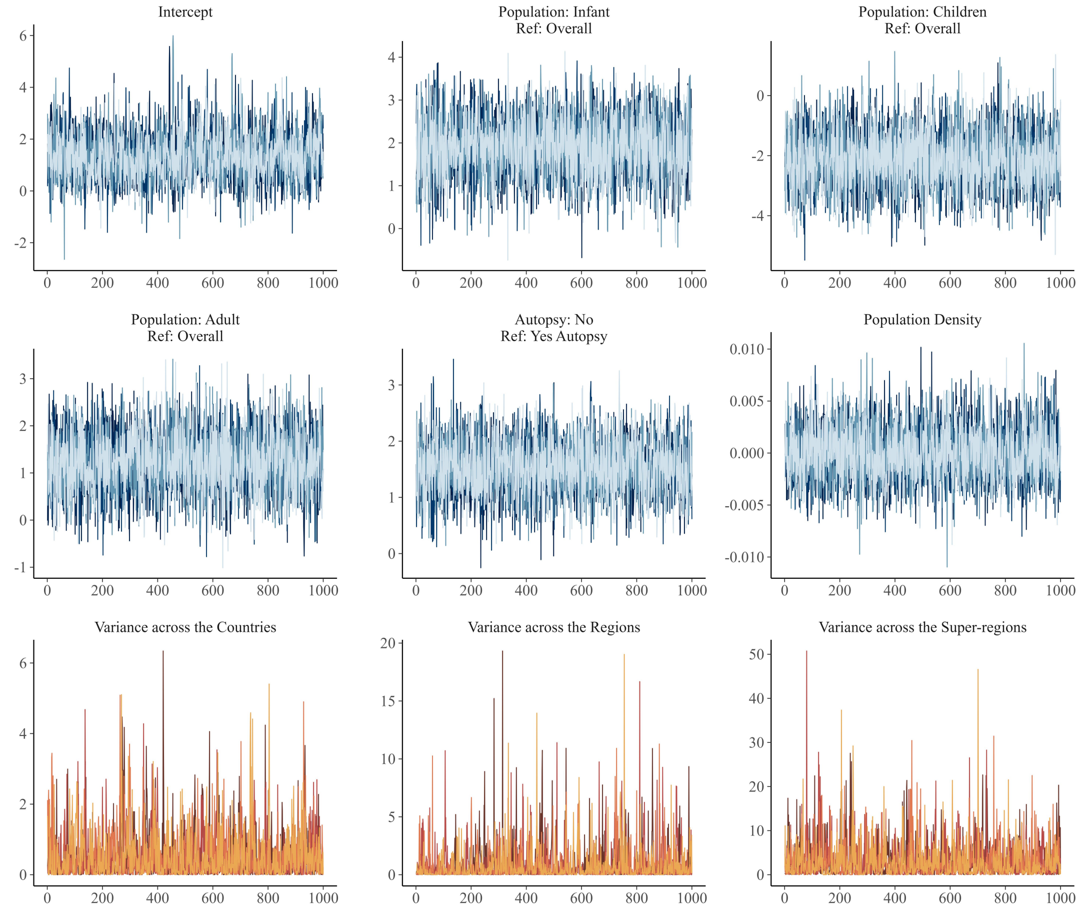

Figure S5. Posterior predictive check plot comparing observed and simulated values from the Bayesian hierarchical model. In the plot, y denotes the observed value, and y_{rep} denotes the fitted value.

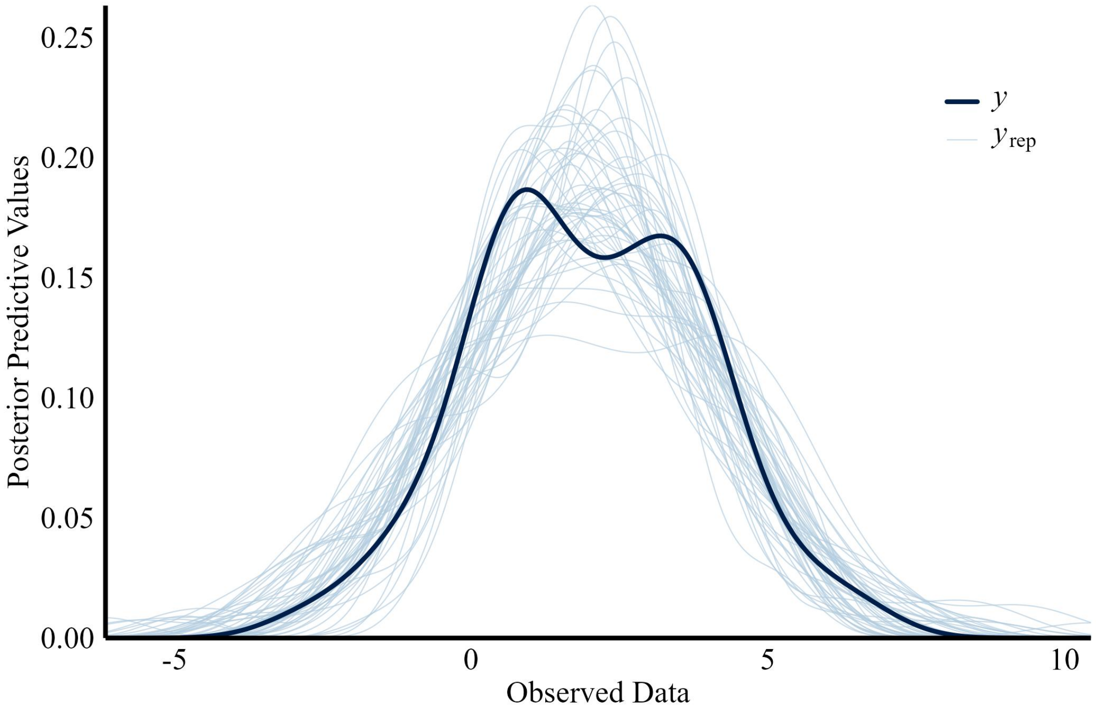

Figure S6. Estimated incidence of sudden death for infants in 21 regions. The error bars indicate 95% credible intervals (95% CI). The different color represents the different super region, respectively.

Region	Incidence rate	Incidence rate log transformed (95% CI)
Europe, central	127.15	4.85 (2.61, 7.56)
Asia,central	20.09	3.00 (0.68, 5.65)
Europe, eastern	174.11	5.16 (2.82, 7.83)
Europe, western	63.71	4.15 (2.63, 5.59)
Australasia	61.63	4.12 (2.38, 5.51)
North America, high income	62.25	4.13 (2.52, 5.49)
Asia Pacific, high income	46.45	3.84 (−5.15, 13.01)
Latin America, southern	144.88	4.98 (3.32, 6.46)
Latin America, tropical	178.55	5.18 (2.93, 8.85)
Caribbean	206.18	5.33 (2.99, 8.80)
Latin America, Andean	249.16	5.52 (3.08, 9.12)
Latin America, central	107.88	4.68 (2.33, 8.23)
North Africa and the Middle East	69.14	4.24 (1.44, 6.32)
Asia, south	310.54	5.74 (3.28, 9.85)
Asia, east	166.70	5.12 (3.13, 7.60)
Asia, South East	177.72	5.18 (3.21, 8.15)
Oceania	83.30	4.42 (2.42, 6.95)
Sub−Saharan Africa,western	39.06	3.67 (−0.28, 5.74)
Sub−Saharan Africa, central	17.26	2.85 (−0.85, 5.09)
Sub−Saharan Africa,southern	28.20	3.34 (−0.38, 5.57)
Sub−Saharan Africa,eastern	28.59	3.35 (−0.59, 5.60)

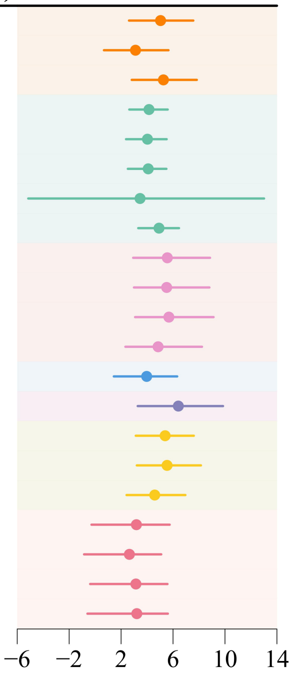

Figure S7. Estimated incidence of sudden death for children in 21 regions. The error bars indicate 95% credible intervals (95% CI). The different color represents the different super region, respectively.

Region	Incidence rate	Incidence rate log transformed (95% CI)
Europe, central	2.76	1.02 (−1.73, 3.87)
Asia,central	0.40	−0.92 (−3.63, 2.13)
Europe, eastern	3.47	1.24 (−1.48, 4.30)
Europe, western	1.15	0.14 (−1.82, 2.10)
Australasia	1.01	0.01 (−2.16, 2.14)
North America, high income	1.06	0.06 (−1.92, 2.00)
Asia Pacific, high income	0.63	−0.46 (−9.34, 8.88)
Latin America, southern	2.45	0.89 (−1.23, 3.03)
Latin America, tropical	4.73	1.55 (−1.39, 5.14)
Caribbean	4.55	1.52 (−1.44, 5.06)
Latin America, Andean	5.31	1.67 (−1.26, 5.39)
Latin America, central	2.35	0.85 (−2.05, 4.48)
North Africa and the Middle East	0.97	−0.03 (−2.89, 2.72)
Asia, south	10.95	2.39 (−0.95, 6.22)
Asia, east	3.94	1.37 (−1.09, 3.93)
Asia, South East	4.59	1.52 (−1.10, 4.50)
Oceania	1.79	0.58 (−1.90, 3.30)
Sub−Saharan Africa,western	0.41	−0.88 (−4.56, 2.21)
Sub−Saharan Africa, central	0.24	−1.41 (−5.17, 1.55)
Sub−Saharan Africa,southern	0.40	−0.92 (−4.68, 2.05)
Sub−Saharan Africa,eastern	0.42	−0.88 (−4.80, 2.06)

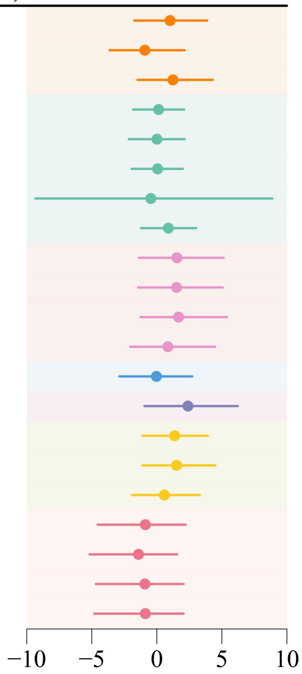

Figure S8. Estimated incidence of sudden death for adults in 21 regions. The error bars indicate 95% credible intervals (95% CI). The different color represents the different super region, respectively.

Region	Incidence rate	Incidence rate log transformed (95% CI)
Europe, central	79.75	4.38 (2.08, 6.88)
Asia,central	11.16	2.41 (0.12, 5.08)
Europe, eastern	96.98	4.57 (2.26, 7.25)
Europe, western	32.44	3.48 (2.24, 4.74)
Australasia	28.66	3.36 (1.77, 4.89)
North America, high income	30.35	3.41 (2.03, 4.71)
Asia Pacific, high income	17.62	2.87 (−5.33, 11.79)
Latin America, southern	69.91	4.25 (2.76, 5.78)
Latin America, tropical	134.05	4.90 (2.19, 8.24)
Caribbean	124.40	4.82 (2.46, 8.10)
Latin America, Andean	147.60	4.99 (2.52, 8.51)
Latin America, central	64.54	4.17 (1.75, 7.60)
North Africa and the Middle East	27.08	3.30 (0.98, 5.48)
Asia, south	304.36	5.72 (2.72, 9.39)
Asia, east	108.92	4.69 (2.70, 6.85)
Asia, South East	128.39	4.86 (2.57, 7.49)
Oceania	50.83	3.93 (1.81, 6.30)
Sub−Saharan Africa,western	12.00	2.48 (−0.63, 4.90)
Sub−Saharan Africa, central	6.96	1.94 (−1.23, 4.34)
Sub−Saharan Africa,southern	11.46	2.44 (−0.72, 4.85)
Sub−Saharan Africa,eastern	12.37	2.52 (−0.85, 4.90)

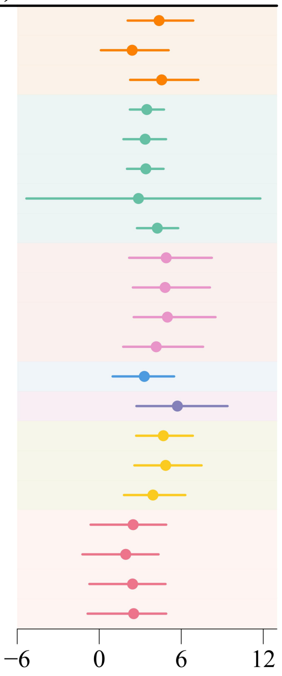

Table S1. Search strategies for the selection of literature sources in this systematic review.

**Literature Search Strategies**	**PubMed Search Strategy**			**Embase Search Strategy**			**Cochrane Library Search Strategy**			**Web of Science Search Strategy**		
Undertaken Jul 20, 2023	***Step***	***Query***	***Results***	***Step***	***Query***	***Results***	***Step***	***Query***	***Results***	***Step***	***Query***	***Results***
	#1	("Death,Sudden/epidemiology"[Mesh] OR "Death, Sudden/ethnology"[Mesh] OR "Death, Sudden/etiology"[Mesh] OR "Death, Sudden/pathology"[Mesh] OR "Death, Sudden/prevention and control"[Mesh])	28,939	#1	'sudden death'/exp OR 'sudden death':ab,ti,kw	92,522	#1	MeSH descriptor: [Death, Sudden] explode all trees and with qualifier(s): [epidemiology - EP)	290	#1	((TS=(Death, sudden)) AND AB=(Sudden death)) AND DOP=(2003-07-01/2023-06-30) and Preprint Citation Index (Exclude – Database)	35,141
	#2	#1 AND (("2003/07/01"[Date - Publication]: "2023/06/30"[Date - Publication]))) AND (English [Language])) AND (Sudden death [Title/Abstract])	3,646	#2	'epidemiology'/exp OR 'epidemiology' OR 'ethnology'/exp OR 'ethnology' OR 'etiology'/exp OR 'etiology' OR 'pathology'/exp OR 'pathology' OR 'prevention and control'/exp OR 'prevention and control'	13,480,093	#2	(Death,Sudden):ti.abkw (Word variations have been searched)	1,088	#2	#1and Article or Review Article or Case Report or Meeting (Document Types)	*33,437*
	#3	#2 AND human Filters: Books and Documents, Review, Systematic Review	851	#3	1 AND 2	55,677	#3	(Death.Sudden) ti.ab kw OR (Death Sudden) tiab,kw OR (Death Sudden) ti ab.kw OR (Death. Sudden) tfiab. ku	1,088	#3	#2 and Humans (MeSH Headings) and Epidemiology or Mortality or Analysis (MeSH Qualifiers)	7,528
				#4	#1AND #32AND ([cochrane review]/lim OR [systematic review]/lim) AND [humans]/lim AND [english]/lim AND [01-07-2003]/sd NOT [30-06-2023]/sd Management, Drug Therapy, Epidemiology, Therapy]	1,082	#4	(Death, sudden): ti,ab,kw AND English:la	3,668	#4	#3 and English (Languages)	7,336
				#5			#5	(Incidence OR Prevalence OR Mortality OR Epidemiology):ti,ab,kw	322,592			
				#6			#6	#1 OR #3 with Cochrane Library publication date from Jul 2003 to Jun 2023	1,094			
							#7	#6 AND #4	766			
							#8	#7 AND #5	528			
	PubMed、 Cochrane Library、 Embase and Web of Science combined<br>No. of duplicates											9797<br>829
	**COMBINED TOTAL FROM FOUR SEARCHES**											**8968**

Table S2. Studies excluded during screening.

Reason for exclusion	Number of studies
Total excluded studies at database screening	905
Before July 1, 2003	76
Duplicates	829
Total excluded studies at title-abstract screening	8589
Wrong object and domain/wrong study design/no study sample/wrong study outcome	8589
Total excluded studies at full-text screening	195
Lack of basic information	61
No relevant target data	41
Redundant information	26
Unable to extract or use data	24
Full text not available	21
Data or literature duplication	20
Unreliable literature	2

Table S3. Studies excluded post-full text screening.

1. Fishbein MC. Cardiac disease and risk of sudden death in the young: the burden of the phenomenon. Cardiovascular Pathology 2010; 19(6): 326–328.	No relevant target data
2. Ågesen FN, Risgaard B, Zachariasardóttir S, et al. Sudden unexpected death caused by stroke: A nationwide study among children and young adults in Denmark. International Journal of Stroke 2018; 13(3): 285–291.	Data or literature duplication
3. Sethi NJ, Safi S, Korang SK, et al. Antibiotics for secondary prevention of coronary heart disease. Cochrane Database of Systematic Reviews 2021; 2(2): CD003610.	No relevant target data
4. Aljinović J, Novak K, Mirić L, Grandić L, Kunac N, Pisac VP. The epidemiology of non-traumatic prehospital sudden death in Split-Dalmatia County. Coll Antropol 2013; 37(4): 1127–1131.	Full text not available
5. Malhotra A, Sharma S. Outcomes of cardiac screening in adolescent soccer players. New England Journal of Medicine 2018; 379(21): 2084.	Lack of basic information
6. Egger F, Scharhag J, Kästner A, Dvořák J, Bohm P, Meyer T. FIFA Sudden Death Registry (FIFA-SDR): a prospective, observational study of sudden death in worldwide football from 2014 to 2018. British Journal of Sports Medicine 2020;56(2):80-87.	Unable to extract or use data
7. Braund S, Leviel J, Morau E, Deneux-Tharaux C, Verspyck E; ENCMM study group. Maternal sudden death: A nationwide retrospective study. BJOG 2023; 130(3): 257–263.	Lack of basic information
8. Krahn AD, Connolly SJ, Roberts RS, Gent M. Diminishing proportional risk of sudden death with advancing age: implications for prevention of sudden death. American Heart Journal 2004;147(5):837-840.	No relevant target data
9. Morentin B, Audicana C. Population-based study of out-of-hospital sudden cardiovascular death: incidence and causes of death in middle-aged adults. Revista Española de Cardiología 2011;64(1):28-34.	No relevant target data
10. Savopoulos C, Ziakas A, Hatzitolios A, et al. Circadian rhythm in sudden cardiac death: a retrospective study of 2,665 cases. Angiology 2006;57(2):197-204.	Lack of basic information
11. Eckart RE, Scoville SL, Shry EA, Potter RN, Tedrow U. Causes of sudden death in young female military recruits. American Journal of Cardiology 2006;97(12):1756-1758.	Lack of basic information
12. Whang W, Manson JE, Hu FB, et al. Physical exertion, exercise, and sudden cardiac death in women. Journal of the American Medical Association 2006;295(11):1399-1403.	Lack of basic information
13. El-Assaad I, Al-Kindi SG, Aziz PF. Trends of out-of-hospital sudden cardiac death among children and young adults. Pediatrics 2017;140(6).	Full text not available
14. Winkel BG. Sudden cardiac death in young Danes. Danish Medical Journal 2012; 59(1): B4403.	Data or literature duplication
15. Morau E, Beaumont E, Verspyck E. Mortalité maternelle par mort subite. Résultats de l'enquête nationale française confidentielle sur la mortalité maternelle, 2010–2012 [Maternal deaths due to sudden death. Results from the French confidential enquiry into maternal deaths, 2010–2012]. Gynecologie Obstetrique Fertilite Senologie 2017; 45(12S): S81–S83.	Unreliable literature
16. Winkel BG, Holst AG, Theilade J, et al. Sudden unexpected death in infancy in Denmark. Scandinavian Cardiovascular Journal 2010; 45(1): 14–20.	Lack of basic information
17. Eckart RE, Shry EA, Burke AP, et al. Sudden death in young adults: an autopsy-based series of a population undergoing active surveillance. Journal of the American College of Cardiology 2011; 58(12): 1254–1261.	Lack of basic information
18. Chow KM, Szeto CC, Kwan BC-H, Chung KY, Leung CB, Li PK-T. Factors associated with sudden death in peritoneal dialysis patients. Peritoneal Dialysis International 2009; 29(1): 58–63.	No relevant target data

19. Turakhia M, Tseng ZH. Sudden cardiac death: epidemiology, mechanisms, and therapy. Current Problems in Cardiology 2007; 32(10): 501–546.	Redundant information
20. Winkel BG, Holst AG, Theilade J, et al. Nationwide study of sudden cardiac death in persons aged 1–35 years. European Heart Journal 2011; 32(8): 983–990.	Data or literature duplication
21. Behr ER, Casey A, Sheppard M, et al. Sudden arrhythmic death syndrome: a national survey of sudden unexplained cardiac death. Heart 2007; 93(6): 601–605.	Lack of basic information
22. Adnet F, Renault R, Jabre P, Kulstad E, Galinski M, Lapostolle F. Incidence of acute myocardial infarction resulting in sudden death outside the hospital. Emergency Medicine Journal 2010; 28(8): 884–886.	Lack of basic information
23. Tseng ZH, Ramakrishna S, Salazar JW, Vittinghoff E, Olgin JE, Moffatt E. Sex and racial differences in autopsy-defined causes of presumed sudden cardiac death. Circulation Arrhythmia and Electrophysiology 2021; 14(3): e120.	Lack of basic information
24. Harmon KG, Drezner JA, Maleszewski JJ, et al. Pathogeneses of sudden cardiac death in National Collegiate Athletic Association athletes. Circulation 2020; 12(1): 123–129.	Unable to extract or use data
25. Shapiro-Mendoza CK, Tomashek KM, Anderson RN, Wingo J. Recent national trends in sudden unexpected infant deaths: more evidence supporting a change in classification or reporting. American Journal of Epidemiology 2006; 163(5): 762–769.	Lack of basic information
26. Welten SJGC, Remmelzwaal S, Blom MT, et al. Validation of the ARIC prediction model for sudden cardiac death in the European population: the ESCAPE-NET project. American Heart Journal 2023; 262(4): 55–65.	Lack of basic information
27. Carrick C, Collins KA, Lee CJ, Prahlow JA, Barnard JJ. Sudden death due to asphyxia by esophageal polyp: two case reports and review of asphyxial deaths. American Journal of Forensic Medicine and Pathology 2005; 26(3): 275–281.	No relevant target data
28. De Visme S, Chalumeau M, Levieux K, et al. National variations in recent trends of sudden unexpected infant death rate in Western Europe. Journal of Pediatrics 2020; 226(7): 179–185.e4.	No relevant target data
29. Monasterio E, McKean A, Sinhalage V, Frampton C, Mulder R. Sudden death in patients with serious mental illness. Psychiatry Research 2018; 131(1): 70–79.	No relevant target data
30. Wong CX, Brown A, Lau DH, et al. Epidemiology of sudden cardiac death: global and regional perspectives. Heart Lung and Circulation 2019; 28(10): 6–14.	Redundant information
31. Lynge TH, Nielsen JL, Blanche P, et al. Decline in incidence of sudden cardiac death in the young: a 10-year nationwide study of 8756 deaths in Denmark. Europace 2019; 21(9): 909–917.	Redundant information
32. Bogle BM, Ning H, Goldberger JJ, Mehrotra S, Lloyd-Jones DM. A simple community-based risk-prediction score for sudden cardiac death. American Journal of Medicine 2018; 131(4): 532–539.e5.	Redundant information
33. Welten SJGC, Remmelzwaal S, Blom MT, et al. Validation of the ARIC prediction model for sudden cardiac death in the European population: the ESCAPE-NET project. American Heart Journal 2023; 262(5): 55–65.	Data or literature duplication
34. Crandall LG, Lee JH, Stainman R, Friedman D, Devinsky O. Potential role of febrile seizures and other risk factors associated with sudden deaths in children. JAMA Network Open 2019; 2(6): e192739.	Unable to extract or use data
35. Udnoon J, Chirachariyavej T, Peonim V. Sudden unexpected deaths in different age groups at Ramathibodi Hospital, Bangkok, Thailand: a retrospective autopsy study during 2003–2007. Journal of the Medical Association of Thailand 2009; 40(2): 162–168.	Full text not available
36. Landi K, Gutierrez C, Sampson B, et al. Investigation of the sudden death of infants: a multicenter analysis. Pediatric and Developmental Pathology 2005; 8(3): 630–638.	Lack of basic information

37. Butters A, Arnott C, Sweeting J, Winkel BG, Semsarian C, Ingles J. Sex disparities in sudden cardiac death. Circulation Arrhythmia and Electrophysiology 2021; 14(8): e008345.	Redundant information
38. Yildiz A, Gürpinar SS, Yağci FE, Çayli E, Baydar ÇL. Retrospective analysis of sudden cardiac deaths in a 10-year autopsy series in the city of Isparta in Turkey. American Journal of Forensic Medicine and Pathology 2020; 41(11): 263–268.	Full text not available
39. Risgaard B, Winkel BG, Jabbari R, et al. Burden of sudden cardiac death in persons aged 1 to 49 years: nationwide study in Denmark. Circulation Arrhythmia and Electrophysiology 2014; 7(4): 205–211.	Data or literature duplication
40. Gervacio-Domingo G, Punzalan FE, Amarillo ML, Dans A. Sudden unexplained death during sleep occurred commonly in the general population in the Philippines: a sub study of the National Nutrition and Health Survey. Journal of Clinical Epidemiology 2007; 60(5): 567–571.	No relevant target data
41. Törő K, Bartholy J, Pongrácz R, Kis Z, Keller É, Dunay G. Evaluation of meteorological factors on sudden cardiovascular death. Journal of Forensic and Legal Medicine 2010; 17(7): 236–242.	Redundant information
42. Neychev VK, Jinnah HA. Sudden death in Lesch-Nyhan disease. Developmental Medicine and Child Neurology 2006; 48(10): 923.	Lack of basic information
43. Allen K, Anderson TM, Chajewska U, Ramirez J, Mitchell EA. Factors associated with age of death in sudden unexpected infant death. Acta Paediatrica 2020; 110(8): 174–183.	Lack of basic information
44. Ventura F, Barranco R, Smith A, et al. Multidisciplinary study of sudden unexpected infant death in Liguria (Italy): a nine-year report. Minerva Pediatrica 2021; 73(3): e156.	Full text not available
45. Whitman IR, Feldman HI, Deo R. CKD and sudden cardiac death: epidemiology, mechanisms and therapeutic approaches. Journal of the American Society of Nephrology 2012; 23(4): 1929–1939.	Redundant information
46. Harris KM, Creswell LL, Haas TS, et al. Death and cardiac arrest in U.S. triathlon participants 1985 to 2016: a case series. Annals of Internal Medicine 2017; 167(8): 529–535.	Unable to extract or use data
47. Kawato H, Hitosugi M, Kido M, Yufu T, Nagai T, Tokudome S. An autopsy case of sudden death in a boy with primary pulmonary hypertension: a case report. Medicine Science and the Law 2005; 45(4): 361–363.	Lack of basic information
48. Möllborg P, Wennergren G, Almqvist P, Alm B. Bed sharing is more common in sudden infant death syndrome than in explained sudden unexpected deaths in infancy. Acta Paediatrica 2015; 104(9): 777–783.	Redundant information
49. Svane J, Lynge TH, Hansen CJ, Risgaard B, Winkel BG, Tfelt-Hansen J. Witnessed and unwitnessed sudden cardiac death: a nationwide study of persons aged 1–35 years. Europace 2021; 23(12): 898–906.	Data or literature duplication
50. Mehra R. Global public health problem of sudden cardiac death. Journal of Electrocardiology 2007; 40(Suppl 1): S118–S122.	Lack of basic information
51. Reinier K, Rusinaru C, Chugh SS. Race, ethnicity and the risk of sudden death. Trends in Cardiovascular Medicine 2019; 29(11): 120–126.	Redundant information
52. Mitchell EA, Yan X, Ren SY, et al. Geographic variation in sudden unexpected infant death in the United States. Journal of Pediatrics 2020; 220(5): 49–55.e2.	Unable to extract or use data
53. Ahmed S, Mitchell I, Wolbring G. Analysis of sudden infant death syndrome coverage in Canadian newspapers. Journal of Child Health Care 2018; 22(9): 545–562.	Redundant information
54. Winkel BG, Risgaard B, Bjune T, et al. Gender differences in sudden cardiac death in the young - a nationwide study. BMC Cardiovascular Disorders 2017; 17(6): 6.	Data or literature duplication
55. Zhao D, Post WS, Blasco-Colmenares E, et al. Racial differences in sudden cardiac death. Circulation 2019; 139(12): 1688–1697.	No relevant target data

56. Zipes DP. Epidemiology and mechanisms of sudden cardiac death. Journal of the American College of Cardiology 2005; 21(Suppl A): 37A–40A.	Full text not available
57. Mounsey LA, Lin FC, Pursell I, et al. Relation of household income to incidence of sudden unexpected death in Wake County, North Carolina. American Journal of Cardiology 2017; 119(7): 1030–1035.	Lack of basic information
58. Freemantle CJ, Read AW, de Klerk NH, McAullay D, Anderson IP, Stanley FJ. Sudden infant death syndrome and unascertainable deaths: trends and disparities among Aboriginal and non-Aboriginal infants born in Western Australia from 1980 to 2001 inclusive. Journal of Paediatrics and Child Health 2006; 42(4): 445–451.	Unable to extract or use data
59. Tavora F, Zhang Y, Zhang M, et al. Cardiomegaly is a common arrhythmogenic substrate in adult sudden cardiac deaths and is associated with obesity. Pathology 2012; 44(2): 187–191.	Lack of basic information
60. Omalu BI, Lindner JL, Janssen JK, et al. The role of environmental factors in the causation of sudden death in infants: two cases of sudden unexpected death in two unrelated infants who were cared for by the same babysitter. Journal of Forensic Sciences 2007; 52(6): 1355–1358.	Lack of basic information
61. Freemantle CJ, Read AW, de Klerk NH, Charles A, McAullay D, Stanley F. Interpretation of recent sudden infant death syndrome rates in Western Australia. Journal of Paediatrics and Child Health 2005; 41(12): 669–670.	Lack of basic information
62. Tereshchenko LG, Kim ED, Oehler A, et al. Electrophysiologic substrate and risk of mortality in incident hemodialysis. Journal of the American Society of Nephrology 2016; 27(11): 3413–3420.	No relevant target data
63. Zachariasardóttir S, Risgaard B, Ågesen FN, et al. Sudden cardiac death and coronary disease in the young: a nationwide cohort study in Denmark. International Journal of Cardiology 2017; 236(1): 16–22.	Data or literature duplication
64. Stecker EC, Reinier K, Marijon E, et al. Public health burden of sudden cardiac death in the United States. Circulation Arrhythmia and Electrophysiology 2014; 7(2): 212–217.	Unable to extract or use data
65. Wren C. Sudden death in children and adolescents. British Heart Journal 2000; 83(4): 410–413.	Redundant information
66. Montagnana M, Lippi G, Franchini M, Targher G, Guidi GC. Sudden cardiac death: prevalence, pathogenesis, and prevention. Annals of Medicine 2008; 40(5): 360–375.	Redundant information
67. Yousuf O, Chrispin J, Tomaselli GF, Berger RD. Clinical management and prevention of sudden cardiac death. Circulation Research 2015; 116(12): 2020–2040.	Unable to extract or use data
68. Chowdhury PS, Franklin BA, Boura JA, et al. Sudden cardiac death after manual or automated snow removal. American Journal of Cardiology 2003; 92(7): 833–835.	Lack of basic information
69. Fiscal-Málaga AG, Sosa-Cruz H, Calderón-Garcidueñas AL, et al. Sudden death report in Mexico (1998-2014). Reporte de muerte súbita en Mexico (1998-2014). Archivos de Cardiología de México 2019; 89(2): 154–158.	Full text not available
70. Jabbari R, Risgaard B, Holst AG, et al. Cardiac symptoms before sudden cardiac death caused by coronary artery disease: a nationwide study among young Danish people. Heart 2013; 99(13): 938–943.	Data or literature duplication
71. Randall B, Thompson P, Wilson A. Racial differences within subsets of sudden unexpected infant death (SUID) with an emphasis on asphyxia. Journal of Forensic and Legal Medicine 2019; 62: 52–55.	No relevant target data
72. Lynge TH, Nielsen JL, Blanche P, et al. Decline in incidence of sudden cardiac death in the young: a 10-year nationwide study of 8756 deaths in Denmark. Europace 2019; 21(9): 909–917.	Data or literature duplication
73.Loland VW, Ågesen FN, Lynge TH, et al. Low birth weight increases the risk of sudden cardiac death in the young: a nationwide study of 2.2 million people. Journal of the American Heart Association 2021; 10(2): e018314.	Data or literature duplication
74. Johnston R, Yan X, Anderson TM, Mitchell EA. Altitude and risk of sudden unexpected infant death in the United States. Scientific Reports 2021; 11(1): 2161.	Lack of basic information

75. Wisten A, Börjesson M, Krantz P, Stattin EL. Exercise related sudden cardiac death (SCD) in the young: pre-mortal characterization of a Swedish nationwide cohort showing a decline in SCD among athletes. Resuscitation 2019; 144(1): 99–105.	Data or literature duplication
76. Hayashi M, Shimizu W, Albert CM. The spectrum of epidemiology underlying sudden cardiac death. Circulation Research 2015; 116(12): 1887–1906.	Redundant information
77. Hayashi M, Shimizu W, Albert CM. Febrile seizures prior to sudden cardiac death: a Danish nationwide study. Circulation Research 2015; 116(12): 1887–1906.	Data or literature duplication
78. Tomcik MA, Gerig NR, Prahlow JA. Sudden death from ruptured intracranial vascular malformation. Forensic Science, Medicine, and Pathology 2010; 7(2): 185–191.	Redundant information
79. Link MS. Sudden cardiac death in the young: epidemiology and overview. Congenital Heart Disease 2017; 12(5): 597–599.	Redundant information
80. Lynge TH, Svane J, Pedersen-Bjergaard U, et al. Sudden cardiac death among persons with diabetes aged 1-49 years: a 10-year nationwide study of 14294 deaths in Denmark. European Heart Journal 2020; 41(23): 2699–2706.	Data or literature duplication
81. Ha FJ, Han H-C, Sanders P, et al. Sudden cardiac death related to physical exercise in the young: a nationwide cohort study of Australia. Internal Medicine Journal 2022; 53(1): 497–502.	Full text not available
82.Torrisi M, Pennisi G, Russo I, Amico F, Esposito M. Sudden cardiac death in anabolic-androgenic steroid users: a literature review. Medicina (Kaunas, Lithuania) 2020; 56(11): 587.	Lack of basic information
83. Behr ER, Scrocco C, Wilde AAM, et al. Investigation on sudden unexpected death in the young (SUDY) in Europe: results of the European Heart Rhythm Association survey. Europace 2022; 24(3): 331–339.	Lack of basic information
84. Krexi L, Georgiou R, Krexi D, Sheppard MN. Sudden cardiac death with stress and restraint: the association with sudden adult death syndrome cardiomyopathy and coronary artery disease. Medicine, Science and the Law 2015; 56(2): 85–90.	Full text not available
85. Steinhaus DA, Vittinghoff E, Moffatt E, Hart AP, Ursell P, Tseng ZH. Characteristics of sudden arrhythmic death in a diverse urban community. American Heart Journal 2012; 163(1): 125–131.	Lack of basic information
86. Salvo F, Pariente A, Shakir S, et al. Sudden cardiac and sudden unexpected death related to antipsychotics: a meta-analysis of observational studies. Clinical Pharmacology & Therapeutics 2016; 99(3): 306–314.	Full text not available
87.Chapur V. Epidemiology of sudden unexpected death in infancy in Argentina: secular trend and spatial variation. Archives of Argentine Pediatrics 2019; 117(3): e164.	No relevant target data
88. Müller-Nordhorn J, Schneider A, Grittner U, et al. International time trends in sudden unexpected infant death 1969–2012. BMC Pediatrics 2020; 20(1): 377.	Redundant information
89. Stiffler D, Ayres B, Fauvergue C, Cullen D. Sudden infant death and sleep practices in the Black community. Journal of Specialist Pediatric Nursing 2018; 23(1): e12213.	No relevant target data
90. Brockmann PE, Oyarzún MA, Villarroel L, Bertrand P. Síndrome de muerte súbita del lactante: prevalencia y cambios en los últimos años en Chile [Prevalence of sudden death syndrome among children]. Revista Medica de Chile 2013; 141(5): 589–594.	Lack of basic information
91. Skinner JR, Crawford J, Smith W, et al. Prospective population-based long QT molecular autopsy study of postmortem negative sudden death in 1 to 40 year olds. Heart Rhythm 2011; 8(4): 412–419.	Redundant information
92. Erck Lambert AB, Parks SE, Shapiro-Mendoza CK. National and state trends in sudden unexpected infant death: 1990-2015. Pediatrics 2018; 141(3): e20173519.	Unable to extract or use data
93. Hendrix A, Borleffs CJW, Vink A, et al. Cardiogenetic screening of first-degree relatives after sudden cardiac death in the young: a population-based approach. Europace 2011; 13(5): 716–722.	Unable to extract or use data

94. Isbey SC, Howard MB, Abdulrahman E, et al. Characteristics and geographic variation in sudden unexpected infant deaths in the district of Columbia. American Journal of Forensic Medicine and Pathology 2022; 43(4): 328–333.	Full text not available
95. Garstang J, Ellis C, Griffiths F, Sidebotham P. Unintentional asphyxia SIDS and medically explained deaths: a descriptive study of outcomes of child death review (CDR) investigations following sudden unexpected death in infancy. Forensic Science Medicine and Pathology 2016; 12(4): 407–415.	Lack of basic information
96. Sweeting J, Semsarian C. Sudden cardiac death in athletes. Heart Lung and Circulation 2018; 27(9): 1072–1077.	Full text not available
97. Lynge TH, Risgaard B, Banner J, et al. Nationwide burden of sudden cardiac death: a study of 54028 deaths in Denmark. Heart Rhythm 2021; 18(10): 1657–1665.	Data or literature duplication
98. Sakai K, Maruyama-Maebashi K, Takatsu A, et al. Sudden death involving inhalation of 11-difluoroethane (HFC-152a) with spray cleaner: three case reports. Forensic Science International 2011; 206(1-3): e58–61.	Redundant information
99. Murakoshi N, Aonuma K. Epidemiology of arrhythmias and sudden cardiac death in Asia. Circulation Journal 2013; 77(10): 2419–2431.	Unable to extract or use data
100. Codina P, Zamora E, Levy WC, et al. Sudden cardiac death in heart failure: a 20-year perspective from a Mediterranean cohort. Journal of Cardiac Failure 2023; 29(2): 236–245.	Lack of basic information
101. Moon RY, Sprague BM, Patel KM. Stable prevalence but changing risk factors for sudden infant death syndrome in child care settings in 2001. Pediatrics 2005; 116(5): 972–977.	Lack of basic information
102. Mahmoud KD, De Smet BJGL, Zijlstra F, Rihal CS, Holmes DR. Sudden cardiac death: epidemiology, circadian variation, and triggers. Current Problems in Cardiology 2011; 36(2): 56–80.	No relevant target data
103. Wisten A, Messner T. Young Swedish patients with sudden cardiac death have a lifestyle very similar to a control population. Scandinavian Cardiovascular Journal 2005; 39(3): 137–142.	Lack of basic information
104. Chinen K, Kurosumi M, Ohkura Y, Sakamoto A, Fujioka Y. Sudden unexpected death in patients with malignancy: A clinicopathologic study of 28 autopsy cases. Pathology Research and Practice 2006; 202(11): 869–875.	Lack of basic information
105. Shi GQ, Huang WL, Zhang J, Zhao H, Shen T. Clusters of sudden unexplained death associated with the mushroom Trogia venenata in rural Yunnan Province, China. PLoS One 2012; 7(5): e35894.	No relevant target data
106. De Jonge GA. Sudden infant death syndrome in child care settings in the Netherlands. Archives of Disease in Childhood 2004; 89(5): 427–430.	No relevant target data
107. Toreyhi H, Asgari S, Khalili D, Pishghahi M, Azizi F, Hadaegh F. Sudden cardiac death among Iranian population: a two decades follow-up of Tehran lipid and glucose study. Scientific Reports 2021; 11(1): 95210.	Lack of basic information
108. Halloran D, Alexander G. Preterm delivery and age of SIDS death. Annals of Epidemiology 2006; 16(8): 600–606.	Unable to extract or use data
109. De Faria Oliveira AM, De Andrade PR, Pinheiro EM, et al. Risk and protective factors for sudden infant death syndrome. Revista Brasileira de Enfermagem 2020; 73(6): e20190458.	No relevant target data
110. Takada A, Saito K, Nagai T, Hamamatsu A, Murai T. When does an infarcted heart rupture? A pathological study of 148 out-of-hospital sudden death cases. International Journal of Cardiology 2008; 129(3): 447–448.	Lack of basic information
111. Miao Q, Zhang YL, Miao QF, et al. Sudden death from ischemic heart disease while driving: cardiac pathology, clinical characteristics, and countermeasures. Medical Science Monitor 2020; 26(12): e929212.	Lack of basic information
112. Abildstrom SZ, Ottesen MM, Rask-Madsen C, et al. Sudden cardiovascular death following myocardial infarction: the importance of left ventricular systolic dysfunction and congestive heart failure. International Journal of Cardiology 2005; 104(2): 184–189.	No relevant target data

113. Li L, Fowler D, Liu L, Ripple MG, Lambros Z, Smialek JE. Investigation of sudden infant deaths in the State of Maryland (1990-2000). Forensic Science International 2005; 148(2-3): 85–92.	Lack of basic information
114. Rozen G, Elbaz-Greener G, Marai I, et al. The relationship between the body mass index and in-hospital mortality in patients admitted for sudden cardiac death in the United States. Clinical Cardiology 2021; 44(12): 1673–1682.	Unreliable literature
115. Tseng WC, Wu MH, Chiu SN, Chen HC, Kao FY, Huang SK. Cumulative postnatal risk of pediatric sudden death and all-cause mortality in nationwide birth cohorts of Taiwan and the United States. Journal of the Formosan Medical Association 2020; 119(12): 1842–1853.	Data or literature duplication
116. Fox CS, Evans JC, Larson MG, Kannel WB, Levy D. Temporal trends in coronary heart disease mortality and sudden cardiac death from 1950 to 1999: the Framingham Heart Study. Circulation 2004; 110(5): 522–527.	Lack of basic information
117. Ramireddy A, Chugh HS, Reinier K, et al. Sudden cardiac death during nighttime hours. Heart Rhythm 2021; 18(5): 778–784.	Lack of basic information
118. Christensen ED, Berger J, Alashari MM, et al. Sudden infant death “syndrome”-Insights and future directions from a Utah population database analysis. American Journal of Medical Genetics Part A 2016; 173(1): 177–182.	Lack of basic information
119. Skjelbred T, Rajan D, Svane J, Lynge TH, Tfelt-Hansen J. Sex differences in sudden cardiac death in a nationwide study of 54,028 deaths. Heart 2022; 108(12): 1012–1018.	Unable to extract or use data
120. Subirana MT, Juan-Babot JO, Puig T, et al. Specific characteristics of sudden death in a Mediterranean Spanish population. American Journal of Cardiology 2011; 107(4): 622–627.	Lack of basic information
121. Roi GS. Fatal events related to running competitions in the mountains. Wilderness & Environmental Medicine 2021; 32(3): 176–180.	Lack of basic information
122. Holmström L, Kauppila J, Vähätalo J, et al. Sudden cardiac death after alcohol intake: classification and autopsy findings. Scientific Reports 2022; 12(1): 20250.	Lack of basic information
123. Pan H, Hibino M, Kobeissi E, Aune D. Blood pressure, hypertension and the risk of sudden cardiac death: a systematic review and meta-analysis of cohort studies. European Journal of Epidemiology 2019; 35(5): 443–454.	Redundant information
124. Cheng J, Makielski JC, Yuan P, et al. Sudden unexplained nocturnal death syndrome in Southern China: an epidemiological survey and SCN5A gene screening. American Journal of Forensic Medicine and Pathology 2011; 32(4): 359–363.	Redundant information
125. Mage DT. Seasonal variation of sudden infant death syndrome in Hawaii. Journal of Epidemiology and Community Health 2004; 58(10): 912–916.	Unable to extract or use data
126. Landi KK, Coleman AT. Sudden death in toddlers caused by Influenza B infection: A report of two cases and a review of the literature. Journal of Forensic Sciences 2008; 53(1): 213–215.	No relevant target data
127. Blair PS, Sidebotham P, Berry PJ, Evans M, Fleming PJ. Major epidemiological changes in sudden infant death syndrome: a 20-year population-based study in the UK. Lancet 2006; 367(9507): 314–319.	Lack of basic information
128. Ågesen FN, Lynge TH, Blanche P, et al. Temporal trends and sex differences in sudden cardiac death in the Copenhagen City Heart Study. Heart 2021; 107(17): 1303–1309.	Full text not available

129. Lahtinen M, Kiviniemi AM, Junttila MJ, Kääriäinen M, Huikuri HV, Tulppo MP. Depressive symptoms and risk for sudden cardiac death in stable coronary artery disease. American Journal of Cardiology 2018; 122(5): 749–755.	No relevant target data
130. Ryti NRI, Mäkikyrö EMS, Antikainen H, et al. Risk of sudden cardiac death in relation to season-specific cold spells: a case-crossover study in Finland. BMJ Open 2017; 7(11): e017398.	No relevant target data
131. Hirabayashi M, Yoshinaga M, Nomura Y, et al. Environmental risk factors for sudden infant death syndrome in Japan. European Journal of Pediatrics 2016; 175(12): 1921–1926.	Unable to extract or use data
132.Hansen BL, Jacobsen EM, Kjerrumgaard A, et al. Diagnostic yield in victims of sudden cardiac death and their relatives. Europace 2020; 22(7): 964–971.	Lack of basic information
133.Paratz ED, Van Heusden A, Zentner D, et al. Prevalence of coronary artery anomalies in young and middle-aged sudden cardiac death victims (from a prospective state-wide registry). Am J Cardiol 2022; 175: 127–130.	Data or literature duplication
134. Perkiömäki J, Hookana E, Kaikkonen K, Junttila J, Kortelainen ML, Huikuri H. Blood alcohol in victims of sudden cardiac death in northern Finland. Europace 2015; 18(7): 1006–1009.	Lack of basic information
135. Krous HF, Haas EA, Chadwick AE, Masoumi H, Stanley C. Intrathoracic petechiae in SIDS: a retrospective population-based 15-year study. Forensic Science, Medicine, and Pathology 2008; 4(4): 234–239.	Lack of basic information
136. Shipstone RA, Young J, Kearney L, Thompson JMD. Prevalence of risk factors for sudden infant death among Indigenous and non-Indigenous people in Australia. Acta Paediatrica 2020; 109(12): 2614–2626.	No relevant target data
137. Suzuki M, Shimbo T, Ikaga T, Hori S. Sudden death phenomenon while bathing in Japan - Mortality data. Circulation Journal 2017; 81(8): 1144–1149.	Unable to extract or use data
138. Tajiri T, Tate G, Miura K, et al. Sudden death caused by fulminant bacterial infection: Background and pathogenesis of Japanese adult cases. Internal Medicine 2008; 47(16): 1499–1504.	No relevant target data
139. Risgaard B, Nielsen JB, Jabbari R, et al. Prior myocardial infarction in the young: predisposes to a high relative risk but low absolute risk of sudden cardiac death. Europace 2012; 14(1): 48–54.	Lack of basic information
140. Woida FM, Saggioro FP, Ferro MAR, Peres LC. Sudden infant death syndrome in Brazil: fact or fancy? São Paulo Medical Journal 2008; 126(6): 48–51.	No relevant target data
141. Chen H, Deng Y, Li S. Relation of body mass index categories with risk of sudden cardiac death. International Heart Journal 2019; 60(3): 624–630.	No relevant target data
142. Park S, Han JH, Hwang J, et al. The global burden of sudden infant death syndrome from 1990 to 2019: a systematic analysis from the Global Burden of Disease study 2019. QJM 2022; 115(10): 735–744.	Full text not available
143. Bandoli G, Baer RJ, Owen M, et al. Maternal, infant, and environmental risk factors for sudden unexpected infant deaths: results from a large administrative cohort. Journal of Maternal-Fetal & Neonatal Medicine 2021; 35(5): 8998–9005.	Full text not available
144. Kitulwatte ID, Kim PJH, Pollanen MS. Sudden death related myocarditis: a study of 56 cases. Forensic Science, Medicine, and Pathology 2009; 6(1): 13–19.	No relevant target data

145. Tu SJ, Gallagher C, Elliott AD, et al. Alcohol consumption and risk of ventricular arrhythmias and sudden cardiac death: An observational study of 408712 individuals. Heart Rhythm 2022;<br>19(3): 177–184.	Lack of basic information
146. Chen YY, Chung FP, Lin YJ, Chien KL, Chang WT. Exploring the risk factors of sudden cardiac death using an electrocardiography and medical ultrasonography for the general population<br>without a history of coronary artery disease or left ventricular ejection fraction <35% and aged >35 years - A novel point-based prediction model based on the Chin-Shan Community<br>Cardiovascular Cohort. Circulation Journal 2022; 87(1): 139–149.	Lack of basic information
147.Hendrix A, Vaartjes I, Mosterd A, et al. Regional differences in incidence of sudden cardiac death in the young. European Heart Journal 2010; 31(3): 274–279.	Redundant information
148. Vähätalo JH, Huikuri HV, Holmström LTA, et al. Association of silent myocardial infarction and sudden cardiac death. JAMA Cardiology 2019; 4(8): 796.	No relevant target data
149. Gimeno JR, Oliva MJ, Lacunza J, et al. Characteristics of sudden death in inherited heart disease. Revista Española De Cardiología 2010; 63(3): 268–276.	Lack of basic information
150. Liang J, He Y, Fan L, et al. A preliminary study on the abnormal deaths and work burden of Chinese physicians: A mixed method analysis and implications for smart hospital management.<br>Frontiers in Public Health 2022; 10(4): 10039.	No relevant target data
151. Rosenberg MA, Lopez FL, Bůžková P, et al. Height and risk of sudden cardiac death: the Atherosclerosis Risk in Communities and Cardiovascular Health Studies. Annals of Epidemiology 2014;<br>24(3): 174-179.e2.	No relevant target data
152. Parks SE, Erck Lambert AB, Shapiro-Mendoza CK. Racial and ethnic trends in sudden unexpected infant deaths: United States 1995-2013. Pediatrics 2017; 139(6): e20163844.	Unable to extract or use data
153.MacFarlane M, Thompson JMD, Zuccollo J, et al. Smoking in pregnancy is a key factor for sudden infant death among Māori. Acta Paediatrica 2018; 107(11): 1924–1931.	Lack of basic information
154. Lear A, Patel N, Mullen C, et al. Incidence of sudden cardiac arrest and death in young athletes and military members: a systematic review and meta-analysis. Journal of Athletic Training 2021;<br>57(2): 431–443.	Lack of basic information
155. Fung GP, Lau S, Hui AS, et al. Neonatal mortality in singleton pregnancies: a 20-year retrospective study from a tertiary perinatal unit in Hong Kong. Hong Kong Medical Journal 2022; 28(4):<br>739.	Lack of basic information
156. Wu MH, Chen HC, Wang JK, Chiu HH, Huang SC, Huang SK. Population-based study of pediatric sudden death in Taiwan. Journal of Pediatrics 2009; 155(6): 870-874.e2.	Redundant information
157. Ye S, Grunnert M, Thune JJ, et al. Circumstances and outcomes of sudden unexpected death in patients with high-risk myocardial infarction: implications for prevention. Circulation 2011;<br>123(23): 2674–2680.	No relevant target data
158. Olson KA, Patel RB, Ahmad FS, et al. Sudden cardiac death risk distribution in the United States population (from NHANES, 2005 to 2012). American Journal of Cardiology 2019; 123(9):<br>1249–1254.	No relevant target data

159. Zhang J, Zhou X, Xing Q, et al. Sudden cardiac death in the Kazakh and Han peoples of Xinjiang, China: a comparative cross-sectional study. Medicine (Baltimore) 2019; 98(50): e18126.	Data or literature duplication
160. Filippi A, Sessa E Jr, Mazzaglia G, et al. Out of hospital sudden cardiac death in Italy: a population-based case-control study. Journal of Cardiovascular Medicine (Hagerstown) 2008; 9(6): 595–600.	Unable to extract or use data
161. Litchfield IJ, Ayres JG, Jaakkola JJK, Mohammed NI. Is ambient air pollution associated with onset of sudden infant death syndrome: a case-crossover study in the UK. BMJ Open 2018; 8(4): e018341.	No relevant target data
162. Jhun I, Mata DA, Nordio F, Lee M, Schwartz J, Zanobetti A. Ambient temperature and sudden infant death syndrome in the United States. Epidemiology 2017; 28(5): 728–734.	Unable to extract or use data
163. Isiozor NM, Kunutsor SK, Voutilainen A, Kurl S, Kauhanen J, Laukkanen JA. Association between ideal cardiovascular health and risk of sudden cardiac death and all-cause mortality among middle-aged men in Finland. European Journal of Preventive Cardiology 2021; 28(3): 294–300.	Lack of basic information
164. Correction to: Racial differences in sudden cardiac death: Atherosclerosis Risk in Communities Study (ARIC). Circulation 2019; 139(14): e837.	No relevant target data
165. Kunutsor SK, Zaccardi F, Karppi J, Kurl S, Laukkanen JA. Is high serum LDL/HDL cholesterol ratio an emerging risk factor for sudden cardiac death? Findings from the KIHD Study. Journal of Atherosclerosis and Thrombosis 2017; 24(6): 600–608.	No relevant target data
166. Jiménez-Pavón D, Artero EG, Lee DC, et al. Cardiorespiratory fitness and risk of sudden cardiac death in men and women in the United States: a prospective evaluation from the Aerobics Center Longitudinal Study. Mayo Clinic Proceedings 2016; 91(7): 849–857.	Unable to extract or use data
167. Sharifzadehgan A, Gaye B, Bougouin W, et al. Lack of early etiologic investigations in young sudden cardiac death. Resuscitation 2022; 179(1): 197–205.	Data or literature duplication
168. Shi S, Liu T, Liang J, Hu D, Yang B. Depression and Risk of Sudden cardiac Death and arrhythmias: A Meta-Analysis. Psychosomatic Medicine 2017; 79(2): 153–161.	No relevant target data
169. Tester DJ, Wong LCH, Chanana P, et al. Cardiac genetic predisposition in sudden infant death syndrome. Journal of the American College of Cardiology 2018; 71(12): 1217–1227.	Lack of basic information
170. Lahaye SLD, Cunuder AL, Lachard T, et al. Cardiac events in World-Class Athletes: an Internet-Based study. Medicine and Science in Sports and Exercise 2022; 54(9): 2064–2072.	Full text not available
171. Jensen K, Howell SJ, Phan F, et al. Bringing critical race praxis into the study of electrophysiological substrate of sudden cardiac death: the ARIC Study. Journal of the American Heart Association Cardiovascular and Cerebrovascular Disease 2020; 9(1): e015012.	No relevant target data
172. Ohira T, Maruyama M, Imano H, et al. Risk factors for sudden cardiac death among Japanese: the Circulatory Risk in Communities Study. Journal of Hypertension 2012; 30(6): 1137–1143.	Redundant information
173. Empana J-P, Lerner I, Valentin E, et al. Incidence of sudden cardiac death in the European Union. Journal of the American College of Cardiology 2022; 79(14): 1818–1827.	Unable to extract or use data
174. Shang LX, Zhou XH, Zhang JH, et al. Establishment of a predictive model for inpatient sudden cardiac death in a Chinese cardiac department population: a retrospective study. Chinese Medical	Lack of basic information

Journal 2019; 132(1): 17-24.	
175. Daltveit A, Irgens L, Øyen N, Skjærven R, Markestad T, Wennergren G. Circadian variations in sudden infant death syndrome: associations with maternal smoking, sleeping position, and infections. Acta Paediatrica 2003; 92(8): 1007–1013.	Unable to extract or use data
176. Koyak Z, De Groot JR, Bouma BJ, et al. Sudden cardiac death in adult congenital heart disease: can the unpredictable be foreseen? Europace 2016; 18(3): euw060.	Redundant information
177. Lin YN, Chang SS, Wang LM, et al. Prehospital Predictors of Initial Shockable Rhythm in Out-of-Hospital Cardiac Arrest: Findings From the Taichung Sudden Unexpected Death Registry (THUNDER). Mayo Clinic Proceedings 2017; 92(3): 347-359.	Unable to extract or use data
178. Paratz ED, Rowe SJ, Stub D, Pflaumer A, La Gerche A. A systematic review of global autopsy rates in all-cause mortality and young sudden death. Heart Rhythm 2023; 20(4): 607–613.	Lack of basic information
179. Lakhoo DP, Blake HA, Chersich MF, Nakstad B, Kovats S. The Effect of high and low ambient temperature on infant health: a systematic review. International Journal of Environmental Research and Public Health 2022; 19(17): 9109.	Redundant information
180. Verjee MA, Reimann TA, Alinier G. Sudden cardiac arrest in football. Journal of Emergency Medicine, Trauma & Acute Care 2022; 2022(4): 123-130.	Full text not available
181. Couper K, Putt O, Field R, et al. Incidence of sudden cardiac death in the young: a systematic review. BMJ Open 2020; 10(3): e040815.	No relevant target data
182. 182. Bennett T, Martin LJ, Heathfield LJ. Global trends in the extent of death scene investigation performed for sudden and unexpected death of infant (SUDI) cases: A systematic review. Forensic Science International 2019; 301(1): 435–444.	No relevant target data
183. Li X, Yu H, Tang L, Xie M, Zhang C. Arrhythmic Mitral Valve Prolapse: A Systematic Review and Meta-Analysis of the Association Between Mitral Valve Prolapse and Sudden Cardiac Death. Diagnostics 2023; 13(18): 2868.	Full text not available
184. Aune D, Schlesinger S, Norat T, Riboli E. Diabetes mellitus and the risk of sudden cardiac death: A systematic review and meta-analysis of prospective studies. NMCD Nutrition Metabolism and Cardiovascular Diseases 2018; 28(5): 543–556.	No relevant target data
185. Aune D, Schlesinger S, Norat T, Riboli E. Tobacco smoking and the risk of sudden cardiac death: a systematic review and meta-analysis of prospective studies. European Journal of Epidemiology 2018; 33(6): 509–521.	Lack of basic information
186. Alba AC, Foroutan F, Ng Fat Hing NKV, Fan CS, Manlhiot C, Ross HJ. Incidence and predictors of sudden cardiac death after heart transplantation: A systematic review and meta-analysis. Clinical Transplantation 2018; 32(3): e13206.	Lack of basic information
187. Lin YN, Chang SS, Wang LM, Chi HT, Ueng KC. Prehospital predictors of initial shockable rhythm in out-of-hospital cardiac arrest: findings from the Taichung Sudden Unexpected Death Registry (THUNDER). Mayo Clinic Proceedings 2017; 92(3): 347–359.	Full text not available

188. Agbaedeng TA, Mahajan R, Munawar DA, et al. Risk of sudden cardiac death in obesity: a systematic review and meta-analysis of emerging evidence. European Heart Journal 2017;<br>38(suppl_1): ehx493.	Full text not available
189. Smith J, Brown L, Johnson P, Davis R. Obesity and sudden cardiac death: a systematic review and meta-analysis. European Heart Journal 2022; 38(suppl 1): ehx493.	Data or literature duplication
190. Agbaedeng T, Mahajan R, Munawar D, et al. Obesity associates with increased risk of sudden cardiac death: a systematic review and meta-analysis. Heart, Lung and Circulation 2017; 26(S1):<br>S186.	Full text not available
191. Ottaviani G, Buja LM. Anatomopathological changes of the cardiac conduction system in sudden cardiac death particularly in infants: advances over the last 25 years. Cardiovascular Pathology<br>2016; 25(6): 489–499.	Redundant information
192. Waite O, Smith A, Madge L, Spring H, Noret N. Sudden cardiac death in marathons: a systematic review. The Physician and Sportsmedicine 2016; 44(1):<br>79–84.	No relevant target data
193. Kong MH, Fonarow GC, Peterson ED, et al. Systematic review of the incidence of sudden cardiac death in the United States. Journal of the American College of Cardiology 2011; 57(7):<br>794–801.	No relevant target data
194. Vaartjes I, Hendrix A, Hertogh EM, et al. Sudden death in persons younger than 40 years of age: incidence and causes. European Journal of Cardiovascular Prevention & Rehabilitation 2009;<br>16(6): 592–596.	Unable to extract or use data
195. Bille K, Figueiras D, Schamasch P, et al. Sudden cardiac death in athletes: the Lausanne Recommendations. European Journal of Cardiovascular Prevention & Rehabilitation 2006; 13(6):<br>859–875.	Lack of basic information

Table S4. Basic information of the studies included by location.

	Number of studies, *n* (%)		Number of studies, *n* (%)
**Continent (N[a]=108)**		**Nation (N[b]=109)**	
		China	7 (6.42)
		India	1 (0.91)
		lran	1 (0.91)
Asia	19 (17.59)	Japan	3 (2.75)
		Korea	3 (2.75)
		Thailand	1 (0.91)
		Turkey	3 (2.75)
		Cameroon	1 (0.91)
Africa	4 (3.70)	Seychelles	1 (0.91)
		Tunisia	2 (1.83)
		Britain	8 (7.33)
		Czech Republic	1 (0.91)
		Denmark	3 (2.75)
		France	3 (2.75)
		Finland	3 (2.75)
		Germany	2 (1.83)
		Greece	2 (1.83)
Europe	42 (38.89)	Holland	2 (1.83)
		ltaly	2 (1.83)
		lreland	3 (2.75)
		Portugal	1 (0.91)
		Romania	1 (0.91)
		Spain	6 (5.50)
		Switzerland	3 (2.75)
		Swedish	2 (1.83)
Oceania	10 (9.26)	Australia	9 (8.26)
		New Zealand	2 (1.83)
North America	32 (29.62)	America	29 (26.61)
		Canada	3 (2.75)
South America	1 (0.92)	Brazil	1 (0.91)

Data were given as *n* (%) as indicated. N[a]=108, indicating that the total number of included studies was 108. N[b]=109, indicating that one of the included study data from Australia and New Zealand.

Table S5. Summary characteristics of the studies included in the systematic assessment.

	**Number of studies, *n* (%)**
National economic (N=108)	
High-income	89 (82.40)
Middle-income	18 (16.67)
Low-income	1 (0.92)
Year interval midpoint (N=108)	
1962-79	1 (0.93)
1980-89	3 (2.78)
1990-99	15 (13.89)
2000-09	67 (62.04)
2010-22	22 (20.37)
Study duration (N=108)	
0–4 years	25 (23.15)
5–9 years	24 (22.22)
10–14 years	23 (21.30)
15–19 years	12 (11.11)
20–24 years	13 (12.04)
25–29 years	8 (7.40)
≥30 years	3 (2.78)
Study design type (N=108)	
Case-control	17 (15.74)
Cohort	88 (81.48)
Cross sectional	3 (2.78%)
Study publication year (N=108)	
2003-07	19 (17.60)
2008-12	11 (10.19)
2013-17	46 (42.59)
2018-23	32 (29.63)

Data were given as *n* (%) as indicated. N=108, indicating that the total number of included studies was 108.

Table S6. Pooled incidence of sudden death by time period globally. The time periods are categorized based on the midpoint of each study period.

	Number of studies	Cases	Incidence (95% CI)
Global	68		16.93 (9.2, 26.97)
Period			
1971-1980	1	375	233.8 (210.74, 258.04)
1981-1990	1	1930	4.83 (4.61, 5.04)
1991-2000	9	339, 19	20.57 (0.00, 82.64)
2001-2010	42	461, 69	18.39 (8.41, 32.2)
2011-2020	15	546, 87	7.39 (2.19, 15.63)

Table S7. Integrated classification of countries, regions and super-regions in the analyzed areas.

Region	Country
Central Europe, eastern Europe, and central Asia	
Asia, central	Armenia, Azerbaijan, Georgia, Kazakhstan, Mongolia, Tajikistan, Turkmenistan, Uzbekistan
Europe, central	Albania, Bosnia and Herzegovina, Bulgaria, Croatia, Czech Republic, Hungary, Serbia and Montecnegro, Poland, Romania, Slovakia, Slovenia, TFYR Macedonia
Europe, eastern	Belarus, Estonia, Latvia, Lithuania, Moldova, Russia, Ukraine
High income	
Asia Pacific, high income	Brunei Darussalam, Japan, Republic of Korea, Singapore
Australasia	Australia, New Zealand
Europe, western	Austria, Belgium, Cyprus, Denmark, Finland, France, Germany, Greece, Iceland, Ireland, Israel, Italy, Luxembourg, Malta, Netherlands, Norway, Portugal, Spain, Sweden, Switzerland, United Kingdom
Latin America, southern	Argentina, Chile, Uruguay
North America, high income	Canada, United States of America, Greenland
Latin America and Caribbean	
Caribbean	Antigua and Barbuda, Bahamas, Barbados, Belize, Cuba, Dominican Republic, Grenada, Guyana, Haiti, Jamaica, Puerto Rico, Saint Lucia, Saint Vincent and the Grenadines, Suriname, Trinidad and Tobago, Virgin Island (US)
Latin America, Andean	Bolivia, Ecuador, Peru
Latin America, central	Colombia, Costa Rica, El Salvador, Guatemala, Honduras, Mexico, Nicaragua, Panama, Venezuela (Bolivarian Republic of)
Latin America, tropical	Brazil, Paraguay
North Africa and Middle East	
North Africa and the North Africa and the	Afghanistan, Algeria, Bahrain, Egypt, Iran (Islamic Republic of), Iraq, Jordan, Kuwait, Lebanon, Libyan Arab Jamahiriya, Morocco, Oman, Qatar, Saudi Arabia, Sudan, Syrian Arab Republic, Tunisia, Turkey, United Arab Emirates, Yemen
South Asia	
Asia, south	Bangladesh, Bhutan, India, Nepal, Pakistan
South East Asia, east Asia, and Oceania	
Asia, east	China, Dem. People’s Republic of Korea
Asia, South East	Cambodia, Indonesia, Lao People’s Democratic Republic, Malaysia, Maldives, Mauritius, Myanmar, Philippines, Seychelles, Sri Lanka, Thailand, Timor-Leste, Vietnam
Oceania	Fiji, Guam, Kiribati, Marshall Islands, Micronesia (Fed. States of), Papua New Guinea, Samoa, Solomon Islands, Tonga, Vanuatu
Sub-Saharan Africa	
Sub-Saharan Africa, central	Angola, Central African Republic, Congo, Democratic Republic of the Congo, Equatorial Guinea, Gabon
Sub-Saharan Africa, eastern	Burundi, Comoros, Djibouti, Eritrea, Ethiopia, Kenya, Madagascar, Malawi, Mozambique, Rwanda, Somalia, Uganda, United Republic of Tanzania, Zambia
Sub-Saharan Africa, southern	Botswana, Lesotho, Namibia, South Africa, Swaziland, Zimbabwe

Sub-Saharan Africa, western	Benin, Burkina Faso, Côte d'Ivoire, Cameroon, Cape Verde, Chad, Gambia, Ghana, Guinea, Guinea-Bissau, Liberia, Mali, Mauritania, Niger, Nigeria, São Tomé and Príncipe, Senegal, Sierra Leone, Togo

Table S8. Characteristics of studies reporting on incidence of sudden death.

Author	Year	Study time	Country	Region	Study design	Autopsy or not	Population size	Risk of bias	Reference
Morentin	2003	1991-1998	Spain	Europe, western	Cohort	Yes	551,636	Moderate	Sudden unexplained death among persons 1–35 years old.
Cho	2003	1999-2000	Republic of Korea	Asia Pacific, high income	Case control	Yes	5,325,000	Moderate	Arrhythmogenic right ventricular cardiomyopathy and sudden cardiac death in young Koreans.
Tabib	2003	1980-1999	France	Europe, western	Cohort	Yes	2,000,000	Moderate	Circumstances of death and gross and microscopic observations in a series of 200 cases of sudden death associated with arrhythmogenic right ventricular cardiomyopathy and/or dysplasia.
Doolan	2004	1994-2002	Australia	Australasia	Cohort	Yes	2,000,000	High	Causes of sudden cardiac death in young Australians.
Chugh,	2004	2002-2003	United States of America	North America, high income	Cohort	No	660,486	Low	Current burden of sudden cardiac death: multiple source surveillance versus retrospective death certificate-based review in a large U.S. community.
Eckart	2004	1977-2001	United States of America	North America, high income	Cohort	Yes	6,290,000	Low	Sudden death in young adults: A 25-year review of autopsies in military recruits.
Tümer	2005	1995-2000	Turkey	North Africa and the Middle East	Case control	Yes	NR	High	Sudden unexpected child deaths: Forensic autopsy results in cases of sudden deaths during a 5-year period.
Krous	2005	1979-2002	United States of America	North America, high income	Case control	No	NR	High	Sudden unexpected death in childhood: A report of 50 cases.
Tsuda	2005	1979-2003	Japan	Asia Pacific, high income	Cohort	No	NR	Moderate	Changes in causes of sudden deaths by decade in patients with coronary arterial lesions due to Kawasaki disease.
Puranik	2005	1995-2004	Australia	Australasia	Cohort	Yes	2,500,000	Moderate	Sudden death in the young.
Moore	2005	2003-2004	United Kingdom	Europe, western	Cohort	No	754,351	Low	Demographic and temporal trends in out of hospital sudden cardiac death in Belfast.
Fabre	2005	1994-2003	United Kingdom	Europe, western	Cohort	Yes	NR	Moderate	Sudden adult death syndrome and other non-ischaemic causes of sudden cardiac death.
Gerber	2006	1979-2002	United States of America	North America, high income	Cohort	No	124,277	Low	Seasonality and daily weather conditions in relation to myocardial infarction and sudden cardiac death in Olmsted County, Minnesota, 1979 to 2002.
Lee	2006	2001-2005	Republic of Korea	Asia Pacific, high income	Cohort	Yes	4,800,000	High	Causes of sudden death related to sexual activity: Results of a medicolegal postmortem study from 2001 to 2005.
Azmak	2007	1984-2005	Turkey	North Africa and the Middle East	Cohort	Yes	NR	Moderate	Sudden natural deaths in Edirne, Turkey, from 1984 to 2005.
Wang	2007	2003-2005	China	Asia, east	Cohort	No	NR	High	The epidemiology of non-traumatic prehospital sudden death in Macau.
Motozawa	2008	1997-2006	Japan	Asia Pacific, high	Case control	Yes	NR	Low	Sudden death while driving a four-wheeled vehicle: an autopsy analysis.

Author	Year	Study time	Country	Region	Study design	Autopsy or not	Population size	Risk of bias	Reference
				income					
Tavora	2007	2001-2006	United States of America	North America, high income	Case control	Yes	NR	Low	Discrepancies in initial death certificate diagnoses in sudden unexpected out-of-hospital deaths: the role of cardiovascular autopsy.
Byrne	2008	2005	Ireland	Europe, western	Cohort	No	414,277	Moderate	Multiple source surveillance incidence and aetiology of out-of-hospital sudden cardiac death in a rural population in the West of Ireland.
Morris	2009	2005	Ireland	Europe, western	Cohort	Yes	NR	Low	Sudden cardiac death in the young: a 1-year post-mortem analysis in the Republic of Ireland.
Sheikhazadi	2009	2001-2005	Iran (Islamic Republic of)	North Africa and the Middle East	Cohort	Yes	NR	Moderate	Survey of Sudden Death From Aneurysmal Subarachnoid Hemorrhage in Cadavers Referred to Legal Medicine Organization of Tehran, 2001-2005.
Adabag	2009	2001-2004	United States of America	North America, high income	Case control	Yes	NR	Moderate	Etiology of sudden death in the community: Results of anatomical, metabolic, and genetic evaluation.
Fragkouli	2010	1998-2008	Greece	Europe, western	Cohort	Yes	353,820	Moderate	Sudden cardiac death: An 11-year postmortem analysis in the region of Epirus, Greece.
Margey	2011	2005-2007	Ireland	Europe, western	Cohort	Yes	1,355,171	Moderate	Sudden cardiac death in 14-to 35-year olds in Ireland from 2005 to 2007: a retrospective registry.
Rao	2010	2010	India	Asia, south	Cohort	No	22,724	Moderate	Contribution of sudden cardiac death to total mortality in India - A population based study.
Morentin	2012	1991-2010	Spain	Europe, western	Cohort	Yes	474,665	Low	Clinicopathological features of sudden unexpected infectious death: Population-based study in children and young adults.
Havmoeller	2012	2002-2005	United States of America	North America, high income	Cohort	No	506,466	Moderate	Low Rate of Secondary Prevention ICDs in the General Population: Multiple-Year Multiple-Source Surveillance of Sudden Cardiac Death in the Oregon Sudden Unexpected Death Study.
Maron	2012	1986-2011	United States of America	North America, high income	Cohort	Yes	4,440,161	Moderate	Incidence of cardiovascular sudden deaths in Minnesota high school athletes.
Suárez-Mier	2013	1995-2010	Spain	Europe, western	Cross sectional	Yes	NR	Low	Pathology of sudden death during recreational sports in Spain.
Pilmer	2012	2008	Canada	North America, high income	Cohort	No	6,602,680	Moderate	Scope and nature of sudden cardiac death before age 40 in Ontario: A report from the Cardiac Death Advisory Committee of the Office of the Chief Coroner.
Nagata	2013	1962-2009	Japan	Asia Pacific, high income	Cohort	Yes	1,903	Low	Temporal trends in sudden unexpected death in a general population: The Hisayama Study.
Yang	2013	1996-2012	United States of America	North America, high income	Case control	Yes	1,058	Low	Sudden Cardiac Death Among Firefighters £45 Years of Age in the United States

Author	Year	Study time	Country	Region	Study design	Autopsy or not	Population size	Risk of bias	Reference
Pilmer	2013	2005-2009	Canada	North America, high income	Cohort	No	2,978,772	Low	Sudden cardiac death in children and adolescents between 1 and 19 years of age..
Winkel	2013	2000-2006	Denmark	Europe, western	Cohort	No	1,111,429	Low	Sudden cardiac death in children (1–18 years): symptoms and causes of death in a nationwide setting.
de Noronha	2013	2007-2009	United Kingdom	Europe, western	Cohort	No	NR	Moderate	The importance of specialist cardiac histopathological examination in the investigation of young sudden cardiac deaths.
Wang	2013	1978-2008	China	Asia, east	Cohort	Yes	NR	Moderate	The autopsy study of 553 cases of sudden cardiac death in Chinese adults.
Hofer	2014	2000-2007	Switzerland	Europe, western	Cohort	No	292,546	Low	Sudden cardiac death in the young (5-39 years) in the canton of Vaud, Switzerland.
Silva	2014	2007-2012	Portugal	Europe, western	Case control	Yes	NR	Moderate	Sudden Cardiac Death in Young Adult.
Martens	2014	2002-2009	Germany	Europe, western	Cohort	No	187,038	Low	Incidence of sudden cardiac death in Germany: results from an emergency medical service registry in Lower Saxony.
Zhang	2015	2006-2013	United States of America	North America, high income	Cohort	No	1,000,000	Low	Occupation and risk of sudden death in a United States community: a case–control analysis.
Feng	2015	1997-2010	Australia	Australasia	Cohort	No	NR	Low	Sudden cardiac death rates in an Australian population: a data linkage study
Niemeijer	2015	1990-2010	Netherlands	Europe, western	Cohort	No	137,944	Low	Declining incidence of sudden cardiac death from 1990–2010 in a general middle-aged and elderly population: The Rotterdam Study.
Braggion-Santos	2015	2006-2010	Brazil	Latin America, tropical	Cohort	Yes	600,000	Low	Sudden cardiac death in Brazil: a community-based autopsy series (2006-2010).
Zheng	2015	2007-2013	China	Asia, east	Case control	Yes	9,985,493	Low	A Case-Control Study of Sudden Unexplained Nocturnal Death Syndrome in the Southern Chinese Han Population.
Risgaard	2015	2000-2009	Denmark	Europe, western	Cohort	Yes	NR	Low	Risk factors and causes of sudden noncardiac death: A nationwide cohort study in Denmark.
Toukola	2015	1998-2007	Finland	Europe, western	Case control	Yes	NR	Low	Sudden cardiac death during physical exercise: Characteristics of victims and autopsy findings.
Naneix	2015	1985-2009	France	Europe, western	Case control	Yes	NR	Low	Sudden adult death: An autopsy series of 534 cases with gender andcontrol comparison.
Farioli	2015	1998-2012	United States of America	North America, high income	Cohort	No	300,000	Moderate	Incidence of sudden cardiac death in a young active population.
Harmon	2015	2003-2013	United States of	North America, high income	Cohort	No	4,242,519	Low	Incidence, Cause, and Comparative Frequency of Sudden Cardiac Death in National Collegiate Athletic Association Athletes: A Decade in Review.

Author	Year	Study time	Country	Region	Study design	Autopsy or not	Population size	Risk of bias	Reference
			America						
Chappex	2015	1995-2010	Switzerland	Europe, western	Case control	Yes	NR	Low	Sudden cardiac death among general population and sport related population in forensic experience.
Zhao	2016	1997-2012	Seychelles	Asia, South East	Cohort	Yes	92,000	Moderate	Sudden unexpected death from natural diseases: Fifteen years' experience with 484 cases in Seychelles.
Smallman	2016	2005-2010	United States of America	North America, high income	Cohort	No	1,383,110	Low	Sudden cardiac death associated with physical exertion in the US military, 2005-2010.
Wu	2016	1998-2013	China	Asia, east	Case control	Yes	NR	Low	Forensic Pathological Study of 1656 Cases of Sudden Cardiac Death in Southern China.
Bohm	2016	2012-2014	Germany	Europe, western	Cohort	No	NR	Low	Data from a nationwide registry on sports-related sudden cardiac deaths in Germany.
Maron	2016	2000-2014	United States of America	North America, high income	Cohort	No	1,308,730	Moderate	Comparison of the Frequency of Sudden Cardiovascular Deaths in Young Competitive Athletes Versus Nonathletes: Should We Really Screen Only Athletes?
Finocchiaro	2016	1994-2014	United Kingdom	Europe, western	Cohort	Yes	NR	High	Etiology of Sudden Death in Sports: Insights From a United Kingdom Regional Registry.
Vassalini	2016	1993-2012	Italy	Europe, western	Case control	Yes	NR	Moderate	An autopsy study of sudden cardiac death in persons aged 1-40 years in Brescia (Italy).
Parsons	2016	2001-2015	Australia	Australasia	Case control	Yes	NR	Moderate	Sudden cardiac death while playing Australian Rules football: a retrospective 14 year review.
Bagnall	2016	2010-2012	Australia	Australasia	Cohort	Yes	12,950,000	High	A Prospective Study of Sudden Cardiac Death among Children and Young Adults.
Bogle	2016	1948-2001	United States of America	North America, high income	Cohort	No	160,396	Low	Lifetime Risk for Sudden Cardiac Death in the Community.
Gräni	2016	1999-2010	Switzerland	Europe, western	Cohort	Yes	2,032,730	Moderate	Sports-related sudden cardiac death in Switzerland classified by static and dynamic components of exercise.
Kim	2016	2011-2013	United States of America	North America, high income	Cohort	No	725,225	Moderate	Sudden neurologic death masquerading as out-of-hospital sudden cardiac death.
Harmon	2016	2007-2013	United States of America	North America, high income	Cohort	No	6,974,640	Low	Incidence and Etiology of Sudden Cardiac Arrest and Death in High School Athletes in the United States.
Risgaard	2016	2000-2009	Denmark	Europe, western	Cohort	No	NR	Moderate	Sudden cardiac death: a nationwide cohort study among the young.
Ifteni	2017	2001-2015	Romania	Europe, central	Cohort	Yes	400,000	Moderate	Sudden Cardiac Death Autopsy Findings in 7200 Cases Between 2001 and 2015.
Krexi	2017	2004-2005	United	Europe, western	Cohort	Yes	NR	Moderate	Cardiovascular causes of maternal sudden death. Sudden Arrhythmic Death Syndrome

Author	Year	Study time	Country	Region	Study design	Autopsy or not	Population size	Risk of bias	Reference
			Kingdom						is leading cause in UK.
Feng	2016	1997-2010	Australia	Australasia	Cohort	No	13818413 (person-years)	Moderate	Temporal Trends in Sudden Cardiac Death From 1997 to 2010: A Data Linkage Study.
Wisten	2016	2000-2010	Sweden	Europe, western	Cohort	No	3,900,000	Moderate	Sudden cardiac death among the young in Sweden from 2000 to 2010: an autopsy-based study.
Bonny	2017	2013	Cameroon	Sub-Saharan Africa,western	Cohort	No	8298660 (person-years)	Low	Epidemiology of sudden cardiac death in Cameroon: the first population-based cohort survey in sub-Saharan Africa.
Ding	2017	2006-2015	China	Asia, east	Cohort	Yes	NR	High	Retrospective analysis of 769 cases of sudden cardiac death from 2006 to 2015: a forensic experience in China.
Finocchiaro	2018	1994-2014	United Kingdom	Europe, western	Cohort	Yes	NR	Moderate	Obesity and sudden cardiac death in the young: Clinical and pathological insights from a large national registry.
Anastasakis	2018	2002-2010	Greece	Europe, western	Cohort	Yes	1,500,000	Moderate	Sudden unexplained death in the young: epidemiology, aetiology and value of the clinically guided genetic screening.
Jayaraman	2017	2002-2015	United States of America	North America, high income	Cohort	No	1,000,000	Moderate	Risk Factors of Sudden Cardiac Death in the Young Multiple-Year Community-Wide Assessment.
Tseng	2018	2011-2014	United States of America	North America, high income	Cohort	No	805,235	Moderate	Prospective Countywide Surveillance and Autopsy Characterization of Sudden Cardiac Death POST SCD Study.
Drezner	2018	2014-2016	United States of America	North America, high income	Cohort	No	NR	Moderate	Survival After Exercise-Related Sudden Cardiac Arrest in Young Athletes: Can We Do Better?
Ripoll	2019	2015-2017	Spain	Europe, western	Cohort	Yes	1,115,999	Moderate	Post-mortem toxicology in the diagnosis of sudden death in young and middle-aged victims.
Zhang	2019	2015	China	Asia, east	Cohort	No	3,224,103	Low	Epidemiological investigation of sudden cardiac death in multiethnic Xinjiang Uyghur autonomous region in Northwest China.
Haukilahti	2019	1998-2017	Finland	Europe, western	Cohort	Yes	NR	Moderate	Sudden Cardiac Death in Women Causes of Death, Autopsy Findings, and Electrocardiographic Risk Markers.
Shuvy	2019	2003-2014	Canada	North America, high income	Cohort	No	73,984,929	Moderate	Temporal trends in sudden cardiac death in Ontario, Canada.
Brodie	2019	2012-2013	Australia	Australasia	Cross sectional	No	NR	Moderate	Sudden natural death behind the wheel: Review of driver deaths and fitness to drive assessment history in Victoria, Australia 2012-2013.
Srettabunjong	2018	2002-2010	Thailand	Asia, South East	Cohort	Yes	NR	Moderate	Sudden Unexplained Nocturnal Death Syndrome: Epidemiological and Morphological Characteristics in Thai Autopsy Cases.
Morentin	2019	1991-2016	Spain	Europe, western	Cohort	Yes	268,545	Moderate	Sudden cardiac death associated to substances of abuse and psychotropic drugs consumed by young people: A population study based on forensic autopsies.

Author	Year	Study time	Country	Region	Study design	Autopsy or not	Population size	Risk of bias	Reference
Joodi	2019	2013-2015	United States of America	North America, high income	Case control	No	4,589	Low	Coronary Artery Disease and Atherosclerotic Risk Factors in a Population-Based Study of Sudden Death.
Sheppard	2023	1994-2021	United Kingdom	Europe, western	Cohort	Yes	NR	High	Sudden arrhythmic death and cardiomyopathy are important causes of sudden cardiac death in the UK: results from a national coronial autopsy database.
Belhaj	2023	2010-2019	Tunisia	North Africa and the Middle East	Cohort	Yes	11,100,000	Moderate	Risk factors of sudden cardiac death in women: A 10 years study in Tunisia.
Puolitaival	2023	1998-2017	Finland	Europe, western	Cohort	Yes	NR	Moderate	Causes and characteristics of unexpected sudden cardiac death in octogenarians/nonagenarians.
Finocchiaro	2023	1994-2022	United Kingdom	Europe, western	Cohort	No	NR	Moderate	Sudden Cardiac Death Among Adolescents in the United Kingdom.
Stattin	2022	2000-2010	Sweden	Europe, western	Case control	No	15,633	Low	Cohort profile: the Swedish study of SUDden cardiac Death in the Young (SUDDY) 2000-2010: a complete nationwide cohort of SCDs.
Frontera	2022	2010-2015	Italy	Europe, western	Cohort	Yes	21,100,000	Low	Antemortem characterization of sudden deaths as first-manifestation in Italy.
Rücklová	2022	2014-2019	Czech Republic	Europe, central	Cohort	Yes	1,675,552	Moderate	BURDEN OF SUDDEN CARDIAC DEATH IN PERSONS AGED 1-40 YEARS IN THE CZECH REPUBLIC.
Colantonio	2021	2003-2013	United States of America	North America, high income	Cohort	No	30,239	Low	Higher Serum Urate Levels Are Associated With an Increased Risk for Sudden Cardiac Death.
Işın	2021	2011-2019	Turkey	North Africa and the Middle East	Cohort	Yes	NR	Moderate	Epidemiology of Football-Related Sudden Cardiac Death in Turkey.
Guo	2021	1995-2015	United States of America	North America, high income	Cross sectional	Yes	NR	Low	Genetic Variants Associated With Unexplained Sudden Cardiac Death in Adult White and African American Individuals.
Ripoll-Vera	2021	2015-2019	Spain	Europe, western	Cohort	Yes	1,144,392	Moderate	Sudden cardiac death in persons aged 50 years or younger: diagnostic yield of a regional molecular autopsy program using massive sequencing.
Saadi	2020	1990-2018	Tunisia	North Africa and the Middle East	Cohort	Yes	NR	Moderate	Sudden death in the young adult: a Tunisian autopsy-based series.
Ha	2020	2000-2016	Australia	Australasia	Cohort	No	21,437,580	Moderate	Sudden Cardiac Death in the Young Incidence, Trends, and Risk Factors in a Nationwide Study.
Chen	2020	2000-2016	China	Asia, east	Cohort	No	46,490	Low	Incidence and risk factors of sudden cardiac death in bipolar disorder across the lifespan.
Park	2020	2002-2015	Republic of Korea	Asia Pacific, high income	Cohort	No	410,119	Low	Short stature is associated with incident sudden cardiac death in a large Asian cohort.
Chang	2008	1989-2004	United States of	North America, high income	Cohort	No	8,850,468	Moderate	Sudden Infant Death Syndrome: Changing Epidemiologic Patterns in California 1989-2004.

Author	Year	Study time	Country	Region	Study design	Autopsy or not	Population size	Risk of bias	Reference
			America						
Liebrechts-Akkerman	2013	1984-2005	Netherlands	Europe, western	Cohort	No	NR	Moderate	Histological Findings in Unclassified Sudden Infant Death, Including Sudden Infant Death Syndrome.
Malloy, M. H.	2013	2005-2007	United States of America	North America, high income	Cohort	Yes	11,944,444	High	Prematurity and sudden infant death syndrome: United States 2005–2007.
Evans	2013	2000-2010	Australia	Australasia	Cohort	Yes	NR	Moderate	Postmortem review and genetic analysis in sudden infant death syndrome: an 11-year review.
Hakeem	2015	1995-2004	United States of America	North America, high income	Cohort	No	37,418,280	Low	Incidence and determinants of sudden infant death syndrome: a population-based study on 37 million births.
Tuchtan	2018	2000-2017	France	Europe, western	Cohort	No	60,000	Moderate	Sudden unexpected infant death characteristics in the French region of West Provence-Alpes-Cote d'Azur.
Drake	2019	2004-2013	United States of America	North America, high income	Cohort	No	689,365	Low	A Descriptive and Geospatial Analysis of Environmental Factors Attributing to Sudden Unexpected Infant Death.
Mitchell	2023	2012-2018	New Zealand	Australasia	Cohort	No	415,401	Low	Regional variation in sudden unexpected death in infancy in New Zealand.
Bartick	2022	2015-2018	United States of America	North America, high income	Cohort	No	13,092,035	Moderate	The Role of Breastfeeding in Racial and Ethnic Disparities in Sudden Unexpected Infant Death: A Population-Based Study of 13 Million Infants From the United States.
Anderson	2021	2006-2015	United States of America	North America, high income	Cohort	No	NR	Moderate	Circadian variation in sudden unexpected infant death in the United States.

Abbreviation: NR, not reported.

Table S9. Details information on the incidence of sudden death in children, adults, and the overall population of the studies.

	Age of population, years	Sudden deaths	Incidence per 100,000 person-years (95% CI)		
			Total population	Male population	Female population
Children					
Barry et al (2012), United States of America					
1986-2011	12-18	17	0.30 (0.20, 0.50)[1]	0.50 (0.30, 0.90)[1]	0
Winkel et al (2013), Denmark					
2000-2006	1-18	87	1.47 (1.21, 1.77)[1,3]	NA	NA
Harmonl et al (2016), United States of America					
2007-2013	14-18	69	0.99 (0.78, 1.26)[1,3]	1.45 (1.12, 1.89) [1,3]	0.32 (0.15, 0.62)[1,3]
Adult					
Eckart et al (2004), United States of America					
1977-2001	17-35	126	13.00 (10.90, 15.50)[1]	13.30 (11.00, 16.00)[1]	11.50 (7.00, 18.90)[1]
1977-1981	...	37	14.60 (10.60, 20.10)[1]	NA	NA
1982-1986	...	37	15.70 (11.40, 21.70)[1]	NA	NA
1987-1991	...	20	10.20 (6.60, 15.80)[1]	NA	NA
1992-1996	...	13	9.10 (5.30, 15.50)[1]	NA	NA
1997-2001	...	19	13.60 (8.70, 21.20)[1]	NA	NA
Lee et al (2006), Republic of Korea					
2001-2005	32-64	14	0.06 (0.03, 0.10)[2,3]	NA	NA
Zhang et al (2015), United States of America					
2006-2013	18-65	646	8.67 (8.05, 9.35)[2,3]	NA	NA
Niemeijer et al (2015), Netherlands					
1990-2010	≥ 45	583	422.64 (389.41, 458.65)[1,3]	513.43 (455.87, 578.1)[1,3]	363.61 (324.47, 407.39)[1,3]
Farioli et al (2015), United States of America					
1998-2012	18-64	182	18.10 (15.70, 21.00)[1,5]	18.10 (15.70, 21.00)[1,5]	NA
1998	...	15	27.50 (13.40, 41.70) [1,5]	27.50 (13.40, 41.70) [1,5]	NA
1999		10	20.00 (7.50, 32.60) [1,5]	20.00 (7.50, 32.60) [1,5]	NA
2000	...	12	21.50 (9.30, 33.70) [1,5]	21.50 (9.30, 33.70) [1,5]	NA
2001	...	12	19.90 (8.60, 31.10) [1,5]	19.90 (8.60, 31.10) [1,5]	NA
2002	...	13	23.60 (10.60, 36.60) [1,5]	23.60 (10.60, 36.60) [1,5]	NA
2003	...	16	24.80 (12.60, 36.90) [1,5]	24.80 (12.60, 36.90) [1,5]	NA
2004	...	13	20.00 (9.00, 31.10) [1,5]	20.00 (9.00, 31.10) [1,5]	NA
2005	...	12	19.80 (8.60, 31.00) [1,5]	19.80 (8.60, 31.00) [1,5]	NA
2006	...	11	16.70 (6.80, 26.60) [1,5]	16.70 (6.80, 26.60) [1,5]	NA
2007	...	15	21.10 (10.40, 31.90) [1,5]	21.10 (10.40, 31.90) [1,5]	NA
2008	...	7	9.00 (2.30, 15.70) [1,5]	9.00 (2.30, 15.70) [1,5]	NA

2009	...	10	13.00 (4.90, 21.20) [1,5]	13.00 (4.90, 21.20) [1,5]	NA
2010	...	12	15.90 (6.90, 25.00) [1,5]	15.90 (6.90, 25.00) [1,5]	NA
2011	...	12	17.30 (7.50, 27.10) [1,5]	17.30 (7.50, 27.10) [1,5]	NA
2012	...	12	15.0 (6.40, 23.60) [1,5]	15.0 (6.40, 23.60) [1,5]	NA
Farioli et al (2016), United States of America					
1948-2001	≧28	375	233.80 (211.03, 258.98)[2,3]	NA	NA
Kim et al (2016), United States of America					
2011-2013	≥18	336	15.44 (13.86, 17.21)[2,3]	21 (18.43, 23.92)[2,3]	9.66 (7.92, 11.77)[2,3]
Feng et al (2016), Australia					
1997-2010	35-84	8077	58.45 (57.19, 59.74)[2,3]	82.67 (80.54, 84.85)[2,3]	34.44 (33.08, 35.86)[2,3]
Bonny et al (2017), Cameroon					
2013	≥18	135	1.63 (1.37, 1.92)[1]	1.87 (1.57, 2.21)[1]	0.30 (0.51, 1.00)[1]
Kim et al (2018), United States of America					
2011-2014	18-90	630	29.6[1,4]	NA	NA
Shuvy et al (2019), Canada					
2003-2014	35-74	36,334	49.11 (48.61, 49.62)[2,3]	75.27 (74.38, 76.17)[2,3]	23.98 (23.49, 24.48)[2,3]
2003	...	3578	57.07 (55.22, 58.98) [2,3]	86.29 (83.08, 89.61) [2,3]	27.85 (26.05, 29.77) [2,3]
2004	...	3493	54.55 (52.76, 56.4) [2,3]	82.04 (78.95, 85.26) [2,3]	27.06 (25.3, 28.94) [2,3]
2005	...	3236	49.55 (47.86, 51.29) [2,3]	74.22 (71.31, 77.25) [2,3]	24.87 (23.21, 26.66) [2,3]
2006	...	3294	50.25 (48.56, 52) [2,3]	75.18 (72.25, 78.22) [2,3]	25.33 (23.65, 27.12) [2,3]
2007	...	3332	50.75 (49.05, 52.51) [2,3]	78.4 (75.39, 81.54) [2,3]	24.18 (22.56, 25.92) [2,3]
2008	...	3289	49.46 (47.79, 51.19) [2,3]	77.02 (74.05, 80.1) [2,3]	22.98 (21.41, 24.67) [2,3]
2009	...	3179	46.97 (45.36, 48.64) [2,3]	70.55 (67.73, 73.48) [2,3]	24.31 (22.71, 26.03) [2,3]
2010	...	3258	47.34 (45.73, 49) [2,3]	73.03 (70.19, 75.99) [2,3]	22.65 (21.11, 24.29) [2,3]
2011	...	3107	44.35 (42.81, 45.94) [2,3]	67.51 (64.81, 70.33) [2,3]	22.09 (20.58, 23.7) [2,3]
2012	...	3208	45.04 (43.5, 46.63) [2,3]	68.47 (65.77, 71.29) [2,3]	22.52 (21.01, 24.13) [2,3]
2013	...	3360	46.47 (44.92, 48.08) [2,3]	71.04 (68.3, 73.88) [2,3]	22.87 (21.37, 24.48) [2,3]
Kim et al (2021), United States of America					
2003-2013		235	155.43 (136.49, 176.94)[2,3]	NA	NA
Park et al (2020), Republic of Korea					
2002-2015	≥20	197	39.86 (37.77, 42.06)[1,3]	47.54 (44.39, 50.92)[1,3]	31.59 (28.94, 34.48)[1,3]
Overall					
Morentin et al (2003), Spain					
1991-1998	1-35	107	2.42 (2.00, 2.94)[2,3]	NA	NA
Tabib et al (2003), France					
1980-1999	1-65	200	4.82 (4.61, 5.05)[2,3]	NA	NA
Doolan et al (2004), Australia					
1994-2002	0-35	239	9.65 (8.36, 11.14)[2,3]	NA	NA

Chugh et al (2004), United States of America					
2002-2003	NA	353	53.45 (48.08, 59.4)[2,3]	NA	NA
Puranik et al (2005), Australia					
1995-2004	5-35	427	1.71 (1.55, 1.88)[2,3]	NA	NA
Moore et al (2005), United Kingdom					
2003-2004	NA	300	19.88 (17.73, 22.30)[2,3]	NA	NA
Gerber et al (2006), United States of America					
1979-2002	NA	2066	69.27 (66.33, 72.34)[2,3]	NA	NA
Wang et al (2007), China					
2003-2005	12-101	605	46.00[1,4]	NA	NA
Byrne et al (2008), Ireland					
2005	0-80+	212	51.17 (44.62, 58.67)[1,3]	76.42 (65.24, 89.47)[1,3]	25.38 (19.14, 33.55)[1,3]
Morris et al (2009), Ireland					
2005	<35	69	3.18[1,4]	4.98[1,4]	1.77[1,4]
Fragkouli et al (2010), Greece					
1998-2008	1-80	688	32.22 (30.47, 34.07)[2,3]	NA	NA
Margey et al (2011), Ireland					
2005-2007	< 35	116	2.85 (2.37, 3.44)[1,3]	4.36 (3.53, 5.39)[1,3]	1.30 (0.87, 1.93)[1,3]
Rao et al (2010), India					
2010	<= 90	173	761.31 (654.28, 885.3)[2,3]	NA	NA
Morentin et al (2012), Spain					
1991-2010	0-34	339	3.57 (3.21, 3.98)[2,3]	4.74 (4.16, 5.41)[2,3]	2.34 (1.92, 2.83)[2,3]
Havmoeller et al (2012), United States of America					
2002-2005	0-85+	1175	58.00 (54.75, 61.44)[1,3]	70.00 (65.00, 75.37)[1,3]	45.00 (41.03, 49.35)[1,3]
2002	...	NA	NA	60.00	42.8
2003	...	NA	NA	69.18	37.93
2004	...	NA	NA	82.05	54.76
Maron et al (2012), Canada					
2008	2-40	174	2.64 (2.26, 3.07)[1,3]	NA	NA
Pilmer et al (2013), Canada					
2005-2009	1-19	116	0.78 (0.64, 0.93)[1]	1.01 (0.8, 1.27)[1,3]	0.54 (0.39, 0.75)[1,3]
Hofer et al (2014), Switzerland					
2000-2007	5-39	40	1.71[1,4]	2.73[1,4]	0.69[1,4]
Martens et al (2014), Germany					
2002-2009	0-90+	1212	81.00 (76.54, 85.72)[1,3]	55 (51.37, 58.89)[1,3]	25 (22.56, 27.70) [1,3]
2002	...	151	79[1,4]	54[1,4]	25[1,4]
2003	...	149	78[1,4]	51[1,4]	27[1,4]
2004	...	152	80[1,4]	57[1,4]	23[1,4]

2005	...	153	81[1,4]	58[1,4]	23[1,4]
2006	...	154	81[1,4]	55[1,4]	26[1,4]
2007	...	151	79[1,4]	56[1,4]	24[1,4]
2008	...	150	79[1,4]	52[1,4]	27[1,4]
2009	...	152	80[1,4]	58[1,4]	22[1,4]
Feng et al (2015), Australia					
1997-2010	≥ 1	9567	37.80[1,4]	NA	NA
Braggion-Santos et al (2015), Brazil					
2006-2010	10-80	899	29.97 (28.05, 32.01)[1,3]	NA	NA
Zheng et al (2015), China					
2007-2013	0-111	879	1.26 (1.18, 1.34)[1]	14.83[1,4]	1.26[1,4]
Risgaard et al (2015), Denmark					
2000-2009	1-49	1039	3.80 (3.60, 4.10)[1]	NA	NA
Harmon et al (2015), United States of America					
2003-2013	17-24	79	1.86 (1.48, 2.33)[1]	2.65 (2.05, 3.40)[1]	0.82 (0.48, 1.39)[1]
Zhao et al (2016), Seychelles					
1997-2012	0-97	484	32.88 (30.05, 35.98)[2,3]	NA	NA
Smallman et al (2016), United States of America					
2005-2010	NA	200	1.63 (1.37, 1.92)[1]	1.87 (1.57, 2.21)[1]	0.30 (0.51, 1.00)[1]
Bohm et al (2016), Germany					
2012-2014	10-79	144	0.15[1,4]	NA	NA
Maron et al (2016), United States of America					
2000-2014	14-23	27	2.06 (1.39, 3.05)[2,3]	NA	NA
Bagnall et al (2016), Australia					
2010-2012	1-35	490	1.30 (1.20, 1.40)[1,3]	1.80 (1.62, 2.00)[1,3]	0.70 (0.59, 0.83)[1,3]
Gräni et al (2016), Switzerland					
1999-2010	10-39	69	0.59 (0.50, 0.70)[1,5]	NA	NA
Risgaard et al (2016), Denmark					
2000-2009	1-49	1575	8.60 (8.00, 9.20)[1]	NA	NA
Ifteni et al (2017), Romania					
2001-2015	0-89	1085	18.08 (17.03, 19.20)[2,3]	NA	NA
Wisten et al (2016), Sweden					
2000-2010	1-35	552	1.29 (1.18, 1.40)[1,3]	NA	NA
2000	...	NA	NA	2.24[4,6]	1.10[4,6]
2001	...	NA	NA	1.41[4,6]	1.01[4,6]
2002	...	NA	NA	1.37[4,6]	0.79[4,6]
2003	...	NA	NA	2.32[4,6]	0.91[4,6]
2004	...	NA	NA	1.82[4,6]	0.85[4,6]

2005	...	NA	NA	1.07[4,6]	0.84[4,6]
2006	...	NA	NA	1.72[4,6]	0.48[4,6]
2007	...	NA	NA	1.50[4,6]	0.26[4,6]
2008	...	NA	NA	1.64[4,6]	0.67[4,6]
2009	...	NA	NA	1.62[4,6]	0.47[4,6]
2010	...	NA	NA	1.80[4,6]	0.25[4,6]
Anastasakis et al (2018), Greece					
2002-2010	1-35	349	2.74 (2.46, 3.04)[1]	NA	NA
Jayaraman et al (2017), United States of America					
2002-2015	5-34	3775	26.96 (26.11, 27.84)[2,3]	NA	NA
Ripoll et al (2019), Spain					
2015-2017	16-50	101	3.02 (2.47, 3.68)[2,3]	NA	NA
Zhang et al (2019), China					
2015	NA	1244	38.58 (36.48, 40.8)[1,3]	49.30 (46.80, 51.70)[1,5]	28.10 (26.30, 30.00)[1,5]
Morentin et al (2019), Spain					
1991-2016	15-35	204	2.92 (2.54, 3.36)[2,3]	NA	NA
Belhaj et al (2023), Tunisia					
2010-2019	NA	2385	2.15 (2.06, 2.24)[2,3]	NA	NA
Frontera et al (2022), Italy					
2010-2015	1-35	301	0.24 (0.13, 0.45)[1]	0.40[1,4]	0.10[1,4]
Rücklová et al (2022), Czech Republic					
2014-2019	1-40	245	2.44 (2.15, 2.77)[1,3]	3.20[1,4]	0.80[1,4]
Işınet al (2021), Turkey					
2011-2019	10-59	118	0.41[1,4]	NA	NA
Ripoll-Vera et al (2021), Spain					
2015-2019	0-50	123	5.80[1,4]	10.10[1,4]	1.60[1,4]
Ha et al (2020), Australia					
2000-2016	1-35	2006	0.62 (0.60, 0.65)[1,3]	NA	NA
2001	...	119	0.61 (0.51, 0.74)[1,3]	NA	NA
2002	...	107	0.54 (0.45, 0.66)[1,3]	NA	NA
2003	...	137	0.69 (0.58, 0.82)[1,3]	NA	NA
2004	...	109	0.54 (0.45, 0.66)[1,3]	NA	NA
2005	...	114	0.56 (0.46, 0.67)[1,3]	NA	NA
2006	...	141	0.68 (0.58, 0.81)[1,3]	NA	NA
2007	...	121	0.58 (0.48, 0.7)[1,3]	NA	NA
2008	...	149	0.70 (0.60, 0.83)[1,3]	NA	NA
2009	...	145	0.67 (0.57, 0.79)[1,3]	NA	NA
2010	...	133	0.60 (0.51, 0.72)[1,3]	NA	NA

2011	...	138	0.62 (0.52, 0.73)[1,3]	NA	NA
2012	...	122	0.54 (0.45, 0.64)[1,3]	NA	NA
2013	...	130	0.56 (0.47, 0.67)[1,3]	NA	NA
2014	...	102	0.43 (0.36, 0.53)[1,3]	NA	NA
2015	...	101	0.42 (0.35, 0.52)[1,3]	NA	NA
Chen et al (2020), China					
2000-2016	NA	467	132.86 (121.22, 145.61)[1]	165.62 (147.43, 186.02)[1]	99.91 (85.90, 116.15)[1]

Table S10. Studies reporting on the incidence of sudden death in overall population.

Author	Year	Study time	Country	Population size	Autopsy or not	Population	Population density 2020 (persons/km²)	Incidence (95%CI) (per 100,000 person-years)	Incidence (95%CI) of male (per 100,000 person-years)	Incidence (95%CI) of female (per 100,000 person-years)
Morentin	2003	1991-1998	Spain	551,636	Yes	Overall	90.26	2.42 (2.00, 2.94)[2,3]	NR	NR
Tabib	2003	1980-1999	France	2,000,000	Yes	Overall	119.14	4.82 (4.61, 5.05)[2,3]	NR	NR
Doolan	2004	1994-2002	Australia	2,000,000	Yes	Overall	3.49	9.65 (8.36, 11.14)[2,3]	NR	NR
Chugh	2004	2002-2003	United States of America	660,486	No	Overall	30.17	53.45 (48.08, 59.4)[2,3]	NR	NR
Eckart	2004	1977-2001	United States of America	6,290,000	Yes	Adult	30.17	13.00 (10.90, 15.50)[1]	13.30 (11.00, 16.00)[1]	11.50 (7.00, 18.90)[1]
Puranik	2005	1995-2004	Australia	2,500,000	Yes	Overall	3.49	1.71 (1.55, 1.88)[2,3]	NR	NR
Moore	2005	2003-2004	United Kingdom	754,351	No	Overall	269.85	19.88 (17.73, 22.30)[2,3]	NR	NR
Gerber	2006	1979-2002	United States of America	124,277	No	Overall	30.17	69.27 (66.33, 72.34)[2,3]	NR	NR
Lee	2006	2001-2005	Republic of Korea	4,800,000	Yes	Adult	505.42	0.06 (0.03, 0.10)[2,3]	NR	NR
Wang	2007	2003-2005	China	NR	No	Overall	146.47	46.00[1,4]	NR	NR
Byrne	2008	2005	Ireland	414,277	No	Overall	68.45	51.17 (44.62, 58.67)[1,3]	76.42 (65.24, 89.47)[1,3]	25.38 (19.14, 33.55)[1,3]
Morris	2009	2005	Ireland	NR	Yes	Overall	68.45	3.18[1,4]	4.98[1,4]	1.77[1,4]
Fragkouli	2010	1998-2008	Greece	353,820	Yes	Overall	79.12	32.22 (30.47, 34.07)[2,3]	NR	NR
Margey	2011	2005-2007	Ireland	1,355,171	Yes	Overall	68.45	2.85 (2.37, 3.44)[1,3]	4.36 (3.53, 5.39)[1,3]	1.30 (0.87, 1.93)[1,3]
Rao	2010	2010	India	22,724	No	Overall	445.77	761.31 (654.28, 885.30)[2,3]	NR	NR
Morentin,	2012	1991-2010	Spain	474,665	Yes	Overall	90.26	3.57 (3.21, 3.98)[2,3]	4.74 (4.16, 5.41)[2,3]	2.34 (1.92, 2.83)[2,3]
Havmoeller	2012	2002-2005	United States of America	506,466	No	Overall	30.17	58.00 (54.75, 61.44)[1,3]	70.00 (65.00, 75.37)[1,3]	45.00 (41.03, 49.35)[1,3]
Barry	2012	1986-2011	United States of America	4,440,161	Yes	Children	30.17	0.30 (0.20, 0.50)[1]	0.50 (0.30, 0.90)[1]	NR
Maron	2012	2008	Canada	6,602,680	No	Overall	2.69	2.64 (2.26, 3.07)[1,3]	NR	NR
Pilmer	2013	2005-2009	Canada	2,978,772	No	Overall	2.69	0.78 (0.64, 0.93)[1]	1.01 (0.8, 1.27)[1,3]	0.54 (0.39, 0.75)[1,3]
Winkel	2013	2000-2006	Denmark	1,111,429	No	Children	129.26	1.47 (1.21, 1.77)[1,3]	NR	NR
Hofer	2014	2000-2007	Switzerland	292,546	No	Overall	214.45	1.71[1,4]	2.73[1,4]	0.69[1,4]
Martens	2014	2002-2009	Germany	187,038	No	Overall	236.32	81.00 (76.54, 85.72)[1,3]	55.00 (51.37, 58.89)[1,3]	25.00 (22.56, 27.70)[1,3]
Zhang	2015	2006-2013	United States of America	1,000,000	No	Adult	30.17	8.67 (8.05, 9.35)[2,3]	NR	NR
Feng	2015	1997-2010	Australia	NR	No	Overall	3.49	37.80 [1,4]	NR	NR
Niemeijer	2015	1990-2010	Netherlands	137,944	No	Adult	496.64	422.64 (389.41, 458.65)[1,3]	513.43 (455.87, 578.1)[1,3]	363.61 (324.47, 407.39)[1,3]
Braggion-Santos	2015	2006-2010	Brazil	600,000	Yes	Overall	25.02	29.97 (28.05, 32.01)[1,3]	NR	NR
Zheng	2015	2007-2013	China	9,985,493	Yes	Overall	146.47	1.26 (1.18, 1.34)	14.83[1,4]	1.26[1,4]
Risgaard	2015	2000-2009	Denmark	NR	Yes	Overall	129.26	3.80 (3.60, 4.10)[1]	NR	NR
Farioli	2015	1998-2012	United States of America	300,000	No	Adult	30.17	18.10 (15.70, 21.00)[1,5]	18.10 (15.70, 21.00)[1,5]	NR
Harmon	2015	2003-2013	United States of America	4,242,519	No	Overall	30.17	1.86 (1.48, 2.33)[1]	2.65 (2.05, 3.40)[1]	0.82 (0.48, 1.39)[1]

Author	Year	Study time	Country	Population size	Autopsy or not	Population	Population density 2020 (persons/km²)	Incidence (95%CI) (per 100,000 person-years)	Incidence (95%CI) of male (per 100,000 person-years)	Incidence (95%CI) of female (per 100,000 person-years)
Zhao	2016	1997-2012	Seychelles	92,000	Yes	Overall	534.61	32.88 (30.05, 35.98)[2,3]	NR	NR
Smallman	2016	2005-2010	United States of America	1,383,110	No	Overall	30.17	1.63 (1.37, 1.92)[1]	1.87 (1.57, 2.21)[1]	0.30 (0.51, 1.00)[1]
Bohm	2016	2012-2014	Germany	NR	No	Overall	236.32	0.15[1,4]	NR	NR
Maron	2016	2000-2014	United States of America	1,308,730	No	Overall	30.17	2.06 (1.39, 3.05)[2,3]	NR	NR
Bagnall	2016	2010-2012	Australia	12,950,000	Yes	Overall	3.49	1.30 (1.20, 1.40)[1,3]	1.80 (1.62, 2.00)[1,3]	0.70 (0.59, 0.83)[1,3]
Bogle	2016	1948-2001	United States of America	160,396	No	Adult	30.17	233.80 (211.03, 258.98)[2,3]	NR	NR
Gräni	2016	1999-2010	Switzerland	2,032,730	Yes	Overall	214.45	0.59 (0.50, 0.70)[1,5]	NR	NR
Kim	2016	2011-2013	United States of America	725,225	No	Adult	30.17	15.44 (13.86, 17.21)[2,3]	21 (18.43, 23.92)[2,3]	9.66 (7.92, 11.77)[2,3]
Harmon	2016	2007-2013	United States of America	6,974,640	No	Children	30.17	0.99 (0.78, 1.26)[1,3]	1.45 (1.12, 1.89)[1,3]	0.32 (0.15, 0.62)[1,3]
Risgaard	2016	2000-2009	Denmark	NR	No	Overall	129.26	8.60 (8.00, 9.20)[1]	NR	NR
Ifteni	2017	2001-2015	Romania	400,000	Yes	Overall	80.78	18.08 (17.03, 19.20)[2,3]	NR	NR
Feng	2016	1997-2010	Australia	13,818,413 (person-years)	No	Adult	3.49	58.45 (57.19, 59.74)[2,3]	82.67 (80.54, 84.85)[2,3]	34.44 (33.08, 35.86)[2,3]
Wisten	2016	2000-2010	Sweden	3,900,000	No	Overall	18.96	1.29 (1.18, 1.40)[1,3]	NR	NR
Bonny	2017	2013	Cameroon	8,298,660 (person-years)	No	Adult	56.51	1.63 (1.37, 1.92)[1]	1.87 (1.57, 2.21)[1]	0.30 (0.51, 1.00)[1]
Anastasakis	2018	2002-2010	Greece	1,500,000	Yes	Overall	79.12	2.74 (2.46, 3.04)[1]	NR	NR
Jayaraman	2017	2002-2015	United States of America	1,000,000	No	Overall	30.17	26.96 (26.11, 27.84)[2,3]	NR	NR
Tseng	2018	2011-2014	United States of America	805,235	No	Adult	30.17	29.6[1,4]	NR	NR
Ripoll	2019	2015-2017	Spain	1,115,999	Yes	Overall	90.26	3.02 (2.47, 3.68)[2,3]	NR	NR
Zhang	2019	2015	China	3,224,103	No	Overall	146.47	38.58 (36.48, 40.8)[1,3]	49.30 (46.80, 51.70)[1,5]	28.10 (26.30, 30.00)[1,5]
Shuvy	2019	2003-2014	Canada	73,984,929	No	Adult	2.69	49.11 (48.61, 49.62)[2,3]	75.27 (74.38, 76.17)[2,3]	23.98 (23.49, 24.48)[2,3]
Morentin	2019	1991-2016	Spain	268,545	Yes	Overall	90.26	2.92 (2.54, 3.36)	NR	NR
Belhaj	2023	2010-2019	Tunisia	11,100,000	Yes	Overall	75.64	2.15 (2.06, 2.24)[2,3]	NR	NR
Frontera	2022	2010-2015	Italy	21,100,000	Yes	Overall	198.98	0.24 (0.13, 0.45)[1]	0.40[1,4]	0.10[1,4]
Rücklová	2022	2014-2019	Czech Republic	1,675,552	Yes	Overall	139.42	2.44 (2.15, 2.77)[1,3]	3.20[1,4]	0.80[1,4]
Colantonio	2021	2003-2013	United States of America	30,239	No	Adult	30.17	155.43 (136.49, 176.94)[2,3]	NR	NR
Işın	2021	2011-2019	Turkey	NR	Yes	Overall	100.75	0.41[1,4]	NR	NR
Ripoll-Vera	2021	2015-2019	Spain	1,144,392	Yes	Overall	90.26	5.8[1,4]	10.10[1,4]	1.60[1,4]
Ha	2020	2000-2016	Australia	21,437,580	No	Overall	3.49	0.62 (0.60, 0.65) [1,3]	NR	NR
Chen	2020	2000-2016	China	46,490	No	Overall	146.47	132.86 (121.22, 145.61) [1]	165.62 (147.43, 186.02) [1]	99.91 (85.90, 116.15) [1]

**Author**	**Year**	**Study time**	**Country**	**Population size**	**Autopsy or not**	**Population**	**Population density 2020 (persons/km²)**	**Incidence (95%CI) (per 100,000 person-years)**	**Incidence (95%CI) of male (per 100,000 person-years)**	**Incidence (95%CI) of female (per 100,000 person-years)**
Park	2020	2002-2015	Republic of Korea	410,119	No	Adult	505.42	39.86 (37.77, 42.06)[1,3]	47.54 (44.39, 50.92)[1,3]	31.59 (28.94, 34.48)[1,3]
Chang	2008	1989-2004	United States of America	8,850,468	No	Infant	30.17	71.22 (69.48, 73.00)[2,3]	NR	NR
Malloy	2013	2005-2007	United States of America	11,944,444	Yes	Infant	30.17	43.56 (42.39, 44.76)[2,3]	50.88 (49.11, 52.7)[2,3]	35.88 (34.37, 37.46)[2,3]
Hakeem	2015	1995-2004	United States of America	37,418,280	No	Infant	30.17	64.45 (63.64, 65.27)[2,3]	74.90 (73.68, 76.14)[2,3]	53.55 (52.5, 54.63)[2,3]
Tuchtan	2018	2000-2017	France	60,000	No	Infant	119.14	12.75 (10.69, 15.19)[2,3]	NR	NR
Drake	2019	2004-2013	United States of America	689,365	No	Infant	30.17	106.18 (98.7, 114.23)[1,3]	119.47 (108.46, 131.58)[1,3]	92.29 (82.45, 103.28)[1,3]
Mitchell	2023	2012-2018	New Zealand	415,401	No	Infant	16.86	63.87 (56.54, 72.12)[2,3]	NR	NR
Bartick	2022	2015-2018	United States of America	13,092,035	No	Infant	30.17	91.31 (89.69, 92.97)[2,3]	NR	NR

Abbreviation: CI, confidence interval.

[a] Popoulation：Infant (0-1 years)、Children (2-17 years)、Adult (≥18 years)、(Overall).

[b] Rates Calculation: person-years.

[c] Prevalence was presented as the number of cases per 100,000 person-years.

[d] *** Notes: 1 Value reported from the study; 2 Rate checked and confirmed; 3 Confidence Interval (CI) estimated and added by the authors as it was not present in the original study; 4 It was not possible to calculate the confidence interval due to lack of raw data; 5 Age and/or sex adjusted. NR= Not Reported.

[e] Autopsy or not: Whether all subjects included in the study had undergone autopsies.

Table S11. Estimated incidence of sudden death (per 100,000 person-years) by country, region, and super-region.

Country	Region	Super-region	Estimated incidence (per 100,000 person-years)	Incidence log transformed (95% CI)	Number of people with sudden death (per thousand)	Missing data	Estimate borrowed from	UN population 2021 (per thousand)
Armenia			13.80	2.62 (0.60, 4.93)	220.729915	Yes	Super-region	2870.35
Azerbaijan			13.88	2.63 (0.62, 4.92)	95.179641	Yes	Super-region	10234.37
Georgia			10.40	2.34 (0.27, 4.70)	2964.84097	Yes	Super-region	3788.45
Kazakhstan			31.53	3.45 (1.31, 5.83)	7012.92672	Yes	Super-region	19743.6
Mongolia			36.69	3.60 (1.47, 6.00)	1197.939629	Yes	Super-region	3339.67
Tajikistan			13.44	2.60 (0.54, 4.94)	1034.565258	Yes	Super-region	9966.91
Turkmenistan			62.06	4.13 (1.99, 6.49)	6513.329536	Yes	Super-region	7092.04
Uzbekistan			13.80	2.62 (0.60, 4.93)	220.729915	Yes	Super-region	34243.7
	Asia, central		14.12	2.65 (0.57, 5.01)		Yes	Super-region	
Albania			27.46	3.31 (1.30, 5.49)	713.264892	Yes	Region	2849.64
Bosnia and Herzegovina			113.67	4.73 (2.69, 6.93)	836.862289	Yes	Region	3244.91
Bulgaria			16.99	2.83 (0.79, 5.04)	1686.984519	Yes	Region	6877.23
Croatia			10.52	2.35 (0.31, 4.55)	943.868705	Yes	Region	3924.61
Czech Republic			21.33	3.06 (0.99, 5.17)	2583.175804	No		10530.68
Hungary			22.77	3.13 (1.11, 5.29)	2358.036765	Yes	Region	9707.85
Poland			23.19	3.14 (1.14, 5.30)	8878.60602	Yes	Region	38040.3
Romania			28.22	3.34 (1.21, 5.58)	5166.206144	No		19248.16
Serbia and Montenegro			15.06	2.71 (0.68, 4.91)	1824.855384	Yes	Region	7439.28
Slovakia			26.63	3.28 (1.27, 5.44)	1335.109028	Yes	Region	5442.76
Slovenia			44.91	3.80 (1.79, 5.97)	539.573997	Yes	Region	2113.49
TFYR Macedonia			18.37	2.91 (0.88, 5.10)	436.306627	Yes	Region	1851.11
	Europe, central		22.68	3.12 (1.10, 5.31)				

Country	Region	Super-region	Estimated incidence (per 100,000 person-years)	Incidence log transformed (95% CI)	Number of people with sudden death (per thousand)	Missing data	Estimate borrowed from	UN population 2021 (per thousand)
Belarus			21.47	3.07 (0.98, 5.45)	3454.461558	Yes	Super-region	9251.37
Estonia			8.94	2.19 (0.06, 4.55)	307.63425	Yes	Super-region	1331.75
Latvia			24.42	3.20 (1.05, 5.55)	2661.887403	Yes	Super-region	1885.59
Lithuania			18.37	2.91 (0.81, 5.28)	437.058236	Yes	Super-region	2794.49
Moldova			9.47	2.25 (0.25, 4.54)	317.194522	Yes	Super-region	3023.78
Russia			32.05	3.47 (1.33, 5.85)	27869.293998	Yes	Super-region	145836.18
Ukraine			28.4	3.35 (1.29, 5.68)	2963.579016	Yes	Super-region	44298.64
	Europe, eastern		21.81	3.08 (1.00, 5.47)		Yes	Super-region	
		Central Europe, eastern Europe, and central Asia	20.28	3.01 (0.95, 5.34)				
Brunei Darussalam			4.68	1.54 (-1.35, 2.83)	48.59	Yes	Region	451.72
Japan			4.18	1.43 (-1.07, 3.22)	13333.54	Yes	Region	125679.34
Republic of Korea			3.55	1.27 (-1.08, 3.40)	4586.97	No		51848.4
Singapore			2.82	1.04 (-30.87, 35.89)	616.68	Yes	Region	5546.29
	Asia Pacific, high income		5.15	1.64 (-6.99, 11.04)				
Australia			7.66	2.04 (0.88, 3.18)	3818.18	No		25956.42
New Zealand			8.46	2.14 (0.58, 3.76)	748.43	No		5107.7
	Australasia		8.38	2.13 (0.82, 3.31)				
Austria			6.51	1.87 (0.97, 2.75)	1177.71	Yes	Region	8967.06
Belgium			17.86	2.88 (1.37, 4.49)	1547.80	Yes	Region	11570.84
Cyprus			3.82	1.34 (0.47, 2.21)	174.31	Yes	Region	1317.31

Country	Region	Super-region	Estimated incidence (per 100,000 person-years)	Incidence log transformed (95% CI)	Number of people with sudden death (per thousand)	Missing data	Estimate borrowed from	UN population 2021 (per thousand)
Denmark			10.04	2.31 (1.19, 3.57)	871.488864	No		5856.78
Finland			2.85	1.05 (-0.08, 2.05)	768.547796	Yes	Region	5541.08
France			8.72	2.17 (0.79, 3.49)	9258.305355	No		66083.55
Germany			7.60	2.03 (0.62, 3.20)	10830.402152	No		83697.08
Greece			12.38	2.52 (1.35, 4.32)	1793.23542	No		10579.56
Iceland			31.01	3.43 (2.26, 4.48)	52.206864	Yes	Region	372.64
Ireland			11.49	2.44 (1.30, 3.90)	810.582916	No		5028.43
Israel			11.74	2.46 (0.90, 4.13)	1240.383004	Yes	Region	8942.92
Italy			7.46	2.01 (0.14, 3.29)	7496.033425	No		59729.35
Luxembourg			16.50	2.80 (1.78, 3.88)	91.55861	Yes	Region	640.27
Malta			8.79	2.17 (-3.20, 8.11)	74.19977	Yes	Region	524.38
Netherlands			12.72	2.54 (0.48, 5.06)	2858.166272	No		17730.56
Norway			5.22	1.65 (0.53, 2.66)	757.673409	Yes	Region	5408.09
Portugal			16.29	2.79 (1.89, 3.67)	1317.573728	Yes	Region	10390.96
Spain			11.17	2.41 (1.28, 3.75)	7465.857224	No		47735.66
Sweden			7.67	2.04 (0.11, 3.30)	1361.388191	No		10416.13
Switzerland			7.17	1.97 (0.37, 3.22)	1050.066612	No		8707.02
United Kingdom			9.89	2.29 (0.92, 3.94)	9872.876461	No		67668.79
	Europe, western		9.34	2.23 (1.30, 3.23)				
Argentina			12.47	2.52 (1.19, 3.67)	26802.21362	Yes	Super-region	45312.28
Chile			18.54	2.92 (1.60, 4.06)	12718.602921	Yes	Super-region	19456.33
Uruguay			9.5	2.25 (0.93, 3.39)	99.183348	Yes	Super-region	3396.69
	Latin America,		10.51	2.35 (1.03, 3.49)		Yes	Super-region	

Country	Region	Super-region	Estimated incidence (per 100,000 person-years)	Incidence log transformed (95% CI)	Number of people with sudden death (per thousand)	Missing data	Estimate borrowed from	UN population 2021 (per thousand)
	southern							
Canada			6.95	1.94 (0.40, 3.11)	4875.974808	No		38454.06
Greenland			12.75	2.55 (1.38, 3.62)	7.851204	Yes	Region	56.04
United States of America			10.86	2.39 (1.48, 3.31)	57079.089632	No		340161.44
	North America, high income		8.72	2.17 (1.01, 3.24)				
		High income	8.68	2.16 (0.36, 4.05)				
Antigua and Barbuda			38.06	3.64 (1.43, 6.62)	10.278555	Yes	Super-region	92.35
Bahamas			15.94	2.77 (0.55, 5.89)	69.206202	Yes	Super-region	396.37
Barbados			19.37	2.96 (-0.60, 6.56)	191.946645	Yes	Super-region	282.15
Belize			26.76	3.29 (1.05, 6.42)	123.309665	Yes	Super-region	395.35
Cuba			6.85	1.92 (-0.28, 4.99)	1368.02691	Yes	Super-region	11122.17
Dominican Republic			14.29	2.66 (0.42, 5.65)	6195.77836	Yes	Super-region	11123.48
Grenada			89.15	4.49 (2.16, 7.50)	68.333664	Yes	Super-region	116.69
Guyana			157.45	5.06 (2.78, 8.20)	83.831344	Yes	Super-region	815.48
Haiti			30.29	3.41 (0.69, 6.54)	568.7295	Yes	Super-region	11374.59
Jamaica			29.94	3.40 (1.07, 6.40)	1746.875808	Yes	Super-region	2837.68
Puerto Rico			13.18	2.58 (0.17, 5.58)	994.506297	Yes	Super-region	3253.21
Saint Lucia			17.8	2.88 (0.53, 5.88)	13.317592	Yes	Super-region	178.52
Saint Vincent and the Grenadines			116.3	4.76 (2.46, 7.74)	26.522436	Yes	Super-region	102.84
Suriname			23.42	3.15 (0.88, 6.29)	529.10777	Yes	Super-region	617.9

Country	Region	Super-region	Estimated incidence (per 100,000 person-years)	Incidence log transformed (95% CI)	Number of people with sudden death (per thousand)	Missing data	Estimate borrowed from	UN population 2021 (per thousand)
Trinidad and Tobago			38.61	3.65 (1.34, 6.65)	361.367188	Yes	Super-region	1487.72
Virgin Island (US)			24.88	3.21 (0.98, 6.20)	37.836036	Yes	Super-region	87.22
	Caribbean		30.25	3.41 (1.17, 6.40)		Yes	Super-region	
Bolivia			16.55	2.81 (0.55, 5.94)	1215.223248	Yes	Super-region	11937.36
Ecuador			16.61	2.81 (0.60, 5.88)	4745.96958	Yes	Super-region	17682.45
Peru			13.79	2.62 (0.41, 5.75)	18467.82516	Yes	Super-region	33155.88
	Latin America, Andean		31.04	3.44 (1.24, 6.53)		Yes	Super-region	
Colombia			21.09	3.05 (0.84, 6.13)	21765.209884	Yes	Super-region	51188.17
Costa Rica			9.88	2.29 (0.09, 5.36)	6528.399098	Yes	Super-region	5059.99
El Salvador			15.58	2.75 (0.32, 5.76)	1308.083598	Yes	Super-region	6255.78
Guatemala			83.31	4.42 (2.22, 7.43)	48552.915485	Yes	Super-region	17598.65
Honduras			18.76	2.93 (0.74, 5.99)	1609.337232	Yes	Super-region	10289.88
Mexico			21.11	3.05 (0.84, 6.12)	15241.18911	Yes	Super-region	127648.15
Nicaragua			20.76	3.03 (0.83, 6.12)	7453.404858	Yes	Super-region	6644.74
Panama			28.19	3.34 (1.14, 6.42)	1065.929073	Yes	Super-region	4345.41
Venezuela (Bolivarian Republic of)			31.14	3.44 (1.24, 6.55)	7579.033572	Yes	Super-region	28237.83
	Latin America, central		26.59	3.28 (1.08, 6.34)		Yes	Super-region	
Brazil			49.69	3.91 (1.57, 6.60)	73677.881964	No		209550.29
Paraguay			24.96	3.22 (0.87, 6.15)	2105.5167	Yes	Region	6684.18
	Latin America,		38.25	3.64 (1.31, 6.57)				

Country	Region	Super-region	Estimated incidence (per 100,000 person-years)	Incidence log transformed (95% CI)	Number of people with sudden death (per thousand)	Missing data	Estimate borrowed from	UN population 2021 (per thousand)
	tropical							
		Latin America and Caribbean	28.77	3.36 (1.16, 6.39)				
Afghanistan			3.53	1.26 (-0.94, 3.32)	5776.059204	Yes	Region	40000.41
Algeria			16.09	2.78 (0.57, 4.83)	6333.69565	Yes	Region	44761.1
Bahrain			5.28	1.66 (-8.87, 13.09)	208.294112	Yes	Region	1501.76
Egypt			6.52	1.88 (-0.28, 3.90)	16188.627759	Yes	Region	110957.01
Iran (Islamic Republic of)			19.50	2.97 (0.76, 5.02)	12392.614149	Yes	Region	88455.49
Iraq			19.06	2.95 (0.77, 4.98)	6408.996048	Yes	Region	43071.21
Jordan			6.20	1.83 (-0.35, 3.84)	1489.532056	Yes	Region	11066.36
Kuwait			3.15	1.15 (-1.22, 3.28)	636.231966	Yes	Region	4360.74
Lebanon			6.39	1.86 (-1.79, 5.43)	809.11398	Yes	Region	5718.12
Libyan Arab Jamahiriya			3.13	1.14 (-1.08, 3.21)	951.119494	Yes	Region	7135.18
Morocco			15.03	2.71 (0.53, 4.75)	5077.540056	Yes	Region	36954.44
Oman			8.74	2.17 (-0.05, 4.22)	669.662496	Yes	Region	4500.42
Qatar			8.94	2.19 (-0.17, 4.32)	398.31967	Yes	Region	2814.98
Saudi Arabia			26.33	3.27 (1.06, 5.32)	4479.95691	Yes	Region	31328.37
Sudan			6.13	1.81 (-0.40, 3.86)	8256.47127	Yes	Region	58932.7
Syrian Arab Republic			14.48	2.67 (0.50, 4.70)	2941.52224	Yes	Region	21628.84
Tunisia			7.90	2.07 (-0.14, 4.25)	1757.893658	No		12048.62
Turkey			6.34	1.85 (-0.40, 3.97)	11667.96925	No		86686.25
United Arab Emirates			10.01	2.30 (0.12, 4.31)	1371.445905	Yes	Region	9789.05

Country	Region	Super-region	Estimated incidence (per 100,000 person-years)	Incidence log transformed (95% CI)	Number of people with sudden death (per thousand)	Missing data	Estimate borrowed from	UN population 2021 (per thousand)
Yemen			3.92	1.37 (-0.82, 3.42)	5051.07128	Yes	Region	37140.23
	North Africa and the Middle East		8.02	2.08 (-0.25, 4.17)				
		North Africa and Middle East	10.17	2.32 (-0.19, 4.41)				
Bangladesh			49.38	3.90 (-1.08, 9.07)	82001.943535	Yes	Region	167658.85
Bhutan			45.58	3.82 (1.00, 7.83)	386.94456	Yes	Region	775.44
India			133.20	4.89 (2.08, 7.76)	795631.11414	No		1414203.90
Nepal			87.53	4.47 (1.95, 8.03)	14416.227391	Yes	Region	29475.01
Pakistan			106.29	4.67 (2.15, 8.13)	120696.8112	Yes	Region	239477.80
	Asia, south		90.67	4.51 (1.85, 7.74)				
		South Asia	55.14	4.01 (1.40, 7.51)				
China			33.70	3.52 (2.16, 4.95)	454804.65774	No		1458174.60
Dem. People's Republic of Korea			26.81	3.29 (1.14, 5.58)	7859.265988	Yes	Region	26232.53
	Asia, east		32.01	3.47 (1.75, 5.37)				
Cambodia			55.41	4.01 (2.09, 6.23)	5189.046567	Yes	Region	16974.31
Indonesia			17.80	2.88 (1.00, 5.07)	82916.71178	Yes	Region	276758.05
Lao People's Democratic Republic			23.59	3.16 (1.12, 5.48)	2468.496528	Yes	Region	7453.19
Malaysia			28.88	3.36 (1.45, 5.57)	11241.19896	Yes	Region	34282.40
Maldives			32.98	3.50 (-0.18, 7.52)	162.58725	Yes	Region	516.15
Mauritius			74.16	4.31 (1.63, 7.28)	399.113478	Yes	Region	1279.62
Myanmar			50.04	3.91 (1.99, 6.14)	17505.63009	Yes	Region	53387.10

Country	Region	Super-region	Estimated incidence (per 100,000 person-years)	Incidence log transformed (95% CI)	Number of people with sudden death (per thousand)	Missing data	Estimate borrowed from	UN population 2021 (per thousand)
Philippines			67.07	4.21 (2.02, 6.70)	34925.57336	Yes	Region	113100.95
Seychelles			47.66	3.86 (1.83, 6.18)	41.140155	No		122.99
SriLanka			12.57	2.53 (0.55, 4.86)	7295.898918	Yes	Region	22700.37
Thailand			95.99	4.56 (2.68, 6.77)	23756.091696	Yes	Region	71727.33
Timor-Leste			57.80	4.06 (2.13, 6.28)	400.586538	Yes	Region	1350.14
Vietnam			93.22	4.53 (2.63, 6.84)	31481.14882	Yes	Region	98935.10
	Asia, South East		37.22	3.62 (1.76, 5.92)				
Fiji			21.07	3.05 (1.24, 5.12)	306.639495	Yes	Super-region	916.71
Guam			17.79	2.88 (1.09, 4.85)	65.557492	Yes	Super-region	163.73
Kiribati			26.17	3.26 (1.60, 5.21)	17.986038	Yes	Super-region	128.38
Marshall Islands			34.65	3.55 (1.83, 5.52)	102.245955	Yes	Super-region	41.37
Micronesia (Fed. States of)			36.86	3.61 (1.92, 5.57)	330.831851	Yes	Super-region	521.57
Papua New Guinea			25.84	3.25 (1.39, 5.36)	339.43731	Yes	Super-region	10012.90
Samoa			48.37	3.88 (2.12, 5.92)	120.272628	Yes	Super-region	213.78
Solomon Islands			34.85	3.55 (1.71, 5.63)	159.457569	Yes	Super-region	762.59
Tonga			41.14	3.72 (2.01, 5.70)	19.958708	Yes	Super-region	105.49
Vanuatu			16.46	2.80 (0.95, 4.89)	29.027063	Yes	Super-region	305.87
	Oceania		25.34	3.23 (1.51, 5.23)		Yes	Super-region	
		South East Asia, east Asia, and Oceania	29.23	3.38 (1.69, 5.29)				
Angola			1.22	0.20 (-3.31, 2.46)	1111.944246	Yes	Super-region	34532.43

Country	Region	Super-region	Estimated incidence (per 100,000 person-years)	Incidence log transformed (95% CI)	Number of people with sudden death (per thousand)	Missing data	Estimate borrowed from	UN population 2021 (per thousand)
Central African Republic			5.75	1.75 (-1.75, 4.01)	901.77444	Yes	Super-region	5112.10
Congo			4.16	1.42 (-2.05, 3.68)	367.082814	Yes	Super-region	5892.18
Democratic Republic of the Congo			4.29	1.46 (-2.07, 3.69)	2339.914748	Yes	Super-region	99148.93
Equatorial Guinea			3.11	1.13 (-2.45, 3.34)	650.224663	Yes	Super-region	1758.79
Gabon			3.94	1.37 (-2.12, 3.63)	121.21272	Yes	Super-region	2376.72
	Sub-Saharan Africa, central		4.82	1.57 (-1.93, 3.83)		Yes	Super-region	
Burundi			3.77	1.33 (-3.34, 4.45)	325.433548	Yes	Super-region	12965.48
Comoros			3.68	1.30 (-3.57, 4.76)	302.477449	Yes	Super-region	818.17
Djibouti			15.56	2.74 (-0.82, 4.96)	422.823375	Yes	Super-region	1121.25
Eritrea			2.80	1.03 (-2.49, 3.28)	327.647604	Yes	Super-region	3350.18
Ethiopia			4.25	1.45 (-2.17, 3.63)	25539.179169	Yes	Super-region	122138.59
Kenya			23.74	3.17 (-0.45, 5.35)	4049.978837	Yes	Super-region	53219.17
Madagascar			9.40	2.24 (-1.32, 4.46)	596.790708	Yes	Super-region	29691.08
Malawi			8.93	2.19 (-1.66, 4.41)	158.373354	Yes	Super-region	20047.26
Mozambique			24.02	3.18 (-0.36, 5.41)	5111.29736	Yes	Super-region	31707.80
Rwanda			5.85	1.77 (-3.06, 5.14)	849.394536	Yes	Super-region	13355.26
Somalia			2.68	0.98 (-2.52, 3.24)	2868.784523	Yes	Super-region	17271.43
Uganda			7.52	2.02 (-1.88, 4.25)	9990.218368	Yes	Super-region	45910.93
United Republic of Tanzania			4.66	1.54 (-2.06, 3.72)	5133.244497	Yes	Super-region	62830.41
Zambia			3.28	1.19 (-2.32, 3.45)	5992.823577	Yes	Super-region	19603.61

Country	Region	Super-region	Estimated incidence (per 100,000 person-years)	Incidence log transformed (95% CI)	Number of people with sudden death (per thousand)	Missing data	Estimate borrowed from	UN population 2021 (per thousand)
	Sub-Saharan Africa, eastern		5.61	1.72 (-2.08, 3.92)		Yes	Super-region	
Botswana			6.32	1.84 (-1.65, 4.11)	37.702608	Yes	Super-region	2401.44
Lesotho			5.32	1.67 (-1.93, 3.85)	292.643276	Yes	Super-region	2261.54
Namibia			4.21	1.44 (-2.06, 3.70)	182.404695	Yes	Super-region	2810.55
South Africa			9.23	2.22 (-1.34, 4.44)	15394.10078	Yes	Super-region	61502.60
Swaziland			1.64	0.49 (-3.11, 2.68)	50.918098	Yes	Super-region	1206.59
Zimbabwe			4.51	1.51 (-2.02, 3.74)	1559.184627	Yes	Super-region	15797.21
	Sub-Saharan Africa, southern		5.33	1.67 (-1.86, 3.91)		Yes	Super-region	
Benin			2.59	0.95 (-2.47, 3.36)	1311.832476	Yes	Region	13413.42
Burkina Faso			4.98	1.61 (-1.81, 4.01)	2307.300676	Yes	Region	21995.24
Cameroon			2.55	0.94 (-2.22, 3.51)	2454.717312	No		26915.76
Cape Verde			1.87	0.63 (-2.86, 3.03)	52.59497	Yes	Region	516.65
Chad			1.94	0.66 (-2.65, 3.10)	1707.948266	Yes	Region	17828.27
Côte d'Ivoire			1.40	0.33 (-3.09, 2.74)	2783.171586	Yes	Region	29639.74
Gambia			1.81	0.59 (-3.14, 3.14)	241.887339	Yes	Region	2576.01
Ghana			3.31	1.20 (-2.29, 3.61)	3180.325926	Yes	Region	32518.67
Guinea			1.68	0.52 (-2.84, 2.94)	1380.648357	Yes	Region	13710.51
Guinea-Bissau			5.62	1.73 (-1.63, 4.14)	213.707592	Yes	Region	2058.84
Liberia			3.25	1.18 (-2.18, 3.59)	529.613524	Yes	Region	5259.32
Mali			1.63	0.49 (-2.83, 2.93)	2102.292357	Yes	Region	22388.63
Mauritania			11.27	2.42 (-0.88, 4.87)	482.009766	Yes	Region	4734.87
Niger			2.09	0.74 (-2.59, 3.17)	2494.317852	Yes	Region	24502.14

Country	Region	Super-region	Estimated incidence (per 100,000 person-years)	Incidence log transformed (95% CI)	Number of people with sudden death (per thousand)	Missing data	Estimate borrowed from	UN population 2021 (per thousand)
Nigeria			4.21	1.44 (-2.27, 4.00)	20935.105982	Yes	Region	218529.29
São Tomé and Príncipe			3.31	1.20 (-2.57, 3.79)	21.485728	Yes	Region	221.96
Senegal			3.88	1.35 (-2.07, 3.76)	1634.260563	Yes	Region	17220.87
Sierra Leone			1.35	0.30 (-3.15, 2.71)	832.12488	Yes	Region	8094.60
Togo			3.42	1.23 (-2.28, 3.68)	842.558262	Yes	Region	8878.38
	Sub-Saharan Africa, western		3.64	1.29 (-2.14, 3.71)				
		Sub-Saharan Africa	6.01	1.79 (-1.83, 3.98)				

Abbreviation: CI, confidence interval.

Table S12. Incidence trends of SIDS in included studies.

Study	Age of population, years	Sudden death	Incidence rate per 100, 000 person-years (95% CI)
Chang et al. (2008), United States of America			
1989-2004	<1	6303	71.22 (69.48, 73.00)[2,3]
1989	...	NA	137.57[4,6]
1990	...	NA	118.34[4,6]
1991	...	NA	120.08[4,6]
1992	...	NA	95.51[4,6]
1993	...	NA	94.04[4,6]
1994	...	NA	86.36[4,6]
1995	...	NA	72.70[4,6]
1996	...	NA	57.97[4,6]
1997	...	NA	63.34[4,6]
1998	...	NA	50.75[4,6]
1999	...	NA	46.71[4,6]
2000	...	NA	42.67[4,6]
2001	...	NA	37.78[4,6]
2002	...	NA	32.25[4,6]
2003	...	NA	33.13[4,6]
2004	...	NA	32.08[4,6]
Malloy et al. (2013), United States of America			
2005-2007	<1	5203	43.56 (42.39, 44.76)[2,3]
Hakeem et al. (2015), United States of America			
1995-2004	<1	24101	64.45 (63.64, 65.27)[2,3]
1995	...	NA	82.84[4,6]
1996	...	NA	77.11[4,6]
1997	...	NA	73.00[4,6]
1998	...	NA	68.25[4,6]
1999	...	NA	63.16[4,6]
2000	...	NA	59.55[4,6]
2001	...	NA	54.30[4,6]
2002	...	NA	53.96[4,6]
2003	...	NA	52.14[4,6]
2004	...	NA	55.07[4,6]
Tuchtan et al. (2018), France			
2000-2017	<1	130	12.75 (10.69, 15.19)[2,3]
Drake et al. (2019), United States of America			
2004-2013	<1	732	106.18 (98.7, 114.23)[1,3]
2004	...	70	104.27 (81.89, 132.54)[2,3]
2005	...	86	128.42 (103.34, 159.37)[2,3]
2006	...	57	81.23 (62.08, 106.02)[2,3]
2007	...	92	128.87 (104.47, 158.78)[2,3]

2008	...	104	145.24 (119.27, 176.7)[2,3]
2009	...	98	135.49 (110.58, 165.84)[2,3]
2010	...	68	99.76 (78.06, 127.25)[2,3]
2011	...	55	83.39 (63.42, 109.37)[2,3]
2012	...	65	96.51 (75.08, 123.8)[2,3]
2013	...	37	54.18 (38.7, 75.51)[2,3]
Mitchell et al. (2023), New Zealand			
2012-2018	<1	267	63.87 (56.54, 72.12)[2,3]
Bartick et al. (2022), United States of America			
2015-2018	<1	11942	91.31 (89.69, 92.97)[2,3]

[1] Value reported from the study; [2] Rate checked and confirmed; [3] Confidence Interval (CI) estimated and added by the authors as it was not present in the original study; [4] It was not possible to calculate the confidence interval due to lack of raw data; [5] Age and/or sex adjusted; [6] Data were extracted from the figures and tables of the papers. NA=not available.

Table S13. The articles included in the systematic review.

NO.	Atricle
1	Morentin B, Suárez-Mier MP, Aguilera B. Sudden unexplained death among persons 1-35 years old. Forensic Sci Int 2003; 135(3):213-7.
2	Cho Y, Park T, Yang DH, et al. Arrhythmogenic right ventricular cardiomyopathy and sudden cardiac death in young Koreans. Circ J 2003; 67(11):925-8.
3	Tabib A, Loire R, Chalabreysse L, et al. Circumstances of death and gross and microscopic observations in a series of 200 cases of sudden death associated with arrhythmogenic right ventricular cardiomyopathy and/or dysplasia. Circulation 2003; 108(24):3000-5.
4	Doolan A, Langlois N, Semsarian C. Causes of sudden cardiac death in young Australians. Med J Aust 2004; 180(3):110-2.
5	Chugh SS, Jui J, Gunson K, et al. Current burden of sudden cardiac death: multiple source surveillance versus retrospective death certificate-based review in a large U.S. community. J Am Coll Cardiol 2004; 44(6):1268-75.
6	Eckart RE, Scoville SL, Campbell CL, et al. Sudden death in young adults: a 25-year review of autopsies in military recruits. Ann Intern Med 2004; 141(11):829-34.
7	Tümer AR, Tümer L, Bilge Y. Sudden unexpected child deaths: forensic autopsy results in cases of sudden deaths during a 5-year period. J Trop Pediatr 2005; 51(3):131-5.
8	Krous HF, Chadwick AE, Crandall L, Nadeau-Manning JM. Sudden unexpected death in childhood: a report of 50 cases. Pediatr Dev Pathol 2005; 8(3):307-19.
9	Tsuda E, Arakaki Y, Shimizu T, et al. Changes in causes of sudden deaths by decade in patients with coronary arterial lesions due to Kawasaki disease. Cardiol Young 2005; 15(5):481-8.
10	Puranik R, Chow CK, Duflou JA, Kilborn MJ, McGuire MA. Sudden death

	in the young. Heart Rhythm 2005; 2(12): 1277-82.
11	Fabre A, Sheppard MN. Sudden adult death syndrome and other non-ischaemic causes of sudden cardiac death. Heart 2006; 92(3):316-20.
12	Moore MJ, Glover BM, McCann CJ, et al. Demographic and temporal trends in out of hospital sudden cardiac death in Belfast. Heart 2006; 92(3):311-5.
13	Gerber Y, Jacobsen SJ, Killian JM, Weston SA, Roger VL. Seasonality and daily weather conditions in relation to myocardial infarction and sudden cardiac death in Olmsted County, Minnesota, 1979 to 2002. J Am Coll Cardiol 2006; 48(2):287-92.
14	Lee S, Chae J, Cho Y. Causes of sudden death related to sexual activity: results of a medicolegal postmortem study from 2001 to 2005. J Korean Med Sci 2006; 21(6):995-9.
15	Azmak AD. Sudden natural deaths in Edirne, Turkey, from 1984 to 2005. Med Sci Law 2007; 47(2):147-55.
16	Wang H, Lei W, Li Y, Xu T. The epidemiology of non-traumatic prehospital sudden death in Macau. Resuscitation 2007; 74(2):222-6.
17	Motozawa Y, Hitosugi M, Kido M, Kurosu A, Nagai T, Tokudome S. Sudden death while driving a four-wheeled vehicle: an autopsy analysis. Med Sci Law 2008; 48(1):64-8.
18	Tavora F, Crowder C, Kutys R, Burke A. Discrepancies in initial death certificate diagnoses in sudden unexpected out-of-hospital deaths: the role of cardiovascular autopsy. Cardiovasc Pathol 2008; 17(3):178-82.
19	Byrne R, Constant O, Smyth Y, et al. Multiple source surveillance incidence and aetiology of out-of-hospital sudden cardiac death in a rural population in the West of Ireland. Eur Heart J 2008; 29(11):1418-23.
20	Morris VB, Keelan T, Leen E, et al. Sudden cardiac death in the young: a 1-year post-mortem analysis in the Republic of Ireland. Ir J Med Sci 2009; 178(3):257-61.
21	Sheikhazadi A, Gharehdaghi J. Survey of sudden death from aneurysmal

	subarachnoid hemorrhage in cadavers referred to Legal Medicine Organization of Tehran, 2001-2005. Am J Forensic Med Pathol 2009; 30(4):358-61.
22	Adabag AS, Peterson G, Apple FS, Titus J, King R, Luepker RV. Etiology of sudden death in the community: results of anatomical, metabolic, and genetic evaluation. Am Heart J 2010; 159(1):33-9.
23	Fragkouli K, Vougiouklakis T. Sudden cardiac death: an 11-year postmortem analysis in the region of Epirus, Greece. Pathol Res Pract 2010; 206(10):690-4.
24	Margey R, Roy A, Tobin S, et al. Sudden cardiac death in 14- to 35-year olds in Ireland from 2005 to 2007: a retrospective registry. Europace 2011; 13(10):1411-8.
25	Rao BH, Sastry BK, Chugh SS, et al. Contribution of sudden cardiac death to total mortality in India - a population based study. Int J Cardiol 2012; 154(2):163-7.
26	Morentin B, Suárez-Mier MP, Aguilera B, Arrieta J, Audicana C, Fernández-Rodríguez A. Clinicopathological features of sudden unexpected infectious death: population-based study in children and young adults. Forensic Sci Int 2012; 220(1-3):80-4.
27	Havmoeller R, Reinier K, Teodorescu C, et al. Low rate of secondary prevention ICDs in the general population: multiple-year multiple-source surveillance of sudden cardiac death in the Oregon Sudden Unexpected Death Study. J Cardiovasc Electrophysiol 2013; 24(1):60-5.
28	Maron BJ, Haas TS, Ahluwalia A, Rutten-Ramos SC. Incidence of cardiovascular sudden deaths in Minnesota high school athletes. Heart Rhythm 2013; 10(3):374-7.
29	Suárez-Mier MP, Aguilera B, Mosquera RM, Sánchez-de-León MS. Pathology of sudden death during recreational sports in Spain. Forensic Sci Int 2013; 226(1-3):188-96.

30	Pilmer CM, Porter B, Kirsh JA, et al. Scope and nature of sudden cardiac death before age 40 in Ontario: a report from the cardiac death advisory committee of the office of the chief coroner. Heart Rhythm 2013; 10(4):517-23.
31	Nagata M, Ninomiya T, Doi Y, et al. Temporal trends in sudden unexpected death in a general population: the Hisayama study. Am Heart J 2013; 165(6):932-938.e1.
32	Yang J, Teehan D, Farioli A, Baur DM, Smith D, Kales SN. Sudden cardiac death among firefighters ≤45 years of age in the United States. Am J Cardiol 2013; 112(12):1962-7.
33	Pilmer CM, Kirsh JA, Hildebrandt D, Krahn AD, Gow RM. Sudden cardiac death in children and adolescents between 1 and 19 years of age. Heart Rhythm 2014; 11(2):239-45.
34	Winkel BG, Risgaard B, Sadjadieh G, Bundgaard H, Haunsø S, Tfelt-Hansen J. Sudden cardiac death in children (1-18 years): symptoms and causes of death in a nationwide setting. Eur Heart J 2014; 35(13):868-75.
35	de Noronha SV, Behr ER, Papadakis M, et al. The importance of specialist cardiac histopathological examination in the investigation of young sudden cardiac deaths. Europace 2014; 16(6):899-907.
36	Wang H, Yao Q, Zhu S, et al. The autopsy study of 553 cases of sudden cardiac death in Chinese adults. Heart Vessels 2014; 29(4):486-95.
37	Hofer F, Fellmann F, Schläpfer J, Michaud K. Sudden cardiac death in the young (5-39 years) in the canton of Vaud, Switzerland. BMC Cardiovasc Disord 2014; 14:140.
38	Silva AC, Santos L, Dinis-Oliveira RJ, Magalhães T, Santos A. Sudden cardiac death in young adult. Cardiovasc Toxicol 2014; 14(4):379-86.
39	Martens E, Sinner MF, Siebermair J, et al. Incidence of sudden cardiac death in Germany: results from an emergency medical service registry in Lower

	Saxony. Europace 2014; 16(12):1752-8.
40	Zhang L, Narayanan K, Suryadevara V, et al. Occupation and risk of sudden death in a United States community: a case-control analysis. BMJ Open 2015; 5(12):e009413.
41	Feng JL, Hickling S, Nedkoff L, et al. Sudden cardiac death rates in an Australian population: a data linkage study. Aust Health Rev 2015; 39(5):561-567.
42	Niemeijer MN, van den Berg ME, Leening MJ, et al. Declining incidence of sudden cardiac death from 1990-2010 in a general middle-aged and elderly population: The Rotterdam Study. Heart Rhythm 2015; 12(1):123-9.
43	Braggion-Santos MF, Volpe GJ, Pazin-Filho A, Maciel BC, Marin-Neto JA, Schmidt A. Sudden cardiac death in Brazil: a community-based autopsy series (2006-2010). Arq Bras Cardiol 2015; 104(2):120-7.
44	Zheng J, Huang E, Tang S, et al. A case-control study of sudden unexplained nocturnal death syndrome in the southern Chinese Han population. Am J Forensic Med Pathol 2015; 36(1):39-43.
45	Risgaard B, Lynge TH, Wissenberg M, et al. Risk factors and causes of sudden noncardiac death: A nationwide cohort study in Denmark. Heart Rhythm 2015; 12(5):968-74.
46	Toukola T, Hookana E, Junttila J, et al. Sudden cardiac death during physical exercise: Characteristics of victims and autopsy findings. Ann Med 2015; 47(3):263-8.
47	Naneix AL, Périer MC, Beganton F, Jouven X, Lorin de la Grandmaison G. Sudden adult death: an autopsy series of 534 cases with gender and control comparison. J Forensic Leg Med 2015; 32:10-15.
48	Farioli A, Christophi CA, Quarta CC, Kales SN. Incidence of sudden cardiac death in a young active population. J Am Heart Assoc 2015; 4(6):e001818.
49	Harmon KG, Asif IM, Maleszewski JJ, et al. Incidence, Cause, and Comparative Frequency of Sudden Cardiac Death in National Collegiate

	Athletic Association Athletes: A Decade in Review. Circulation 2015; 132(1):10-9.
50	Chappex N, Schlaepfer J, Fellmann F, Bhuiyan ZA, Wilhelm M, Michaud K. Sudden cardiac death among general population and sport related population in forensic experience. J Forensic Leg Med 2015; 35:62-8.
51	Zhao P, Wang JG, Gao P, Li X, Brewer R. Sudden unexpected death from natural diseases: Fifteen years' experience with 484 cases in Seychelles. J Forensic Leg Med 2016; 37:33-8.
52	Smallman DP, Webber BJ, Mazuchowski EL, Scher AI, Jones SO, Cantrell JA. Sudden cardiac death associated with physical exertion in the US military, 2005-2010. Br J Sports Med 2016; 50(2):118-23.
53	Wu Q, Zhang L, Zheng J, et al. Forensic Pathological Study of 1656 Cases of Sudden Cardiac Death in Southern China. Medicine (Baltimore) 2016; 95(5):e2707.
54	Bohm P, Scharhag J, Meyer T. Data from a nationwide registry on sports-related sudden cardiac deaths in Germany. Eur J Prev Cardiol 2016; 23(6):649-56.
55	Maron BJ, Haas TS, Duncanson ER, Garberich RF, Baker AM, Mackey-Bojack S. Comparison of the Frequency of Sudden Cardiovascular Deaths in Young Competitive Athletes Versus Nonathletes: Should We Really Screen Only Athletes?. Am J Cardiol 2016; 117(8):1339-41.
56	Finocchiaro G, Papadakis M, Robertus JL, et al. Etiology of Sudden Death in Sports: Insights From a United Kingdom Regional Registry. J Am Coll Cardiol 2016; 67(18): 2108-15.
57	Vassalini M, Verzeletti A, Restori M, De Ferrari F. An autopsy study of sudden cardiac death in persons aged 1-40 years in Brescia (Italy). J Cardiovasc Med (Hagerstown) 2016; 17(6):446-53.
58	Parsons S, Lynch M. Sudden cardiac death while playing Australian Rules football: a retrospective 14 year review. Forensic Sci Med Pathol 2016;

	12(2):158-62.
59	Bagnall RD, Weintraub RG, Ingles J, et al. A Prospective Study of Sudden Cardiac Death among Children and Young Adults. N Engl J Med 2016; 374(25):2441-52.
60	Bogle BM, Ning H, Mehrotra S, Goldberger JJ, Lloyd-Jones DM. Lifetime Risk for Sudden Cardiac Death in the Community. J Am Heart Assoc 2016; 5(7):e002398.
61	Gräni C, Chappex N, Fracasso T, et al. Sports-related sudden cardiac death in Switzerland classified by static and dynamic components of exercise. Eur J Prev Cardiol 2016; 23(11):1228-36.
62	Kim AS, Moffatt E, Ursell PC, Devinsky O, Olgin J, Tseng ZH. Sudden neurologic death masquerading as out-of-hospital sudden cardiac death. Neurology 2016; 87(16):1669-1673.
63	Harmon KG, Asif IM, Maleszewski JJ, et al. Incidence and Etiology of Sudden Cardiac Arrest and Death in High School Athletes in the United States. Mayo Clin Proc 2016; 91(11):1493-1502.
64	Risgaard B. Sudden cardiac death: a nationwide cohort study among the young. Dan Med J 2016; 63(12):B5321.
65	Ifteni P, Barabas B, Gavris C, Moga M, Burtea V, Dracea L. Sudden Cardiac Death: Autopsy Findings in 7200 Cases Between 2001 and 2015. Am J Forensic Med Pathol 2017; 38(1):49-53.
66	Krexi D, Sheppard MN. Cardiovascular causes of maternal sudden death. Sudden Arrhythmic Death Syndrome is leading cause in UK. Eur J Obstet Gynecol Reprod Biol 2017; 212:177.
67	Feng JL, Nedkoff L, Knuiman M, et al. Temporal Trends in Sudden Cardiac Death From 1997 to 2010: A Data Linkage Study. Heart Lung Circ 2017; 26(8):808-816.
68	Wisten A, Krantz P, Stattin EL. Sudden cardiac death among the young in Sweden from 2000 to 2010: an autopsy-based study. Europace 2017;

	19(8):1327-1334.
69	Bonny A, Tibazarwa K, Mbouh S, et al. Epidemiology of sudden cardiac death in Cameroon: the first population-based cohort survey in sub-Saharan Africa. Int J Epidemiol 2017; 46(4):1230-1238.
70	Ding Z, Yang M, Wang Y, Wu S, Qiu X, Liu Q. Retrospective analysis of 769 cases of sudden cardiac death from 2006 to 2015: a forensic experience in China. Forensic Sci Med Pathol 2017; 13(3):336-341.
71	Finocchiaro G, Papadakis M, Dhutia H, et al. Obesity and sudden cardiac death in the young: Clinical and pathological insights from a large national registry. Eur J Prev Cardiol 2018; 25(4):395-401.
72	Anastasakis A, Papatheodorou E, Ritsatos K, et al. Sudden unexplained death in the young: epidemiology, aetiology and value of the clinically guided genetic screening. Europace 2018; 20(3):472-480.
73	Jayaraman R, Reinier K, Nair S, et al. Risk Factors of Sudden Cardiac Death in the Young: Multiple-Year Community-Wide Assessment. Circulation 2018; 137(15):1561-1570.
74	Tseng ZH, Olgin JE, Vittinghoff E, et al. Prospective Countywide Surveillance and Autopsy Characterization of Sudden Cardiac Death: POST SCD Study. Circulation 2018; 137(25):2689-2700.
75	Drezner JA, Peterson DF, Siebert DM, et al. Survival After Exercise-Related Sudden Cardiac Arrest in Young Athletes: Can We Do Better?. Sports Health 2019; 11(1):91-98.
76	Ripoll T, García AB, Gomila I, et al. Post-mortem toxicology in the diagnosis of sudden death in young and middle-aged victims. Eur Rev Med Pharmacol Sci 2019; 23(21):9135-9149.
77	Zhang J, Zhou X, Xing Q, et al. Epidemiological investigation of sudden cardiac death in multiethnic Xinjiang Uyghur autonomous region in Northwest China. BMC Public Health 2019; 19(1):116. Published 2019 Jan 28.

78	Haukilahti MAE, Holmström L, Vähätalo J, et al. Sudden Cardiac Death in Women. Circulation 2019; 139(8):1012-1021.
79	Shuvy M, Qiu F, Lau G, et al. Temporal trends in sudden cardiac death in Ontario, Canada. Resuscitation 2019; 136:1-7.
80	Brodie LR, Odell M, Ranson D, Young C, Kitching F, Ibrahim JE. Sudden natural death behind the wheel: Review of driver deaths and fitness to drive assessment history in Victoria, Australia 2012-2013. J Forensic Leg Med 2019; 63:31-33.
81	Srettabunjong S. Sudden Unexplained Nocturnal Death Syndrome: Epidemiological and Morphological Characteristics in Thai Autopsy Cases. J Forensic Sci 2019; 64(3):773-777.
82	Morentin B, Callado LF. Sudden cardiac death associated to substances of abuse and psychotropic drugs consumed by young people: A population study based on forensic autopsies. Drug Alcohol Depend 2019; 201:23-28.
83	Joodi G, Maradey JA, Bogle B, et al. Coronary Artery Disease and Atherosclerotic Risk Factors in a Population-Based Study of Sudden Death. J Gen Intern Med 2020; 35(2):531-537.
84	Sheppard MN, Westaby J, Zullo E, Fernandez BVE, Cox S, Cox A. Sudden arrhythmic death and cardiomyopathy are important causes of sudden cardiac death in the UK: results from a national coronial autopsy database. Histopathology 2023; 82(7):1056-1066.
85	Belhaj A, Shimi M, Kort I, Zaara MA, Hamdoun M, Ben Khelil M. Risk factors of sudden cardiac death in women: A 10 years study in Tunisia. J Forensic Leg Med 2023; 96:102517.
86	Puolitaival E, Vähätalo J, Holmström L, et al. Causes and characteristics of unexpected sudden cardiac death in octogenarians/nonagenarians. PLoS One 2023; 18(4):e0284515.
87	Finocchiaro G, Radaelli D, D'Errico S, et al. Sudden Cardiac Death Among Adolescents in the United Kingdom. J Am Coll Cardiol 2023;

	81(11):1007-1017.
88	Stattin EL, Hagström E, Dahl N, et al. Cohort profile: the Swedish study of SUDden cardiac Death in the Young (SUDDY) 2000-2010: a complete nationwide cohort of SCDs. BMJ Open 2022; 12(5):e055557.
89	Frontera A, Anselmino M, Matta M, et al. Ante-mortem characterization of sudden deaths as first-manifestation in Italy. J Interv Card Electrophysiol 2022; 63(2):267-274.
90	Rücklová K, Dobiáš M, Bílek M, et al. Burden of sudden cardiac death in persons aged 1-40 years in the Czech Republic. Cent Eur J Public Health 2022; 30(1):58-64.
91	Colantonio LD, Reynolds RJ, Merriman TR, et al. Higher Serum Urate Levels Are Associated With an Increased Risk for Sudden Cardiac Death. J Rheumatol 2021; 48(11):1745-1753.
92	Işın A, Turgut A, Peden AE. Epidemiology of Football-Related Sudden Cardiac Death in Turkey. Medicina (Kaunas) 2021; 57(10):1105.
93	Guo L, Torii S, Fernandez R, et al. Genetic Variants Associated With Unexplained Sudden Cardiac Death in Adult White and African American Individuals. JAMA Cardiol 2021; 6(9):1013-1022.
94	Ripoll-Vera T, Pérez Luengo C, Borondo Alcázar JC, et al. Sudden cardiac death in persons aged 50 years or younger: diagnostic yield of a regional molecular autopsy program using massive sequencing. Rev Esp Cardiol (Engl Ed) 2021; 74(5):402-413.
95	Saadi S, Ben Jomaa S, Bel Hadj M, Oualha D, Haj Salem N. Sudden death in the young adult: a Tunisian autopsy-based series. BMC Public Health 2020; 20(1):1915.
96	Ha FJ, Han HC, Sanders P, et al. Sudden Cardiac Death in the Young: Incidence, Trends, and Risk Factors in a Nationwide Study. Circ Cardiovasc Qual Outcomes 2020; 13(10):e006470.
97	Chen PH, Tsai SY, Pan CH, et al. Incidence and risk factors of sudden

	cardiac death in bipolar disorder across the lifespan. J Affect Disord 2020; 274:210-217.
98	Park YM, Moon J, Hwang IC, Lim H, Cho B. Short stature is associated with incident sudden cardiac death in a large Asian cohort. Heart Rhythm 2020; 17(6):931-936.
99	Chang RK, Keens TG, Rodriguez S, Chen AY. Sudden infant death syndrome: changing epidemiologic patterns in California 1989-2004. J Pediatr 2008; 153(4):498-502.
100	Liebrechts-Akkerman G, Bovee JV, Wijnaendts LC, Maes A, Nikkels PG, de Krijger RR. Histological findings in unclassified sudden infant death, including sudden infant death syndrome. Pediatr Dev Pathol 2013; 16(3): 168-76.
101	Malloy MH. Prematurity and sudden infant death syndrome: United States 2005-2007. J Perinatol 2013; 33(6):470-5.
102	Evans A, Bagnall RD, Duflou J, Semsarian C. Postmortem review and genetic analysis in sudden infant death syndrome: an 11-year review. Hum Pathol 2013; 44(9): 1730-6.
103	Hakeem GF, Oddy L, Holcroft CA, Abenhaim HA. Incidence and determinants of sudden infant death syndrome: a population-based study on 37 million births. World J Pediatr 2015; 11(1):41-7.
104	Tuchtan L, Delteil C, Levrat F, et al. Sudden unexpected infant death characteristics in the French region of West Provence-Alpes-Côte d'Azur. Paediatr Int Child Health 2019; 39(2):104-110.
105	Drake SA, Wolf DA, Yang Y, et al. A Descriptive and Geospatial Analysis of Environmental Factors Attributing to Sudden Unexpected Infant Death. Am J Forensic Med Pathol 2019; 40(2):108-116.
106	Mitchell EA, Taylor BJ, Milne BJ. Regional variation in sudden unexpected death in infancy in New Zealand. J Paediatr Child Health 2023; 59(2):319-327.

107	Bartick M, Barr AW, Feldman-Winter L, Guxens M, Tiemeier H. The Role of Breastfeeding in Racial and Ethnic Disparities in Sudden Unexpected Infant Death: A Population-Based Study of 13 Million Infants in the United States. Am J Epidemiol 2022; 191(7):1190-1201.
108	Anderson TM, Allen K, Ramirez JM, Mitchell EA. Circadian variation in sudden unexpected infant death in the United States. Acta Paediatr 2021; 110(5): 1498-504.
